# Myocardial Scar Assessment Using Artificial Intelligence-Powered Contrast-Free MRI: A Prospective Multicenter Study of Virtual Native Enhancement

**DOI:** 10.1016/j.jacc.2026.05.029

**Published:** 2026-08-18

**Authors:** Qiang Zhang, Di Zhou, Patrick Thompson, Georgios Vavillis, Thomas Anderton, Rajib Ahmed, Ricardo A. Gonzales, Huaying Zhang, Wai Yan Ryana Fok, Daniel Atkinson, Katharine E. Thomas, Andrew Lewis, Oliver Rider, Keith M. Channon, Stefan Neubauer, Sven Plein, Minjie Lu, Peter P. Swoboda, Stefan K. Piechnik, Vanessa M. Ferreira

**Affiliations:** a Division of Cardiovascular Medicine, Radcliffe Department of Medicine, University of Oxford, Oxford, United Kingdom; b Big Data Institute, Li Ka Shing Centre for Health Information and Discovery, University of Oxford, Oxford, United Kingdom; c Department of Magnetic Resonance Imaging, Fuwai Hospital, State Key Laboratory of Cardiovascular Disease, National Centre for Cardiovascular Diseases, Chinese Academy of Medical Sciences and Peking Union Medical College, Beijing, China; d Department of Biomedical Imaging Science, Leeds Institute of Cardiovascular and Metabolic Medicine, University of Leeds, Leeds, United Kingdom

**Keywords:** artificial intelligence, cardiovascular magnetic resonance, gadolinium-free, myocardial tissue characterization, virtual native enhancement

## Abstract

**Background:**

Myocardial scar detection using cardiovascular magnetic resonance (CMR) may now be achieved without contrast agents using artificial intelligence virtual native enhancement (VNE); prospective multicenter clinical validation is needed to establish its feasibility for routine clinical use.

**Objectives:**

The aim of this study was to prospectively investigate the diagnostic performance of VNE in myocardial scar detection using a multicenter, blinded approach in real-world clinical settings.

**Methods:**

CMR data sets were prospectively collected from 2 sites (Leeds and Fuwai). Leeds and Fuwai teams independently determined scar presence on late gadolinium enhancement (LGE) as ground truth. The Oxford team generated VNE images using only precontrast cine and T1 map images from the other 2 sites. The Oxford team scored VNE images blinded to clinical data and LGE. Four additional clinical readers blindly assessed the matched-slice VNE and LGE images.

**Results:**

VNE diagnosis of myocardial infarction achieved 94.4% accuracy on the basis of confident images (n = 107) and 87.5% accuracy on all images (n = 136). Myocardial scar quantification by VNE correlated strongly with LGE (*R* = 0.90) and agreed closely with LGE (mean difference 3.2%; 95% CI: −10.4% to +16.8%). VNE-LGE agreement on infarcted myocardial territories was 90.0%. The 4 clinical readers considered VNE adequate without proceeding to LGE in 69.7% of patients, and in these cases, VNE achieved an average diagnostic accuracy of 93.7%, comparable with that of LGE (93.9%).

**Conclusions:**

Accurate myocardial scar quantification can be achieved without contrast agents in high-quality and clear VNE images. VNE may triage the need for LGE and obviate its use in more than two-thirds of referrals for chronic myocardial infarction scar assessment without compromising diagnostic accuracy.

Myocardial scar assessment is important in ischemic and other cardiac conditions for clinical management and prognosis.^1^ The current imaging gold standard for assessing myocardial infarction (MI) and fibrosis is cardiovascular magnetic resonance (CMR) late gadolinium enhancement (LGE) imaging,^2^ which is now routinely used in clinical practice. The presence and extent of LGE provide critical information about myocardial scar burden and viability and guide decision-making regarding revascularization, risk stratification, and prognosis.^3^^,^^4^ However, LGE requires intravenous administration of gadolinium-based contrast agents (GBCAs), which increases scan time and costs and may be contraindicated in some patients. Consequently, there is growing interest in developing contrast-free imaging to improve the throughput and accessibility of CMR for general patient care.

Myocardial scar imaging may now be achieved without contrast agents in CMR using artificial intelligence (AI)–powered innovations, such as virtual native enhancement (VNE). VNE combines precontrast images (such as cine and T1 maps) and then uses generative AI models to enhance the intrinsic signals in these images, to simulate LGE images, without the need to inject contrast.^5^^,^^6^ VNE has demonstrated feasibility in hypertrophic cardiomyopathy and chronic MI.^5^^,^^6^

However, despite their transformative potential, the clinical translation of AI-based solutions is hindered by challenges in generalizability, reproducibility, quality control, reliability, and a lack of evidence based on prospective, blinded assessments in real-world clinical settings, which collectively represent standards that many AI innovations have failed to achieve to demonstrate clinical robustness. For VNE, a prospective, multicenter validation study at independent centers was crucial to establish its potential for clinical adoption.

In this study, we significantly advanced an AI-based technology (VNE) to the next critical stage of clinical translation for myocardial scar assessment. We addressed several key challenges, namely, multicenter generalizability, reproducibility, and quality control in real-world clinical applications, in which most AI technologies struggle or fail. We undertook the first prospective, truly blinded, multicenter validation of a generative AI approach for contrast-free myocardial scar assessment using CMR. Additionally, this work established a translational framework for deploying AI-enabled imaging across clinical imaging centers. We evaluated this technology in 2 common clinical cohorts: patients referred for suspected ischemic heart disease and viability assessment, which account for a substantial proportion of routine CMR referrals.

This work sets standards and provides an evidence-based roadmap for clinical validation and implementation of AI-generated diagnostic imaging, which are relevant to all physicians who use cardiac imaging that increasingly incorporate AI solutions for patient care (Central Illustration).

**Central Illustration:**
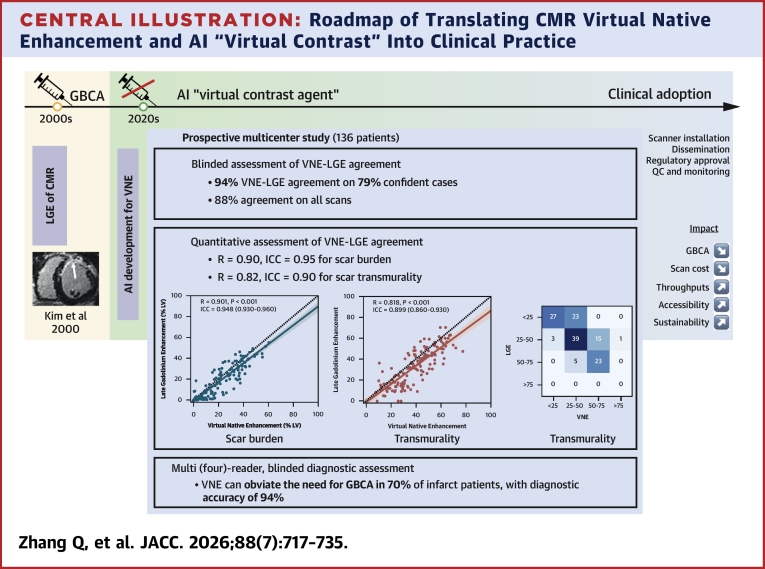
Roadmap of Translating CMR Virtual Native Enhancement and AI “Virtual Contrast” Into Clinical Practice AI = artificial intelligence; CMR = cardiovascular magnetic resonance; GBCA = gadolinium-based contrast agent; ICC = intraclass correlation coefficient; LGE = late gadolinium enhancement; QC = quality control; VNE = virtual native enhancement.

## Methods

### Study population and protocol

This study uses materials prospectively acquired at the Leeds Institute of Cardiovascular and Metabolic Medicine in the United Kingdom (Leeds site) and at Fuwai Hospital, National Centre for Cardiovascular Diseases, in China (Fuwai site). Both sites have ethics approval to undertake this research. All patients provided informed consent. The LGE and VNE materials are available on reasonable request to Leeds and Fuwai study investigators.

The VNE scan protocol includes 3 short-axis slices of precontrast T1 mapping (shortened modified Look-Locker inversion recovery),^7^ precontrast steady-state free precession cine imaging, and postcontrast motion-corrected LGE imaging, with matching slice positions between T1, cine, and LGE. At the beginning of this study, T1 mapping sequences were quality assured using a phantom approach^8^ (Supplemental Appendix).

The Leeds site prospectively and consecutively recruited patients referred clinically for the investigation of ischemia. These patients may or may not have had previous MI. The Fuwai site prospectively and selectively recruited patients with previous diagnoses of MI undergoing percutaneous coronary intervention who were referred for new symptoms of chest pain. LGE extent, pattern, and location were not known during enrollment.

The Leeds site scanned patients using a 1.5-T MAGNETOM Aera scanner (software version syngo MR E11, Siemens Healthineers). LGE was acquired 6 to 10 minutes after the injection of Gadovist (gadobutrol 0.1 mmol/kg, Bayer Healthcare). The Fuwai site scanned patients using a 3-T Skyra scanner (software version syngo MR E11, Siemens Healthineers). LGE was acquired 10 minutes after the injection of Magnevist (gadopentetate dimeglumine 0.1mmol/kg, Bayer Healthcare).

### VNE image generation

VNE used a generative AI model to combine precontrast cine imaging, T1 maps, and the underlying T1-weighted images, enhancing intrinsic magnetic resonance signals, to produce virtual LGE-like images without using extrinsic contrast media. As detailed in its original development, the VNE AI model was trained on 842 patient materials acquired at a single center, consisting of CMR scans obtained on 1.5- and 3-T Siemens scanners, from patients with confirmed histories of prior MI without substantial concomitant myocardial pathology, as well as from patients undergoing 6-month follow-up after acute MI.^6^ The training cohort had an average age of 64 years, included 81% men, and had an average infarct age of 7 months.^6^

**Figure 1:**
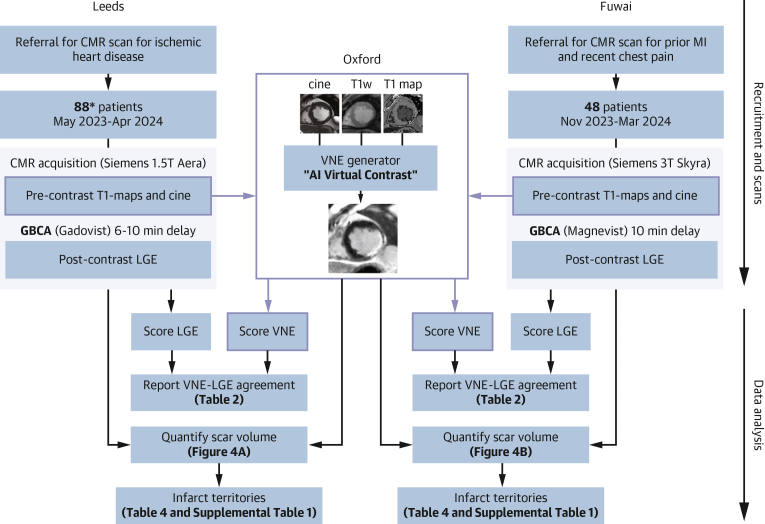
Study Design and Data Analysis for Prospective Validation of VNE The Leeds and Fuwai sites prospectively scanned patients. The Oxford team generated virtual native enhancement (VNE) images from precontrast images, blinded to postcontrast late gadolinium enhancement (LGE) and clinical information. Qualitative and quantitative analyses were subsequently performed to assess VNE-LGE agreement. Outlined boxes illustrate the generation and scoring of VNE blinded to postcontrast images and information. ∗Five patient materials were excluded due to absence of matched cine (n = 2) and severe image artifacts (n = 3), resulting in 88 patient scans for inclusion in the study. AI = artificial intelligence; CMR = cardiovascular magnetic resonance; GBCA = gadolinium-based contrast agent; MI = myocardial infarction; T1w = T1-weighted.

In the present study we assessed the VNE technology at independent clinical centers in a blinded manner. VNE deployment software and pipeline were developed for this validation using TensorFlow and Python Tkinter, incorporating motion correction neural networks,^9^ VNE AI neural networks,^6^ and myocardial segmentation neural networks^10^ to process raw Digital Imaging and Communications in Medicine data. There was no additional tuning of the underpinned VNE AI model. For the Leeds cohort, only the anonymized precontrast cine images and T1 maps were shared with the Oxford team (Figure 1). The images were fed into the VNE pipeline to generate VNE images. For the Fuwai cohort, because of institutional restrictions on data export, the VNE software was deployed on site. A clinical researcher (D.Z.) used the software to generate VNE images from anonymized precontrast data, and a member of the Oxford development team (Q.Z.) subsequently visited the site to perform postprocessing of the VNE images on site (Figure 1). The Oxford team (including Q.Z.) did not have access to LGE images before blinded reader analysis.

### Blinded reader analysis of VNE diagnostic accuracy

The Oxford team analyzed the precontrast cine, T1 map, and VNE images blindly without having access to LGE or additional clinical information, all of which were safeguarded by the collaborating sites. The presence of MI on VNE images was scored by a CMR cardiologist (V.M.F., with 20 years’ experience, Society for Cardiovascular Magnetic Resonance level III reader), with no additional clinical information or images (such as perpendicular views). Patient cases, as well as individual short-axis slices, were classified as having clear-cut “definite infarct” or “no infarct”; the label “possible infarct” was given if the VNE showed a hyperintensity lesion but diagnostic certainty was affected by confounding signals from the left ventricular outflow tract, blurry signals, small areas of enhancement adjacent to the blood pool, and image artifacts. For patients with “definite infarct” or “possible infarct,” the myocardial territories of infarction were marked and categorized as “anterior” (including anterior and anteroseptal segments), “inferior” (including inferior and inferoseptal segments), and/or “lateral” (including anterolateral and inferolateral segments), corresponding to the left anterior descending coronary artery, right coronary artery, and left circumflex coronary artery territories in the American Heart Association’s 16-segment model, respectively.^11^ The Leeds team (P.T., P.P.S.) and Fuwai team (D.Z.) independently determined the ground truth by scar presence on the basis of all LGE images (including a full short-axis stack and orthogonal long-axis views) and health record, without access to VNE classifying the LGE as binary “no infarct” or “infarct,” and marking the myocardial territories of infarct. Findings of nonischemic diffuse LGE were also scored and recorded. All scores were locked before the Oxford site had access to LGE for subsequent quantification of scar burden.

### Matched-slice multireader blinded VNE-LGE comparison

This study acquired full short-axis stack LGE and provided VNE typically at basal, midventricular, and apical slice positions. To compare the head-to-head diagnostic performance of VNE and LGE, matched LGE slices were extracted from the full LGE stack. The LGE or VNE was then presented to clinical readers in random order and blinding their origin, with both VNE and LGE accompanied by the underpinning precontrast T1 maps and cine images. Using a dedicated user interface, 4 clinical readers (K.E.T., A.L., O.R., M.L.) scored for the presence of infarct, followed by a query of whether the reader would further request GBCA to obtain corresponding slices of LGE, if the images were contrast-free VNE. The query was designed to be answered in advance of revealing any results, to subjectively assess the accuracy of VNE on the confident subsets without post hoc adjustment. The readers also scored scar transmurality, using categorical ratings of <25%, 25% to 50%, 50% to 75%, and >75% (Supplemental Appendix). Quantitative analysis of scar burden in LGE and VNE images was also performed (Supplemental Appendix).

### Statistical analysis

Statistical data analysis was performed using Python version 3.9.6. The diagnostic accuracy of VNE against LGE was calculated on the basis of categorical observers’ scores. Correlation between scar size by VNE and LGE was assessed using linear least squares regression taking VNE as exposure variables and LGE as outcome variables, along with Pearson correlation coefficients and intraclass correlation coefficients (ICCs). A mixed-effects model was used to calculate the correlation coefficients in per-slice comparison to account for repeated measures per patient. For the intraclass correlation, we used a mixed-effects variance-component approach to compute ICC(3,k),^12^ retaining all image-level observations while incorporating a random intercept for each subject to account for within-subject clustering. Interobserver variability was assessed using the ICC and categorical Fleiss’s kappa. Bland-Altman analysis was performed to analyze any systematic differences between quantification by VNE and LGE. Area under the receiver-operating characteristic curve (AUC-ROC) and the Youden index (*J*) were calculated to compare VNE and LGE burden in discriminating between cohorts with and without infarct. Statistical significance was defined as *P* < 0.05.

## Results

### Study population

At the Leeds site, 93 patients underwent CMR with the specified protocol (cine, T1 mapping, and LGE), following clinical referral for the investigation of ischemia (May 2023 to April 2024). Five scans were excluded: 2 scans did not acquire matched cine and T1 map pairs, 1 had severe pacemaker lead artifact, 1 had severe T1 map motion artifact, and 1 had cine motion artifact with mistriggering. This resulted in 88 patient scans for inclusion from Leeds (Figure 1). At the Fuwai site, 48 patients who had histories of MI were recruited and scanned (November 2023 to March 2024), following clinical referral for investigation of chest pain. Scan quality was inspected at the time of acquisition, and there was no patient exclusion after VNE image generation, resulting in 48 patient scans from Fuwai. This provided a total of 136 patient cases (389 data sets with matched cine, T1 map, VNE, and LGE slices) for further analysis in this study. Patient demographics and characteristics of the patient groups are given in Table 1.

**Table 1:** Patient Characteristics of the Cardiovascular Magnetic Resonance Imaging Cohorts at Independent Testing Sites

	Leeds Patients (n = 93)	Fuwai Patients (n = 48)	All Patients (N = 141)
Age, y			
Mean ± SD	63.4 ± 12.1	58.0 ± 9.7	61.7 ± 11.5
Median (Q1-Q3)	64.0 (56.0-72.0)	58.5 (53.8-66.3)	68.5 (60.0-74.0)
Male	68 (73.1)	44 (91.7)	115 (78.2)
BMI, kg/m^2^	29.2 ± 5.3 (n = 60)	25.7 ± 4.6	27.8 ± 5.3 (N = 108)
Cardiovascular risk factors			
Diabetes mellitus	38 (40.9)	15 (31.3)	56 (38.1)
Hypertension	45 (48.4)	23 (47.9)	71 (48.3)
Hypercholesterolemia	21 (22.6)	39 (81.3)	62 (42.2)
Smoking history	13 (22.4) (n = 58)	30 (62.5)	43 (39.4) (N = 106)
eGFR <90 mL/min/1.73 m^2^	49 (68.1) (n= 72)	20 (50) (n = 40)	73 (62.4) (N = 112)
eGFR <60 mL/min/1.73 m^2^	13 (18.1) (n = 72)	3 (7.5) (n = 40)	17 (14.5) (N = 112)
Previous revascularization	44 (49.4) (n = 89)	40 (83.3)	86 (60.1) (N = 137)
Confirmed history of prior MI	39 (42.9) (n = 91)	48 (100)	89 (61.4) (N = 139)

Values are mean ± SD, median (Q1-Q3), or n (%). Patient characteristics for the Leeds and Fuwai cohorts, along with combined data. Actual number of patients for each characteristic reported in clinical and cardiovascular magnetic resonance scan records is in ( ).

BMI = body mass index; eGFR = estimated glomerular filtration rate; MI = myocardial infarction.

### Blinded reader analysis of VNE diagnostic accuracy

For all 136 cases (389 per-slice data sets), the blinded observer from Oxford reviewed VNE for subendocardial and transmural enhancement and its underpinning cine for abnormal wall motion and both T1 map and cine for image quality and artifacts. On a per-patient basis, on the basis of VNE images, 107 cases were confidently scored as having “definite infarct” or “no infarct.” In the remaining 29 cases, VNE showed enhancement suggesting infarct, but the signals were subtle or difficult to distinguish from left ventricular outflow tract, blood pool, or artifacts because of a limited number of short-axis slices and no complementary long-axis slices or clinical information; these VNE images were scored as “possible infarct” for reduced diagnostic confidence (Table 2). A full list of these images is provided in Supplemental Appendix.

**Table 2:** Intermethod Agreement Between VNE and LGE in Detecting the Presence of Myocardial Infarction Scars

	All Patients (N = 136)			Leeds Patients (n = 88)			Fuwai Patients (n = 48)		
	No Infarct (44)	Possible Infarct (29)	Definite Infarct (63)	No Infarct (44)	Possible Infarct (22)	Definite Infarct (22)	No Infarct (0)	Possible Infarct (7)	Definite Infarct (41)
No infarct	39	11	1^a^	39	11	1	0	0	0
Infarct	5^a^	18	62	5	11	21	0	7	41

**Figure 2:**
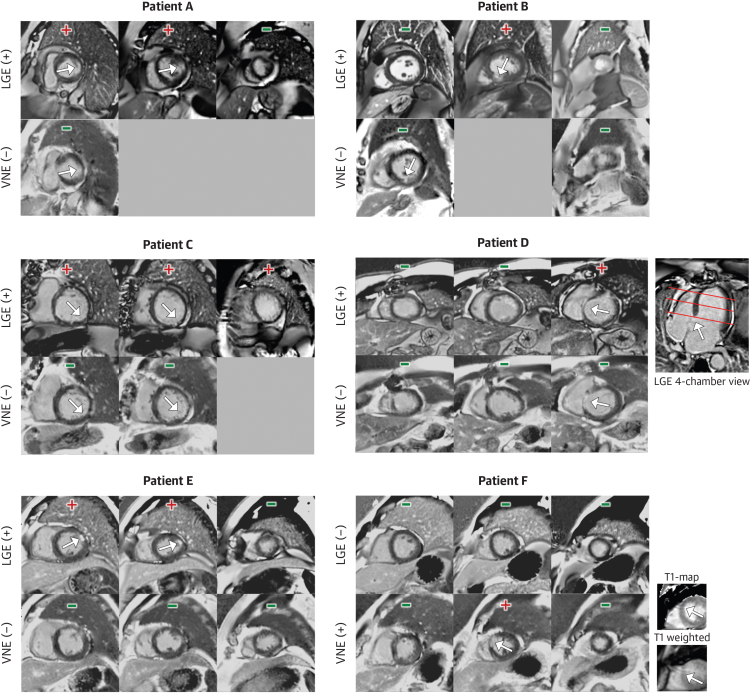
All 6 Cases With Discrepant LGE and VNE Scores for the Presence or Absence of MI Returned by Blinded, Independent Multiobserver Assessment Plus signs denote scored as infarct or possible infarct; minus signs denote scored as no infarct. Gray panels indicate missing image slices. (A) Discrepancy attributed to missing VNE slices. The single available VNE slice showed a small area of enhancement in the basal lateral wall, similar to LGE, but the VNE observer did not have the subsequent midventricular slice also showing a lateral wall scar to help diagnose a case of lateral MI. (B) Discrepancy attributed to missing VNE slice. The corresponding LGE slice showed an inferoseptal infarction. (C) Discrepancies attributed to interobserver variability in scoring MI. Both LGE and VNE showed thin subendocardial enhancement in the inferolateral wall but were interpreted differently. The LGE scorer would have had the entire short-axis stack and also long-axis LGE views to help recognize the presence of MI. (D) Left ventricular outflow tract on LGE basal slice was misinterpreted as scar, whereas the VNE scorer had attributed the enhancement to outflow tract partial volume effect. This was later confirmed on the 4-chamber long-axis LGE image. VNE and LGE images had good visual-spatial agreement. (E) A false negative VNE case: a small MI scar was missed on VNE. (F) A false positive VNE case: the VNE signal was later attributed to fat off-resonance artifact on the corresponding T1-map and T1-weighted images. Abbreviations as in Figure 1.

LGE = late gadolinium enhancement; VNE = virtual native enhancement.

a Disagreed cases (n = 6) are reported in Figure 2.

On the basis of the confident VNE images (107 cases), the per-patient accuracy of VNE in detecting the presence of MI was 94.4% (sensitivity 92.5%, specificity 97.5%, positive and negative predictive values 98.4% and 88.6%), using LGE as the ground truth (Table 3). Including the 29 cases with “possible infarct” on VNE (ie, a total of all 136 cases), the per-patient diagnostic accuracy of VNE dropped to 87.5% (sensitivity 94.1%, specificity 76.5%, positive predictive value 87.0%, negative predictive value 88.6%) (Table 3).

**Table 3:** VNE-LGE Agreement in Assessing the Presence of Myocardial Infarction Scar

	All Patients (N = 136)	Patients With Confident VNE Scores (n = 107)	Leeds Patients (n = 88)	Leeds Patients With Confident VNE Scores (n = 66)
Sensitivity, %	94.1 (88.80-96.98)	92.5 (85.91-96.15)	86.5 (77.82-92.13)	80.8 (69.71-88.50)
Specificity, %	76.5 (68.71-82.84)	97.5 (92.51-99.19)	76.5 (66.65-84.13)	97.5 (90.39-99.39)
PPV, %	87.0-(80.32-91.65)	98.4 (93.85-99.60)	72.7 (69.86-75.37)	95.5 (87.53-98.47)
NPV, %	88.6-(82.17-92.91)	88.6 (81.20-93.33)	88.6 (80.29-93.68)	88.6 (78.73-94.23)
Accuracy, %	87.5-(80.89-92.05)	94.4 (88.31-97.41)	80.7 (71.24-87.59)	90.9 (81.54-95.76)

Disagreements were attributed mainly to incomplete slice coverage of VNE and interobserver variability in scoring for small scars (Figure 2). The 95% CIs are reported in parentheses.

NPV = negative predictive value; PPV = positive predictive value; other abbreviations as in Table 2.

In the Leeds subset, the blinded observer was able to score 66 VNE cases as confidently having “definite infarct” or “no infarct” and 22 cases as “possible infarct” (Table 2). Using the 66 confident VNE cases, the diagnostic accuracy of VNE was 90.9%. Including the 22 cases with “possible infarct” on VNE, the diagnostic performance of VNE dropped to 80.7% (Table 3). In the Fuwai subset, of all 48 patients, the blinded observer scored 41 as “definite infarct” and 7 as “possible infarct” on the basis of VNE. All patients were confirmed on LGE images and health record as having infarct.

In the data sets with confident VNE scores (“definite infarct” and “no infarct,” excluding “possible infarct”), all individual patient cases of disagreement (n = 6 patients) are shown in Figure 2. In the first 4 patient cases (Figures 2A to 2D), there were no significant discrepancies in the VNE-LGE images and signals; discrepancies in diagnosing the presence and absence of MI are as follows. In patient A (Figure 2A), the VNE blinded observer only had 1 slice of VNE image showing subtle signals in the basal lateral wall, in contrast to the LGE observer, who had the whole short-axis stack and long-axis views of LGE to diagnose a lateral MI. In patient B (Figure 2B), the VNE image containing the infarction as shown on LGE was missing, precluding the VNE observer from diagnosing the MI. In patient C (Figure 2C), VNE images showed subtle subendocardial enhancement matching those in LGE, but were scored as “no infarct” because of difficulties in distinguishing them from blood pool. In patient D (Figure 2D), the outflow tract in the LGE basal slice was misinterpreted as scar, resulting in false positive LGE scores, as confirmed in the 4-chamber long-axis LGE image. Overall, full short-axis stack coverage and long-axis acquisitions for VNE would likely further improve its diagnostic confidence and accuracy.

**Figure 3:**
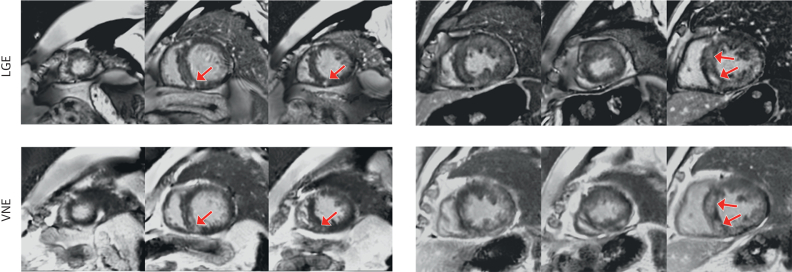
VNE Chronic MI Model Depicts Nonischemic Patchy Lesions in Agreement With LGE VNE captured scar patterns in agreement with LGE, as indicated by red arrows, suggesting a pathway of developing a generalist AI for CMR enhancement for common heart disease. Abbreviations as in Figure 1.

VNE missed a small lateral wall MI in 1 case (false negative), with more subtle enhancement in the area compared with the LGE image (Figure 2E). A false positive case was identified, which was attributed to the presence of fat off-resonance artifact on the corresponding T1 map and underpinning T1-weighted images (Figure 2F). Although this VNE model was trained only on ischemic LGE patterns, it also captured nonischemic patchy lesions, in good agreement with LGE, suggesting a pathway of developing generalist AI for CMR enhancement for common disease (Figure 3).

In the cases of “definite infarct” and “possible infarct,” the myocardial territories of infarction were further marked on the VNE (observer V.M.F.) and LGE (observers P.T., P.P.S., and D.Z.) images independently. The agreement between VNE and LGE scores on the territories of MI was 90.0% for all patient materials, 90.6% for Leeds patients, and 89.3% for Fuwai patients (Table 4). Disagreements were due mainly to the observers’ inclusion or exclusion of small infarctions in cases with multiterritory MIs (Supplemental Figure 1).

**Table 4:** VNE-LGE Agreement on Infarcted Myocardial Territories

	All Patients (N = 136)			Leeds Patients (n = 88)			Fuwai Patients (n = 48)		
	Anterior	Inferior	Lateral	Anterior	Inferior	Lateral	Anterior	Inferior	Lateral
Anterior	54	0	1	17	0	0	37	0	1
Inferior	4	13	1	2	5	1	2	8	0
Lateral	1	2	12	0	0	7	1	2	5

VNE-LGE agreement on infarcted myocardial territories was 90.0% for all patient materials, 90.6% for Leeds patients, 89.3% for Fuwai patients.

Abbreviations as in Table 2.

### Matched-slice multireader blinded VNE-LGE comparison

Of the 136 contrast-free VNE scans, the 4 clinical readers marked 87, 91, 94, and 107 cases as having adequate diagnostic quality, without further acquisition of LGE (Supplemental Appendix). In these cases, VNE diagnostic accuracy was 94.3%, 92.3%, 92.6%, and 95.3%, by the 4 readers, respectively, compared with LGE diagnostic accuracy of 95.4%, 90.1%, 93.5%, and 93.9%, with no statistically significant difference between the 2 methods (Table 5). This suggests that VNE can eliminate the need for LGE in 69.7% of patients without compromising diagnostic accuracy (average VNE accuracy of 93.7% vs LGE of 93.9%) (Table 5).

**Table 5:** Comparing the Diagnostic Performance of VNE and LGE Using the Matched Image Slices Assessed by 4 Clinical Readers

	VNE					LGE					n
	Sens, %	Spec, %	PPV, %	NPV, %	Acc, %	Sens, %	Spec, %	PPV, %	NPV, %	Acc, %	
All patients											
Reader 1	94.0	69.2	83.2	87.8	84.6	97.6	92.3	95.3	96	95.6	136
Reader 2	92.9	57.7	78	83.3	79.4	94	84.6	90.8	89.8	90.4	
Reader 3	96.4	50	75.7	89.7	78.7	96.4	80.8	89	93.3	90.4	
Reader 4	97.6	55.8	78.1	93.5	81.6	95.2	88.5	93	92	92.6	
Pooled	95.2	58.2	78.6	88.3	81.1	95.8	86.5	92	92.8	92.3	
Patients with VNE marked as adequate (ie, not requesting GBCA)											
Reader 1	96.5	90	94.8	93.1	94.3	96.5	93.3	96.5	93.3	95.4	87 (64.0%)
Reader 2	92.4	92	96.8	82.1	92.3	93.9	80	92.5	83.3	90.1	91 (66.9%)
Reader 3	98.6	76	91.9	95	92.6	100	88	95.8	100	96.8	94 (69.1%)
Reader 4	97.4	90.3	96.1	93.3	95.3	96.1	87.1	94.8	90	93.5	107 (78.7%)
Pooled	96.3	87.4	94.9	90.7	**93.7**	96.6	87.4	94.9	91.5	**93.9**	379 (69.7%)

VNE may obviate the need for GBCAs in more than two-thirds (69.7%) of patients for scar assessment, with average diagnostic accuracy of 93.7%, comparable with that achieved with LGE (93.9%). **Bold** numbers represent key summary findings. Further details are reported in Supplemental Appendix.

Acc = accuracy; GBCA = gadolinium-based contrast agent; Sens = sensitivity; Spec = specificity; other abbreviations as in Tables 2 and 3.

Using the full data set, without regard to confidence scoring (ie, including suboptimal VNE images scored as requiring further GBCA), VNE achieved a pooled accuracy of only 81.1%, compared with LGE accuracy of 92.3%; this highlights the critical importance of assessing the image quality of the precontrast imaging data (ie, cines and T1 maps) before using VNE for diagnosis. This may be performed either manually or with AI in the future that clearly indicates image quality and flags sources of artifacts for the reader, for more reliable diagnostic imaging when using AI-generated images such as VNE.

On the basis of visual assessment of matched image slices, VNE-LGE agreement in scar transmurality was ICC(3,k) between 0.89 and 0.93, with Pearson correlation coefficients of 0.81 to 0.86. Comparing the mean scores averaged across readers, the VNE-LGE agreement in scar transmurality was ICC = 0.94 and *R* = 0.88 (Supplemental Appendix).

Interobserver reproducibility among the 4 readers for scoring the presence of infarct was categorical Fleiss kappa = 0.718; for scoring scar transmurality, ICC = 0.915 (range: 0.882-0.942); for categorizing scar transmurality (>50% or <50%), Fleiss kappa = 0.770.

### Quantitative analysis of myocardial scar burden and transmurality

Myocardial scar quantification was performed on all 389 short-axis pairs of VNE and LGE images from 136 patients (Leeds, 245 short-axis pairs of images from 88 patients; Fuwai, 144 short-axis pairs of images from 48 patients). Contrast-to-noise ratio was 6.71 ± 2.93 (Leeds, 6.56 ± 2.70; Fuwai, 6.99 ± 3.29) by VNE, which was comparable with 6.76 ± 3.18 by LGE (Leeds, 6.55 ± 3.03; Fuwai, 7.09 ± 3.38).

**Figure 4:**
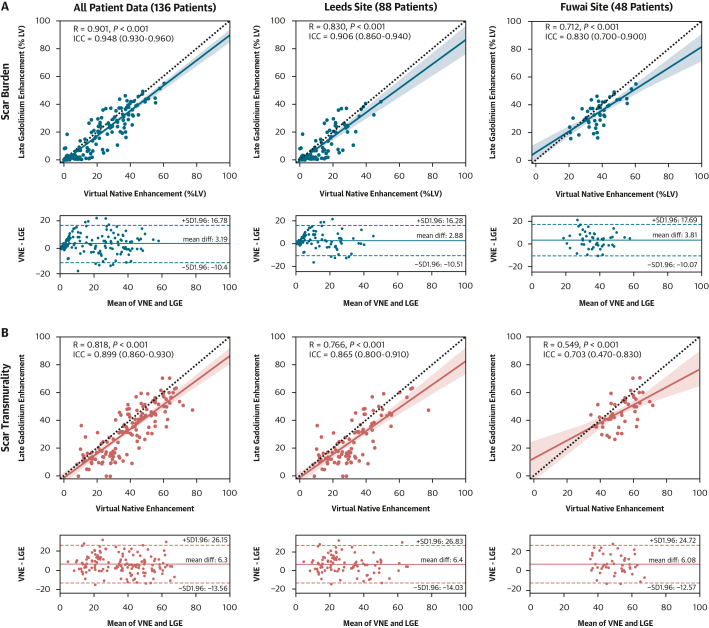
Assessment of Myocardial Scar Burden and Transmurality Using VNE in Comparison With LGE (A) VNE correlated strongly with LGE in quantifying scar burden as volume fraction of the left ventricular myocardium; the VNE-LGE difference was 3.2% in this cohort. (B) VNE correlated strongly with LGE in quantifying average scar transmurality. The VNE-LGE difference was 6.3%. There was no significant difference in the LGE-VNE agreement between the 2 cohorts (Supplemental Figure 4). ICC = intraclass correlation coefficient; LV = left ventricle; other abbreviations as in Figure 1.

Scar burden by VNE correlated strongly with LGE, with linear correlation coefficients of 0.90 (ICC = 0.95) for the entire cohort, 0.83 (ICC = 0.91) for Leeds, and 0.71 (ICC = 0.83) for Fuwai (Figure 4A).

Scar burden by VNE agreed closely with LGE. Bland-Altman plots showed that the mean VNE-LGE difference was 3.2% (95% CI: 17% to −10%). Calculated according to the distribution of scar burden, VNE-LGE agreement on the myocardial territories of infarction was 88.6% for the entire cohort (Leeds, 87.1%; Fuwai, 89.5%) (Supplemental Table 1).

**Figure 5:**
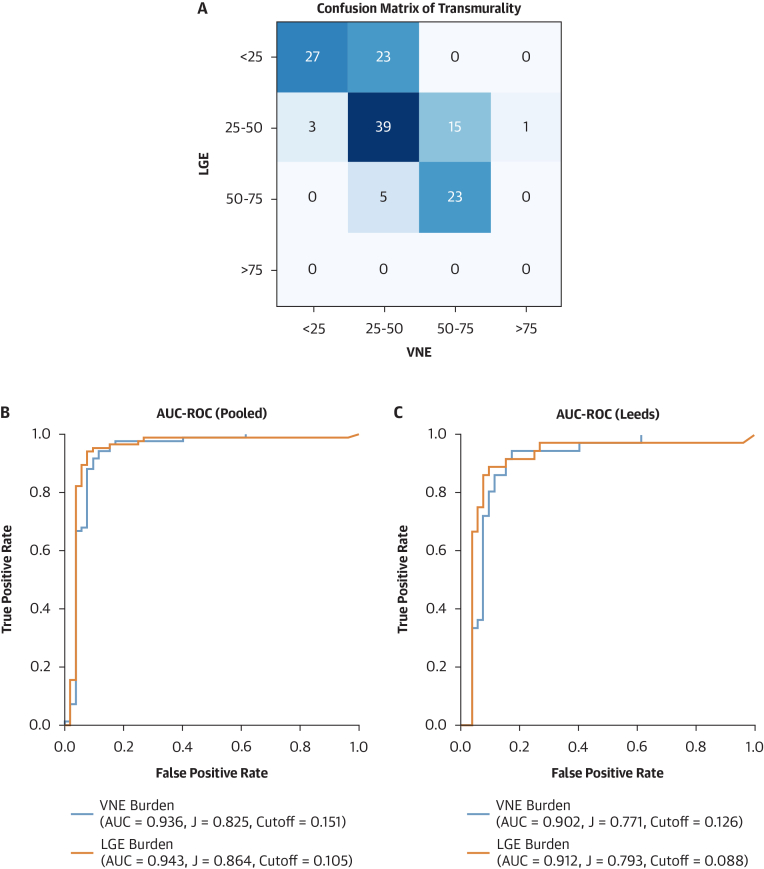
VNE-LGE Confusion Matrix of Transmurality and AUC-ROC (A) Using the full-width-at-half-maximum scar quantification method, VNE-LGE agreement on categorizing transmurality was 84.6% using a 50% transmurality cutoff and 65.4% using 25%, 50%, and 75% transmurality cutoffs. Color intensities are for visual guidance. In pooled cohorts (B) and the Leeds cohort (C), the areas under the receiver-operating characteristic curve (AUC-ROC) were 0.94 and 0.90 using VNE burden for classification and 0.94 and 0.91 using LGE burden. The numerically slightly higher AUC-ROC for LGE than VNE should be interpreted with a note of circular valuation bias, as LGE images was referenced in diagnosing infarction. The Youden index and its optimal cutoff were also reported. AUC = area under the receiver-operating characteristic curve; other abbreviations as in Figure 1.

Scar transmurality quantification by VNE also reported strong overall correlation (*R* = 0.82, ICC = 0.90) and agreement (mean difference: 6.3%; 95% CI: 26% to −14%) with LGE (Figure 4B). VNE-LGE agreement on categorizing transmurality was 84.6% using a 50% transmurality cutoff and 65.4% using <25%, 25% to 50%, 50% to 75%, and >75% transmurality cutoffs for the clinical assessment of subendocardial infarcts (Figure 5A).

In pooled cohorts (Figure 5B), the AUC-ROC was 0.94 (*J* = 0.83, sensitivity 0.94, cutoff at 15% scar burden) using VNE burden for classification and 0.94 (*J* = 0.86, sensitivity 0.94, cutoff at 11%) using LGE burden. In the Leeds cohort (Figure 5C), the AUC-ROC was 0.90 (*J* = 0.77, sensitivity 0.94, cutoff at 13%) using VNE burden for classification and 0.91 (*J* = 0.79, sensitivity 0.89, cutoff at 9%) using LGE burden. The slightly higher AUC-ROC for LGE than VNE should be interpreted with a note of circular valuation bias, as the same LGE images were directly referenced in ground-truth diagnostic labels of infarction.

**Figure 6:**
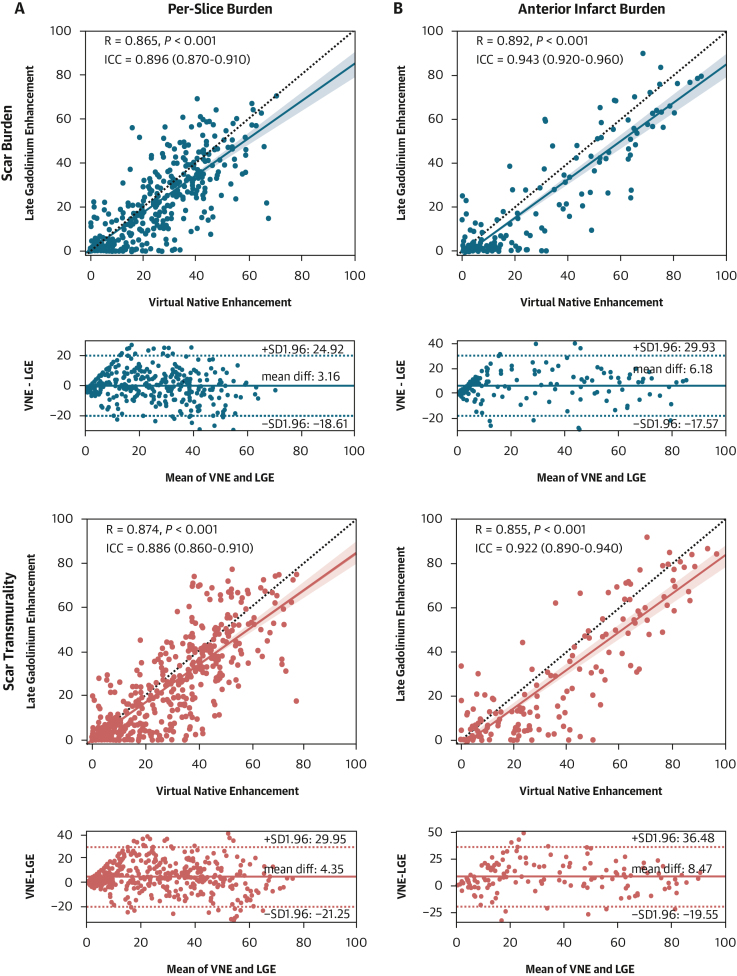

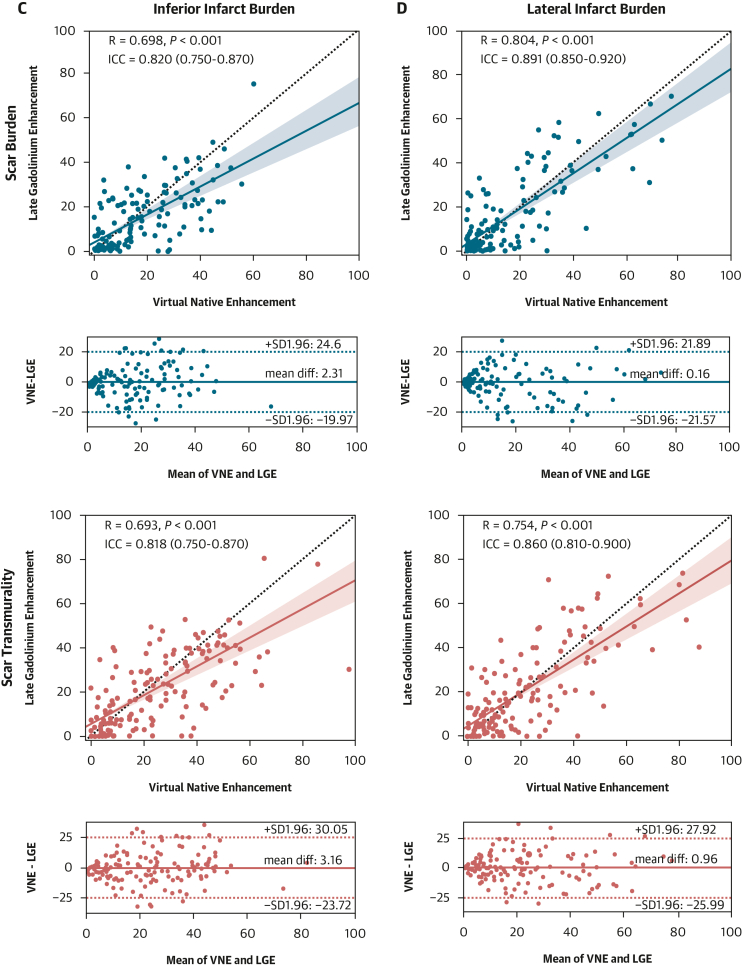
VNE-LGE Agreement for Scar Burden and Transmurality by Slice and Territory VNE demonstrated strong agreement with LGE in quantifying scar burden and transmurality per image slice (A) and across myocardial territories: anterior (B), inferior (C), and lateral (D). Abbreviations as in Figures 1 and 4.

Correlations of scar burden and transmurality on per-slice and per-myocardial-territory levels are further reported in Figure 6, to provide more insights into comparing the 2 methods. For scar burden, per-slice scar burden agreement was cluster-corrected *R* = 0.87 (ICC = 0.90); per-territory agreement was *R* = 0.70 to 0.89 (ICC = 0.82-0.94). For scar transmurality, per-slice agreement was cluster-corrected *R* = 0.87 (ICC = 0.89); per-territory agreement was *R* = 0.69-0.86 (ICC = 0.82-0.92).

Intraobserver reproducibility of the semiautomatic quantification was *R* = 0.96 (ICC = 0.96) for per-patient scar burden and *R* = 0.96 (ICC = 0.96) for per-patient transmurality. Intraobserver variabilities were ±1.96 SD intervals of 17% to 18%, accounting for approximately one-half of intermethod variability (Supplemental Figure 2).

Intermethod variability increased from the per-patient level (1.96 SD = ±0.14 and ±0.20 for scar burden and transmurality), to the per-territory level (1.96 SD = ±0.23 and ±0.28), and finally to the per-segment level (1.96 SD = ±0.41 and ±0.46) (Supplemental Figure 3). This reflects greater sensitivity of territory- and segment-level analyses to slice mismatch, patient motion, and the placement of insertion points.

**Figure 7:**
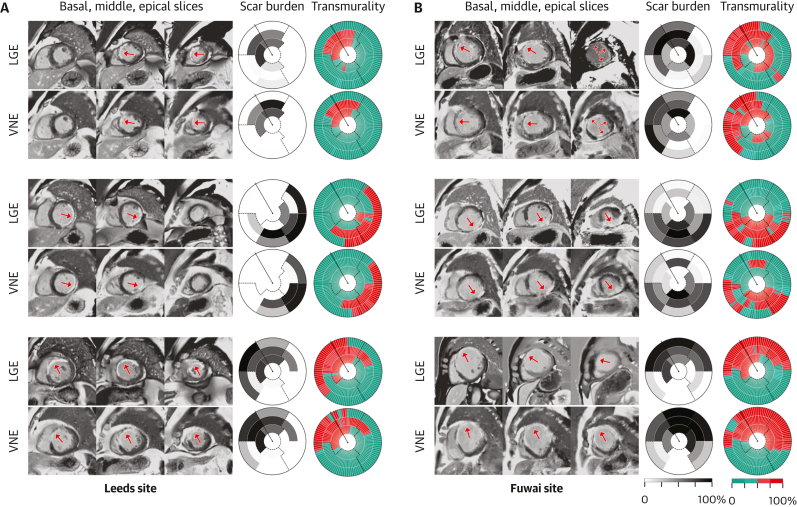
Representative LGE and VNE Images With Segmental Infarct Burden Representative images from 6 patients across the Leeds (A) and Fuwai (B) cohorts comparing LGE (top) and VNE (bottom). VNE accurately captured the presence, extent, location, and overall segmental distribution of infarct scars, as visualized with the bull’s-eye segmental model and transmurality plots (right). Abbreviations as in Figure 1.

Quantification and visualization following the 16-segment model captured and depicted MI in various myocardial territories, showing high visuospatial agreement between VNE and LGE in the presence, distribution, and extent of scars. Representative patient examples from the 2 cohorts are given in Figure 7.

**Figure 8:**
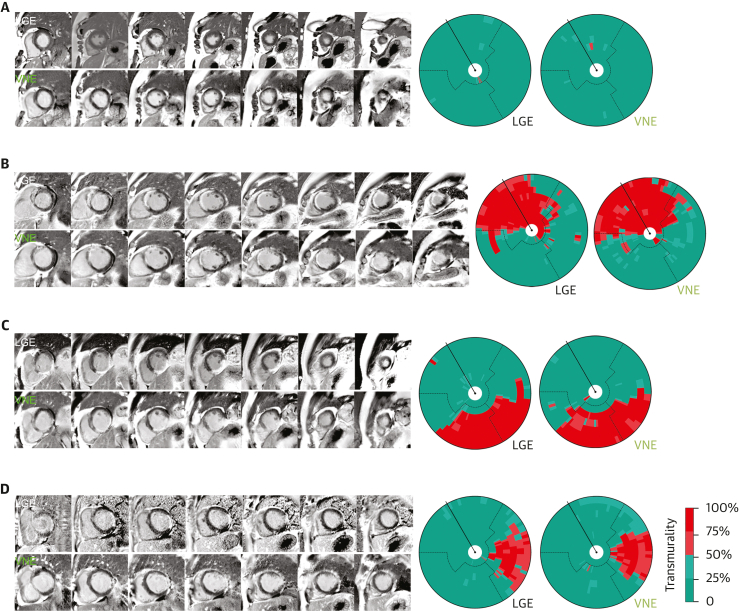
Illustrative Scans of Full Short-Axis Stack VNE for Whole-Heart Coverage Performed at Oxford (A) A case with negative LGE. (B to D) Patients with chronic myocardial infarction in the anterior territory, inferior territory, and lateral wall territory, respectively. The generated transmurality plots are presented on the right-hand side of the images. Cases from the OxAMI (Oxford Acute Myocardial Infarction) study.^29^

Although the performance was validated on 3 short-axis coverage, VNE is readily applicable to full short-axis stack coverage scans, provided that full-stack T1 mapping is acquired. Illustrative full-stack VNE scans performed at Oxford are presented in Figure 8, demonstrating its ability to detect MI in all 3 coronary territories.

## Discussion

This study represents the first prospective, blinded, multicenter clinical validation of an AI innovation in diagnostic imaging for myocardial scar visualization and evaluation, the rigorous process of which is relevant to physicians who use AI-based imaging solutions for patient care.

Although many studies have proposed AI solutions for diagnostic imaging, their clinical deployment often halts because of challenges beyond machine learning. These including difficulties in clinical implementation, reproducibility at independent sites, quality assurance, and accuracy below clinical performance metrics when subject to blinded and independent evaluation by medical experts. As a result, many potential AI solutions fail to translate clinically.

This study addressed these translational barriers through a number of concrete steps. We demonstrated that reproducible and reliable AI performance can be achieved through quality assurance of the underlying methods and scan protocols. We performed rigorous evaluation, in which VNE images were produced with no access to ground-truth LGE, images were blindly assessed by medical experts, and results were locked prior to adjudication. This minimizes bias and provides a true standard for clinical reference. We fully disclosed and dissected all discrepant cases, providing critical insights into the mechanisms and for informed clinical use. This is further enhanced by extensive quantitative analysis and independent clinical reading matching real-world clinical scenarios. The study design and findings may provide a blueprint for the safe deployment of AI solutions for medical diagnostic imaging intended for real-world patient care.

### Real-world performance of AI-derived VNE

VNE achieved diagnostic accuracy of 94.4% in detecting MI on the basis of good-quality images with clear presence or absence of MI scars. The 4 clinical readers considered VNE adequate without the need to proceed to LGE in an average of 69.7% of patients, and in these cases, VNE achieved an average diagnostic accuracy of 93.7%, comparable with that of LGE (93.9%).

The differences in infarct size of 3% with a 95% CI width of 27% are at the upper limit of observed interlaboratory inconsistencies of LGE (offsets ranging ±3%, 95% CI widths between 9% and 27%),^13^ which reflects the current clinical accuracy of LGE and is in line with our results in an earlier single-center validation.^6^ Intraobserver variability accounts for approximately one-half of intermethod variability (Supplemental Figure 2). VNE reported 6% higher transmurality than LGE in this cohort, compared with 2% higher than LGE in the Oxford internal validation data.^6^ A bias of VNE in quantifying small lesions was observed, which is attributed to the sensitivity of underpinned T1 mapping to subtle tissue changes, consistent with our previous findings.^5^ Segmental scoring is inherently more susceptible to variability from multiple sources, including reduced tissue sampling volume, minor mismatches in slice positioning, and variability in myocardial segmentation, right ventricular insertion point selection, and image artifacts, as well as in interobserver assessments, especially in lower quality images, as evident in Supplemental Figure 3 and bull’s-eye plots in Figure 7. In real-world clinical decision-making settings, the determination of myocardial transmurality and the chance of functional recovery after successful revascularization generally uses the 50% cutoff (ie, segments with <50% infarct likely has reasonable viability, whereas segments with >50% infarct likely has limited viability). In this regard, VNE-LGE agreement in this study using 3 short-axis slices was about 85%. Acquiring a full short-axis stack and long-axis images that are of good quality is necessary for VNE in clinical routine, to provide more comprehensive whole left ventricular characterization and diagnostic confidence, particularly for patients with small and/or subendocardial scars. If myocardial scar in a coronary territory cannot be definitively assessed with whole-heart VNE coverage, the decision to inject a GBCA may provide a clearer answer.

### Recommended clinical workflow

Currently, VNE may serve as a rapid triage tool for patients referred for MI scar assessment. Specifically, with regulatory approval, VNE may replace LGE if there is good image quality of both the cine and T1 map, and the VNE shows clear myocardial blood pool contrast and clear findings. As for standard LGE imaging, care should be given to certain types of MI scars on VNE, such as small subendocardial MIs in thin walls without regional wall motion abnormalities and those that can be difficult to distinguish from adjacent blood pool. For uncertain cases, operators should acquire more VNE or proceed with LGE. Our results showed that using this triage strategy, VNE can save the use of LGE in more than two-thirds of scans for MI assessment, without compromising diagnostic accuracy.

Additionally, if a patient requires LGE imaging but cannot or declines to receive a GBCA, and no other diagnostic test can answer the clinical question, a potential algorithm that may be used would be to acquire cines and T1 maps to generate VNE. As for other diagnostic imaging, good image quality is paramount; the findings based on robust images may support clinical decision-making together with other diagnostic tests and within the clinical context.

For nonischemic scar patterns, VNE has been well validated in hypertrophic cardiomyopathy, including detection of the more subtle, low- to medium-intensity patchy lesions,^5^ suggesting a feasible screening tool for this disease.

With prospective, multicenter validation now established, VNE is positioned for clinical implementation for chronic MI scar assessment, supported by an evidence-based foundation and quality control aligned with recommended frameworks for responsible AI integration in cardiovascular care.^14^ The deployment process at the 2 collaborating sites in this study, currently via one magnetic resonance scanner vendor, has validated a translational pathway for bringing this version of VNE into clinical practice right away with vendor-led regulatory approval, where the compatible modified Look-Locker inversion recovery–based T1-mapping method (shortened modified Look-Locker inversion recovery) is commonly available on its platform.

### Future development pathways

The implementation of VNE with other magnetic resonance scanner vendors would first require the deployment of a compatible T1 mapping method on the respective scanner platform. This development is currently well under way, and results will be reported in future work soon, toward generalizable and faster CMR scans for wider patient access.

The VNE concept can be generalized to other T1 mapping methods, provided that sufficient data are available for testing and validation, to assess clinical performance for those methods. Independent studies have demonstrated the feasibility of generating VNE-equivalent images using other modified Look-Locker inversion recovery T1 mapping variants,^15^ T1-weighted images,^16^ T2 imaging,^17^ or cine alone.^18^ Future work includes addressing the potential bias due to different normal T1 ranges^19^^,^^20^ and pathophysiological sensitivity^21^ among T1 mapping methods and robustness issues for less tested T1 mapping methods.

Future AI development may enable structured image quality control and fully automated, robust contouring and quantification of LGE and VNE images, which can be integrated into clinical decision-making workflows.

Future work will enhance VNE capability by generating additional dark-blood images and validating VNE in small subendocardial MI cases, comparing with the more recent dark-blood LGE.

For application of VNE to an undifferentiated, all-comers patient population, further work is under way, first handling the range of commonly encountered pathologies in patients referred for CMR, including cardiac amyloidosis, myocarditis, cardiomyopathies, and acute pathologies and then for multicenter and all-comers testing.

### Mechanistic basis of VNE for scar detection

The VNE concept relies on the observed associations of myocardial characteristics between precontrast and postcontrast images. T1 mapping has been shown to detect changes in or around areas of myocardial fibrosis when validated against LGE and histology^22^, ^23^, ^24^ and to detect areas affected by diffuse myocardial fibrosis, beyond the capabilities of LGE.^25^ Lipomatous metaplasia within chronic scars may also contribute to changes in T1 signals.^26^^,^^27^ AI and data-driven T1 mapping has also been shown to enhance the visualization of areas affected by scar on T1 maps that match those on LGE.^28^ Beyond the T1 map, the underpinning T1-weighted images and cine images may further add complementary information about image contrast and cardiac structure and motion. In VNE, generative AI models combine and enhance overt and subtle signals in precontrast images and present them in form of a virtual LGE image that closely matches that of conventional LGE.

### Study limitations

The VNE technology was trained on LGE and subsequently validated against LGE; LGE images were referenced when diagnosing MI; these factors introduce circular bias, favoring the reported LGE accuracy. Additionally, LGE is an established technology, and operators may be more used to correcting any acquisition issues more effectively than for T1 mapping. Despite involving 2 independent centers outside of Oxford, this study was an internal-external validation; after the review process, additional readers (K.E.T., A.L., O.R., M.L.) from Oxford and Fuwai but outside the VNE development team provided more independent validation (Table 5, Supplemental Appendix). For scar burden quantification on VNE and LGE, the image analyst had access to both VNE and LGE images of the same patients when placing a reference region of interest in remote myocardium for comparability (and thus not independently and blindly determined on VNE), which should be considered when interpreting the derived intermethod quantifications.

Sixty-three percent of infarct scars were anterior, compared with 20% inferior and 17% lateral. This distribution reflects the prevalence of infarct territories in patients referred for ischemia and MI assessment but may affect the generalizability of our results for inferior and lateral scar quantification.

The cohorts at the 2 sites were recruited under distinct clinical scenarios; thus, direct intersite comparison of diagnostic accuracy cannot be performed. The specificity of VNE cannot be assessed in Fuwai materials, as all patients had confirmed histories of MI (targeted for this study), and no patient had no MI on LGE. Magnevist, which was used in one of the cohorts, has now been withdrawn from the market. VNE images were produced off-line of the scanner after the scan was completed, instead of at the time of scanning. Inline implementation on a magnetic resonance console platform is under way to facilitate broader method dissemination and support clinical trials.

## Conclusions

This study represents the first prospective, multicenter, independent, and blinded validation of the VNE CMR technology. High-quality VNE may triage the need for LGE and obviate its use in more than two-thirds of referrals for chronic MI scar assessment without compromising diagnostic accuracy. These findings support that VNE that incorporates quality control holds promise to transform cardiac imaging and noninvasive myocardial scar assessment in the near future.

Perspectives **COMPETENCY IN MEDICAL KNOWLEDGE:** The AI-powered CMR technology good-quality VNE can accurately screen for and quantify myocardial scar without contrast media, in a prospective, blinded multicenter study design, in patients with suspected ischemic heart disease or history of chronic MI, compared with gold-standard LGE.

**TRANSLATIONAL OUTLOOK:** Translation of VNE at independent centers is a crucial step for ultimately offering an alternative to the current clinical standard CMR LGE that is injection free and significantly faster (10 minutes). It provides a triage tool for CMR tissue characterization and can obviate the need for LGE in patients with high-quality VNE images and clear findings. This may significantly reduce scan cost, increase clinical throughput, and expand the capabilities of CMR to include patients in whom GBCAs are contraindicated.

## Funding Support and Author Disclosures

Dr Q. Zhang has received support from the British Heart Foundation (grant FS/IBSRF/23/25190). Dr Ferreira is the Kusuma Senior Research Fellow in Cardiovascular Medicine at the University of Oxford; and has received funding from The Kusuma Trust UK. Dr Gonzales has received support for his DPhil studies from the Clarendon Fund, the Balliol College, and the Radcliffe Department of Medicine, University of Oxford. Dr Plein has received support from the National Institute for Health and Care Research Leeds Biomedical Research Centre (grant NIHR203331) and the British Heart Foundation (grant CH/16/2/32089). Dr Channon has received the British Heart Foundation Chair Award (CH/16/1/32013). Dr Vavillis is supported by grants from the Swedish Heart-Lung Foundation (grant 20230149), the Swedish Heart Association (grant 202100456), and the Swedish Society of Medicine (grant SLS-998059). Dr Atkinson is supported by the iCASE studentship, partially funded by GE Healthcare. Dr Lu is supported by the National High-Level Hospital Clinical Research Funding (No. 2025-GSP-GG-5), National Natural Science Foundation of China (grant 82471973), the Beijing Natural Science Foundation of China (grant 7242110), and the Noncommunicable Chronic Diseases-National Science and Technology Major Project (grant no. 2023ZD0504502). Dr Neubauer has received support from the Oxford National Institute for Health and Care Research Biomedical Research Centre. Dr Thomas has received support from the British Heart Foundation Clinical Research Training Fellowship (grant FD/CRT/21/24268). Dr Swoboda has received British Heart Foundation fellowship support (grant FS/CRA/22/23034). Drs Q. Zhang, Piechnik, and Ferreira have authorship rights for patent WO2021/044153 (“Enhancement of Medical Images”), patent WO202023457 (“Validation of Quantitative Magnetic Resonance Imaging”), and patent WO2020161481 (“Method and Apparatus for Quality Prediction”). Dr Piechnik has authorship rights for patent US20120078084A1 (“Systems and Methods for Shortened Look Locker Inversion Recovery (ShMOLLI) Cardiac Gated Mapping of T1”). Intellectual property is owned and managed by Oxford University Innovation. Dr Plein has research agreements with Siemens Healthineers, Philips, and Circle Cardiovascular. All other authors have reported that they have no relationships relevant to the contents of this paper to disclose.
