# Supplementary material for: Myocardial Scar Assessment Using Artificial Intelligence-Powered Contrast-Free MRI: A Prospective Multicenter Study of Virtual Native Enhancement
Source: J Am Coll Cardiol. 2026 Aug 18;88(7):717–35. doi: 10.1016/j.jacc.2026.05.029 (PMC13460244; doi:10.1016/j.jacc.2026.05.029)
Supplement: Supplementary file 1 [file mmc1.pdf]

# SUPPLEMENTAL APPENDIX

**Myocardial Scar Assessment Using Artificial Intelligence-Powered Contrast-Free MRI: Prospective Multicenter Study of Virtual Native Enhancement**

## Section I: Quality assurance of T1-mapping using phantom approach

We performed quality assurance (QA) of ShMOLLI T1-mapping sequences using our previously developed phantom approach [1], for Leeds in 2021 and for Fuwai in 2025. Full details of the phantom device, QA scan protocol, and QA approach were described in [1].

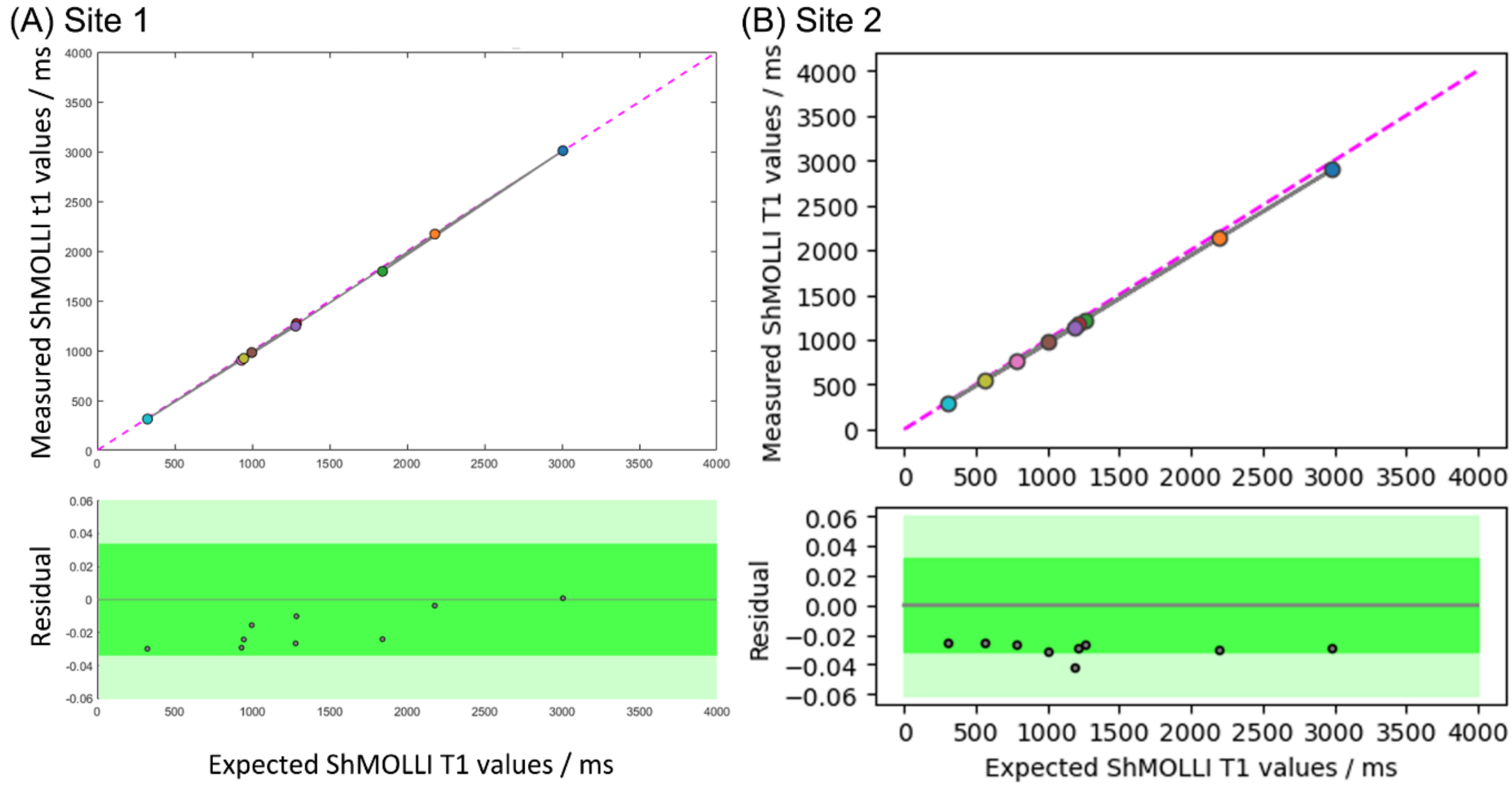


For both sites, the T1-map values were within the predefined QA range, corresponding to the 95% limits of agreement of residuals (measured T1 – expected T1)/expected T1 derived from the n=1004 phantom scans at Oxford [1].

Both sites show perceivable systematic shift of residual values toward the lower boundary of the QA range. This appears consistent with changes to acquisition methods introduced by Siemens between software version VB17 and VE11 (unpublished, a separate paper pending). Given that the pathological T1 deviations in MI are vastly above the observed drift, the VNE-AI model produced VNE images that were comparable between the two sites and with Oxford.

**References:**


[1] Zhang, Q., Werys, K., Popescu, I. A., Biasiolli, L., Ntusi, N. A., Desai, M., ... & Piechnik, S. K. (2021). Quality assurance of quantitative cardiac T1-mapping in multicenter clinical trials–A T1 phantom program from the hypertrophic cardiomyopathy registry (HCMR) study. International Journal of Cardiology, 330, 251-258.

## Section II: Full list of low-confidence 'possible infarct' cases and image characteristics (n=29)

We disclose and categorize below the full list of cases in which VNE was scored as low-confidence 'possible infarct'. The original comments from the clinical reader are provided alongside each case. Main reasons were (A) poor image quality of the underpinned cine or T1-maps, (B) slice mismatch between cine and T1-maps, (C) VNE subtle signals and poor quality, (D) non-ischemic heart disease. Operators should therefore be advised to pay attention to these factors when applying VNE in clinical practice.

| Reason A: Poor image quality of the underpinned cine or T1-maps (n=20) | Original comments by the operator |
|---|---|
| 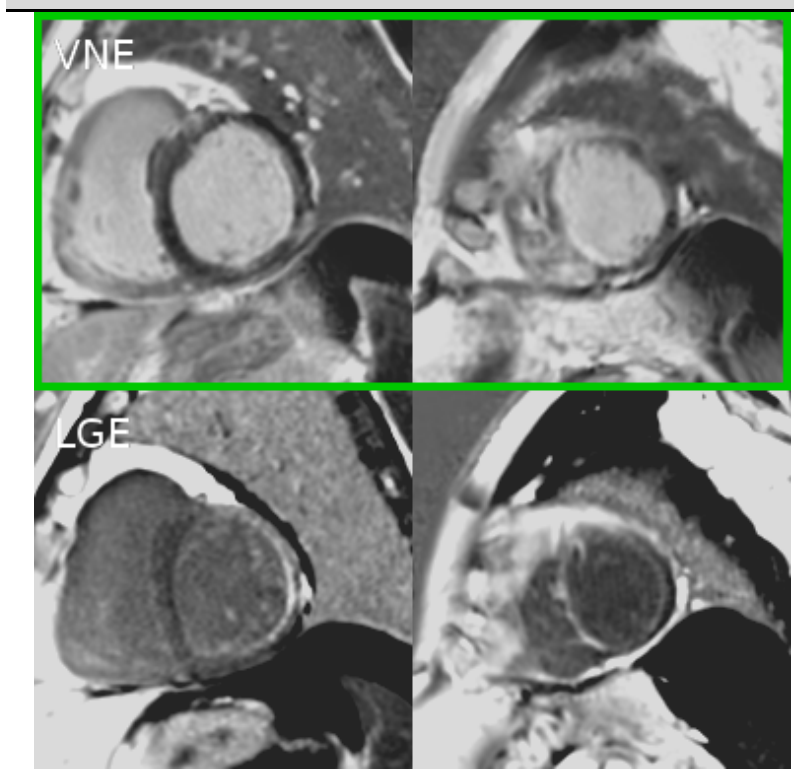 | "Possible infarct on VNE; Abnormal VNE; possible infarct but not sure; won't rely on VNE for decision; anterior poor R2 (quality) of T1-map." |
| 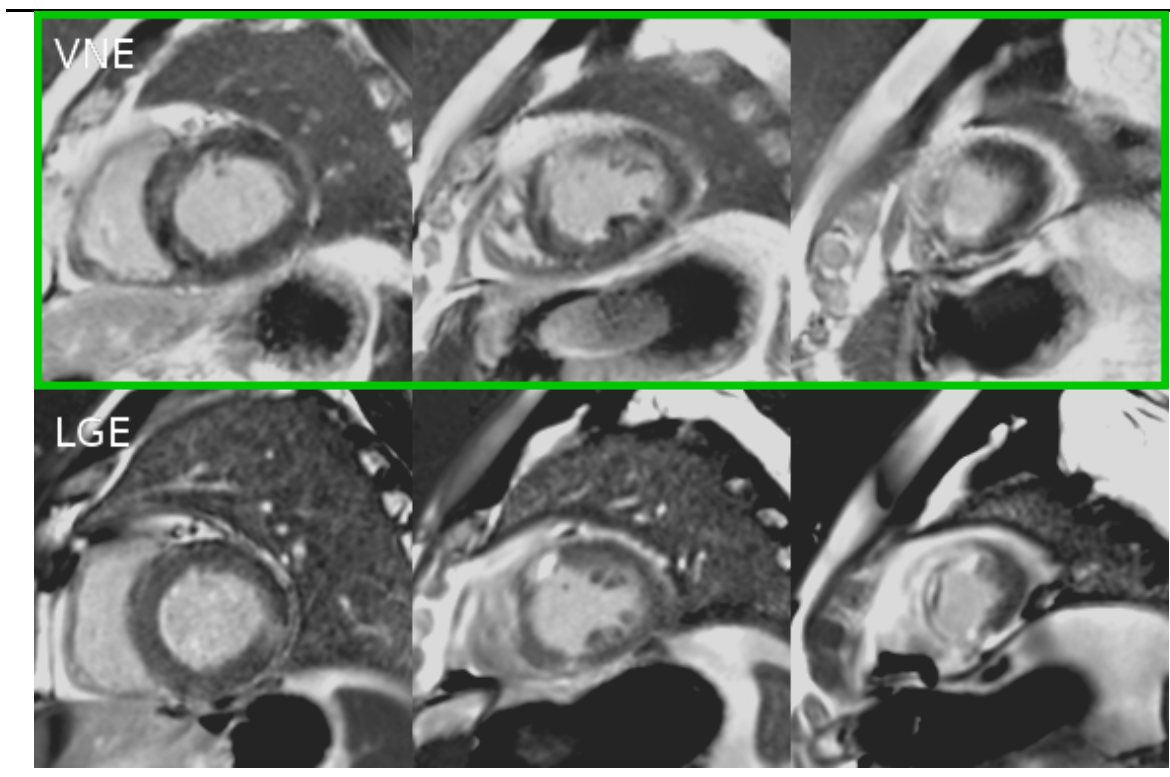 | "Possible infarct on VNE; T1-map minor breathing motion; VNE septal mid-wall lesions could be LVOT; T1 artefacts in anterior RV-LV junction; VNE showed enhancement but affected by T1-map fat off-resonance artefact. VNE quality questionable, possible infarct." |
| 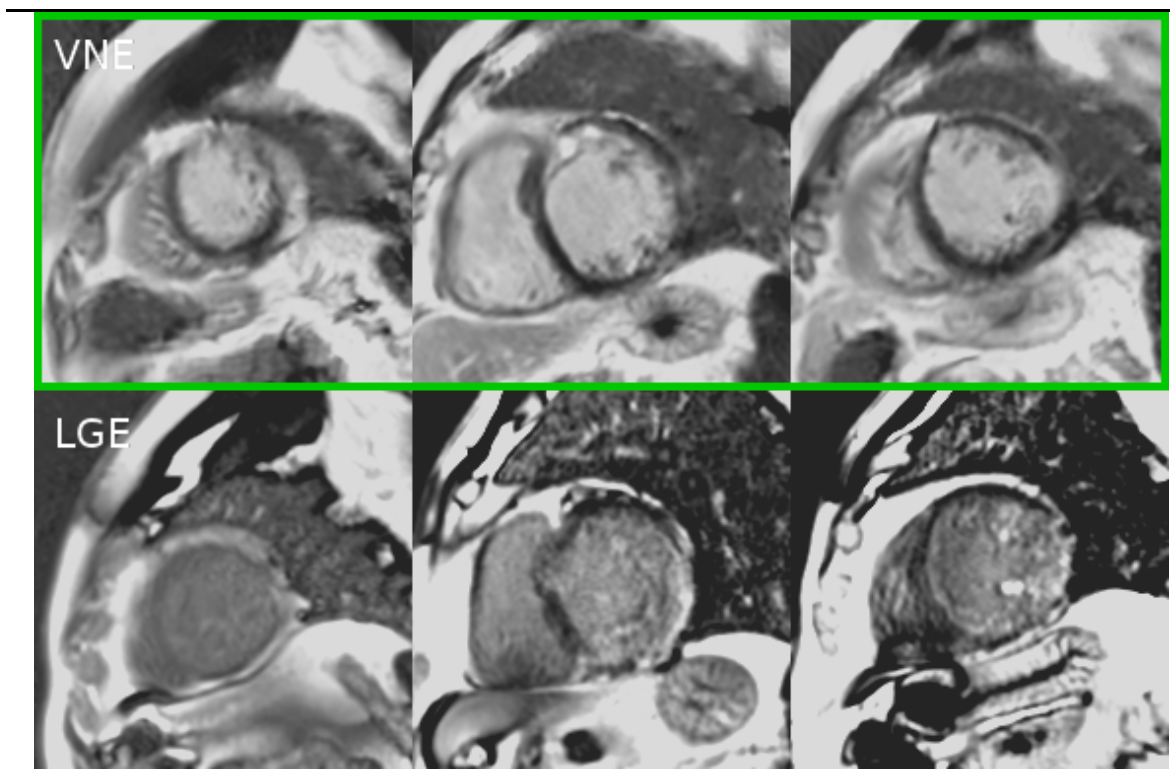 | "Possible infarct on VNE; cine motion; T1-map breathing motion artefacts." |

| | |
|---|---|
| 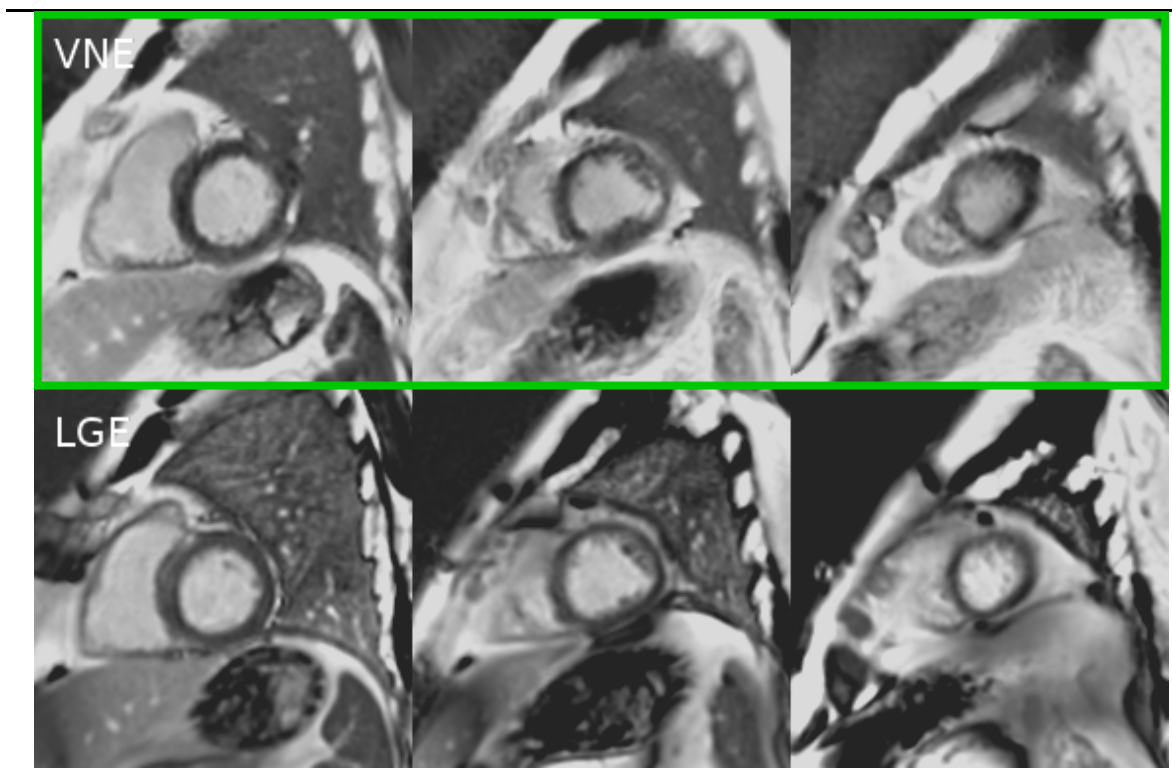 | “Possible infarct on VNE; motion artefacts on T1-maps; mid-slice VNE possible infarct on inferolateral wall, thinned wall, near apical slice; query blood pool.”jh26 |
| 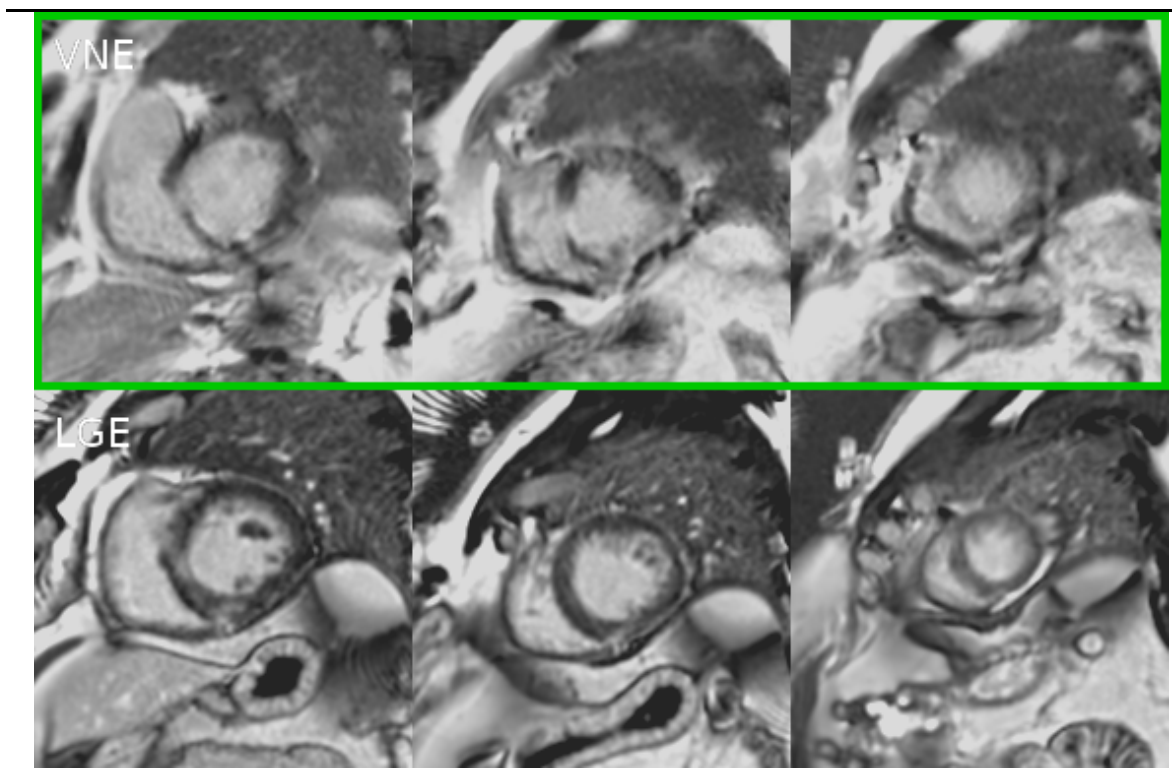 | “Possible infarct on VNE; cine poor quality, T1-map severe motion; VNE image quality questionable.” |
| 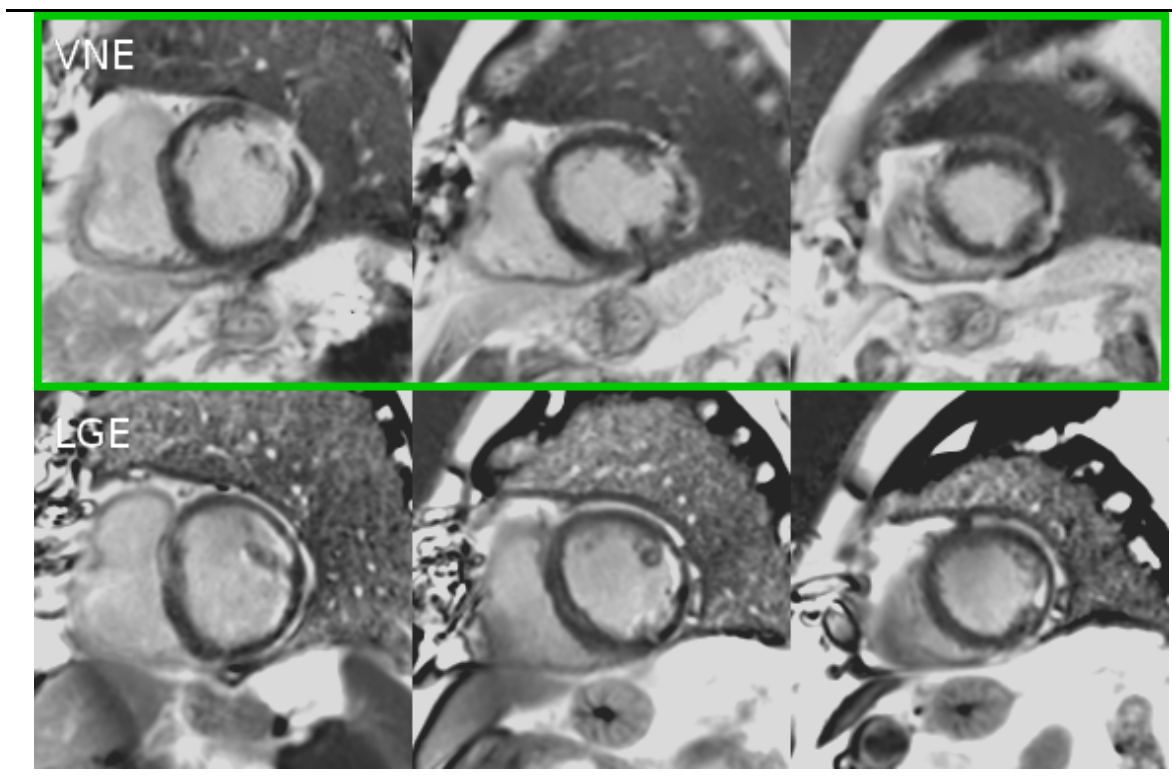 | “Possible infarct on VNE; T1-map breathing artefact and wire artefacts; T1-map poor quality due to multi-slice acquisition (protocol error); T1-map fat off-resonance artefact.” |
| 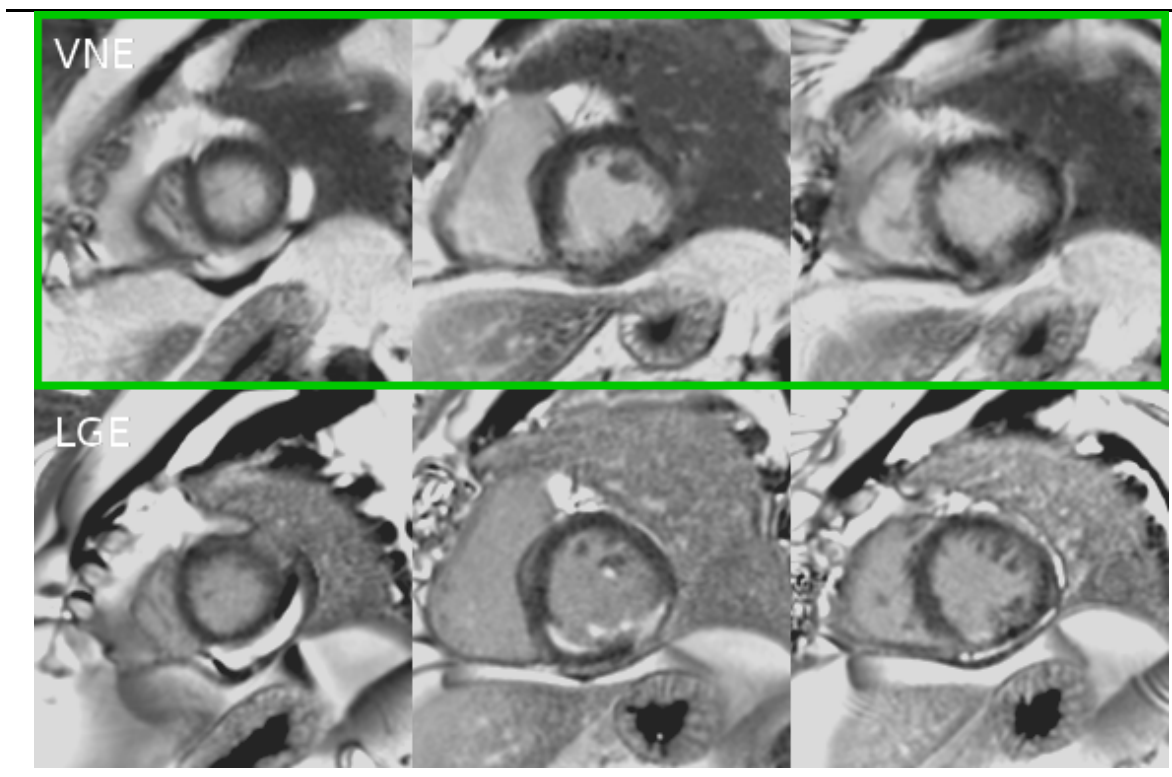 | “Possible infarct on VNE; T1-map mis-triggering artefacts.” |

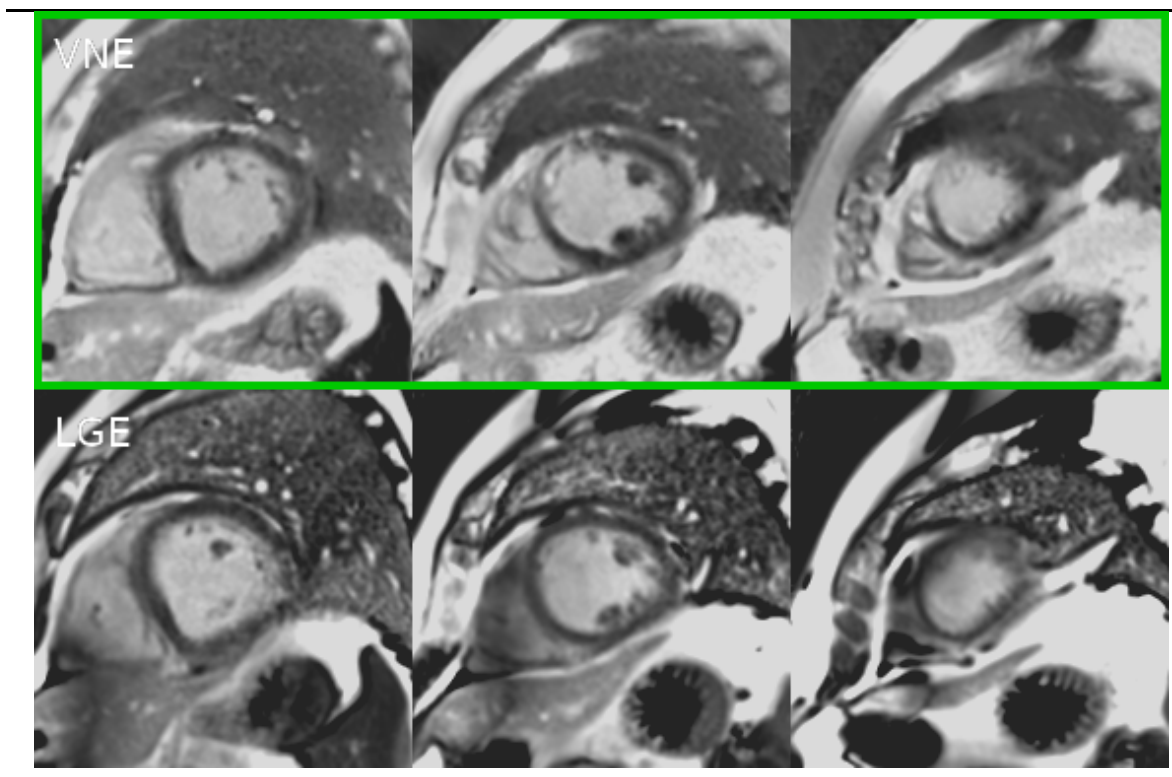


“Possible infarct on VNE; dyssynchronous contraction on cine images; biphasic septal motion.”

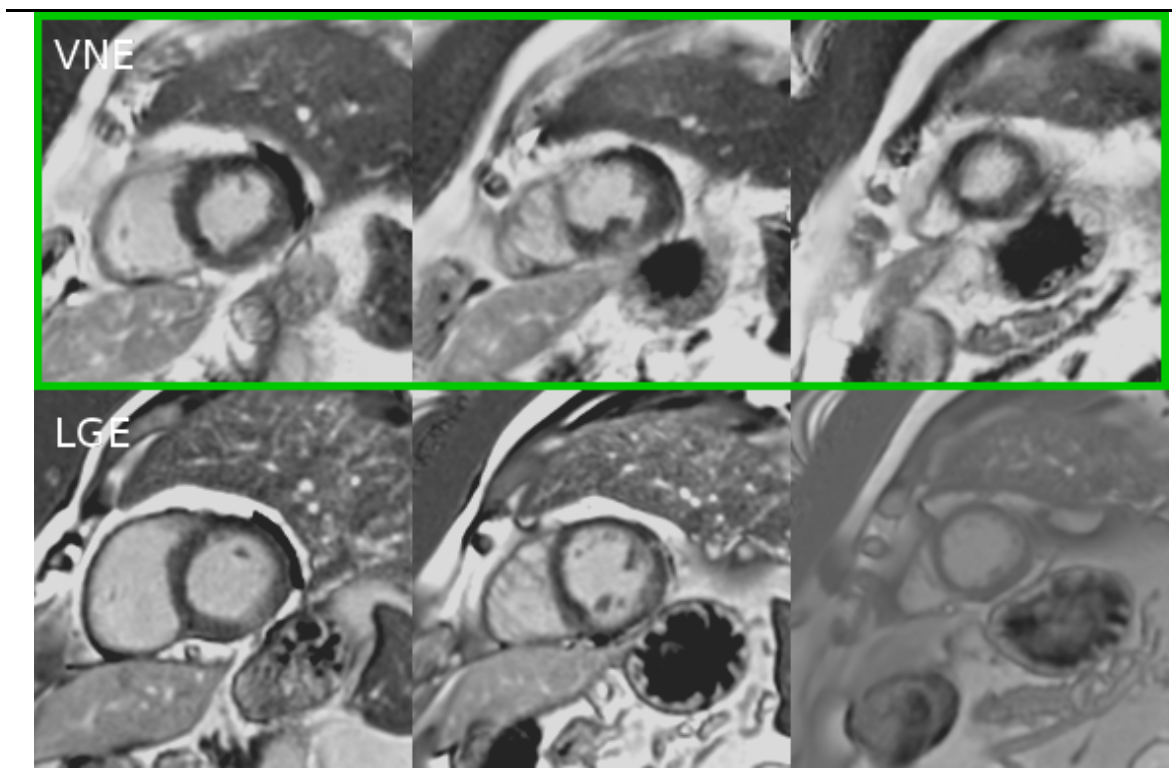


“Possible infarct on VNE; Blurry cine images; query mistriggered beats on cine; T1-map breathing artefacts.”

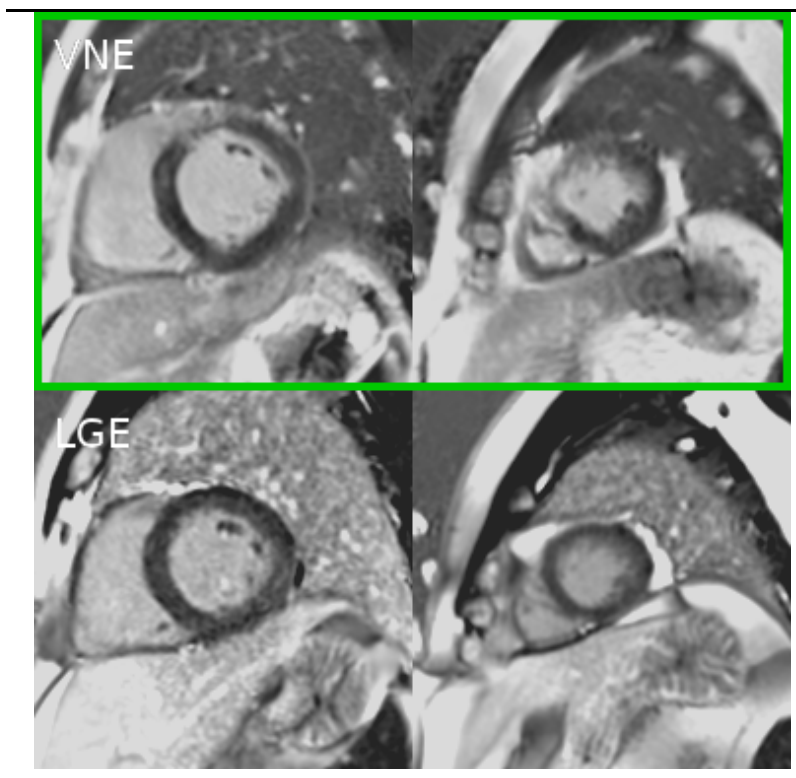


“Possible infarct on VNE; possible sub-endocardial VNE signals; T1-map impaired in septal wall; VNE images blurry in anterior wall and anterior septum.”

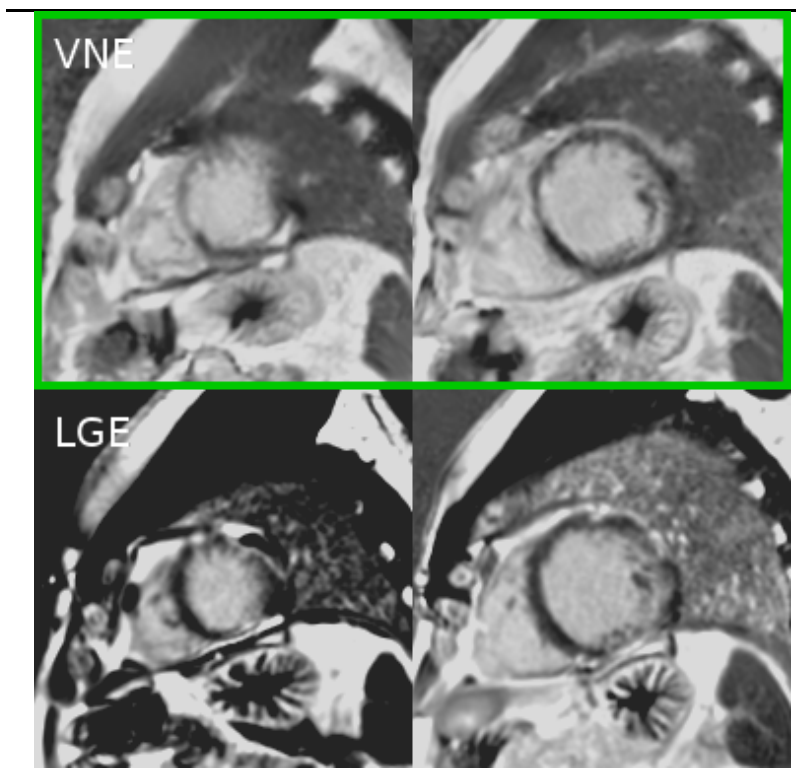


“Possible infarct on VNE; cine through plane partial volume, very apical slice; cine blurry, query mistriggered beats.”

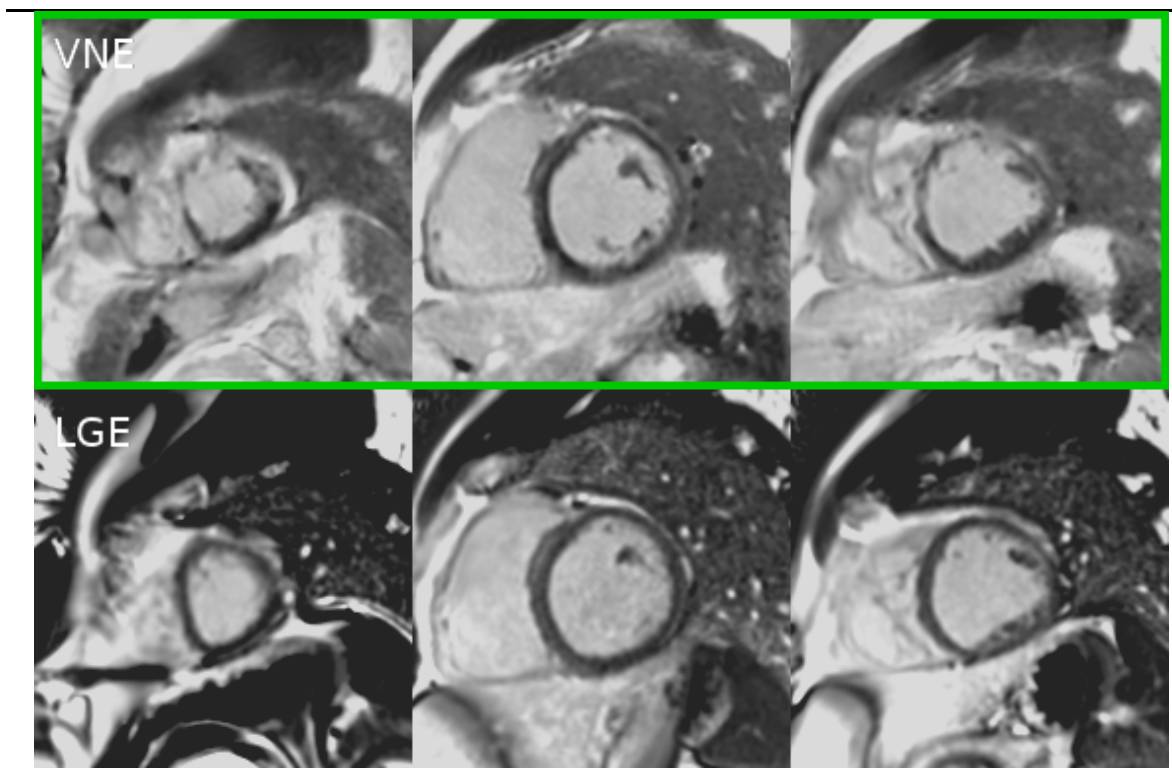


"Possible infarct on VNE; T1-map fat off-resonance artefacts."

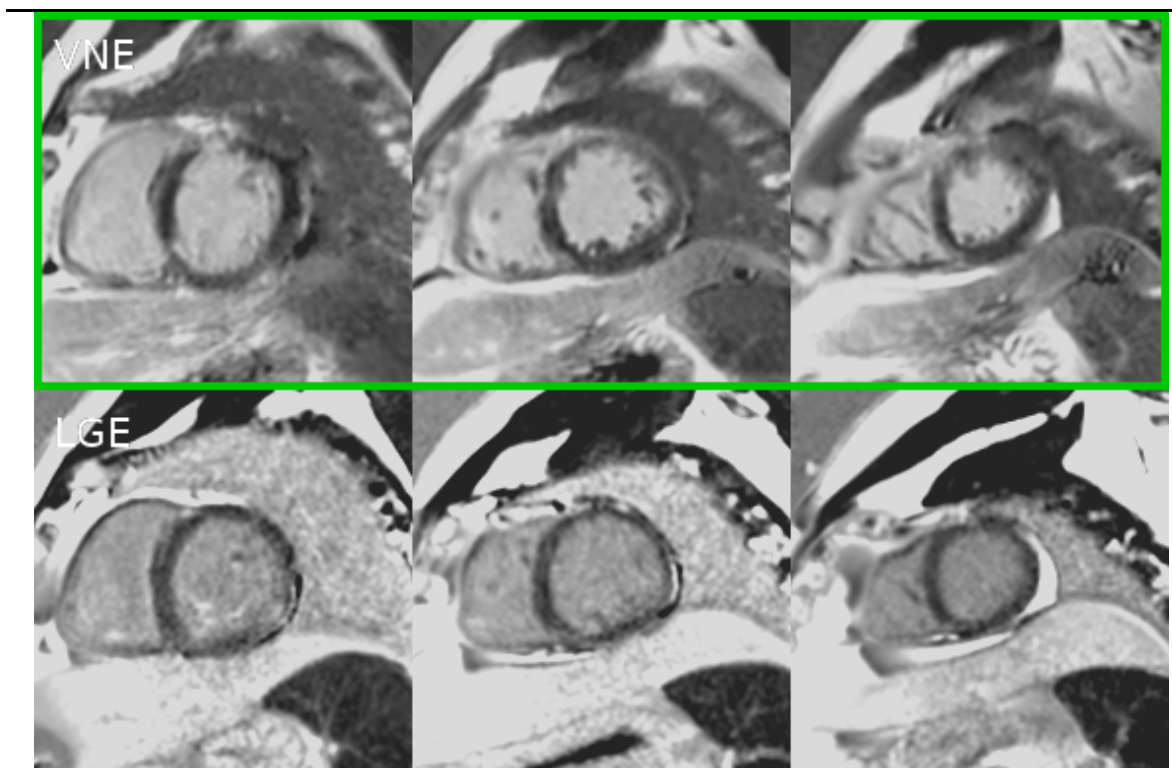


"Possible infarct on VNE; T1-map artefacts affecting the anterior septum; cine motion artefacts; VNE impaired quality, possible infarct in anterior and inferior wall, but likely due to cine artefacts."

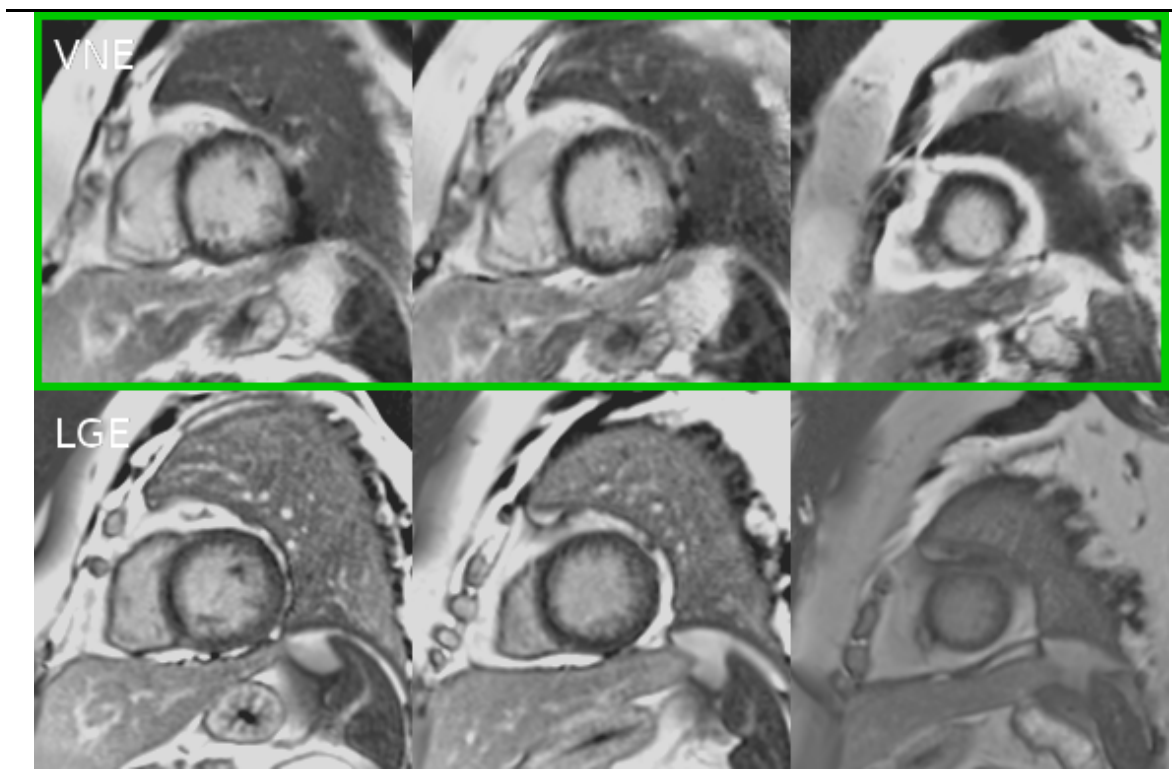


"Possible infarct on VNE; severe T1-map motion; severe cine motion artefacts."

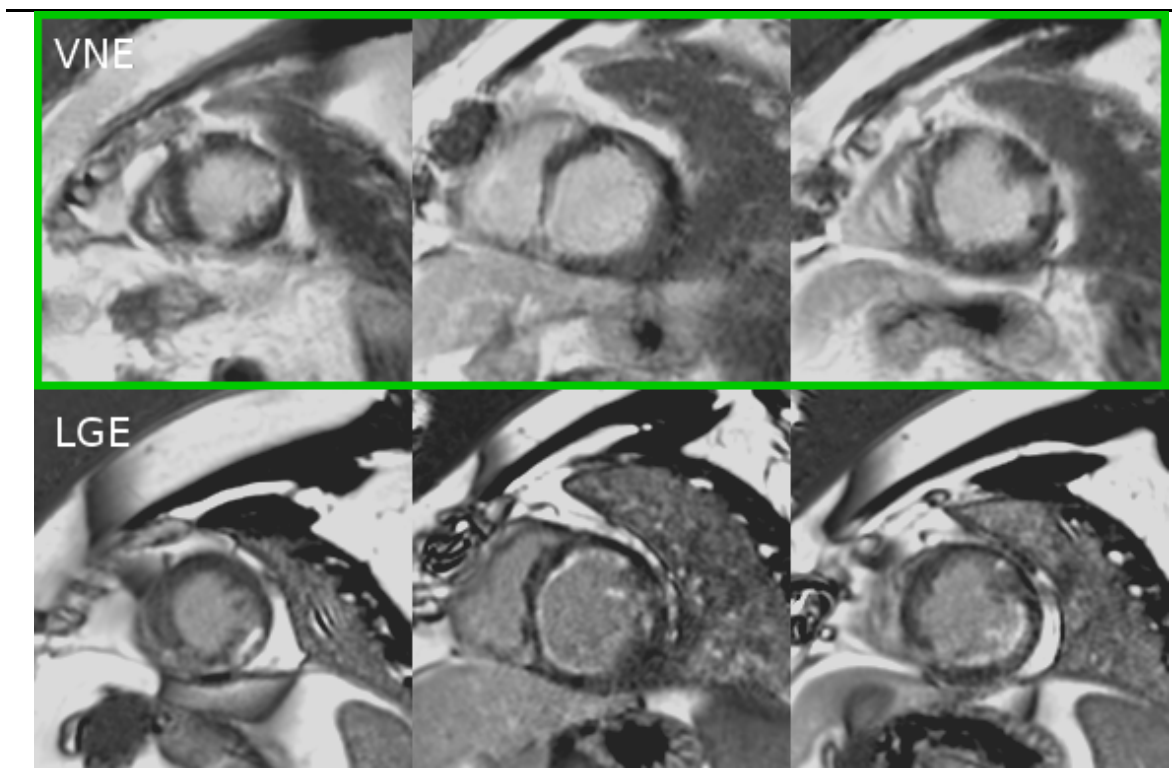


"Possible infarct on VNE; cine severe motion artefacts; T1-map breathing motion artefacts."

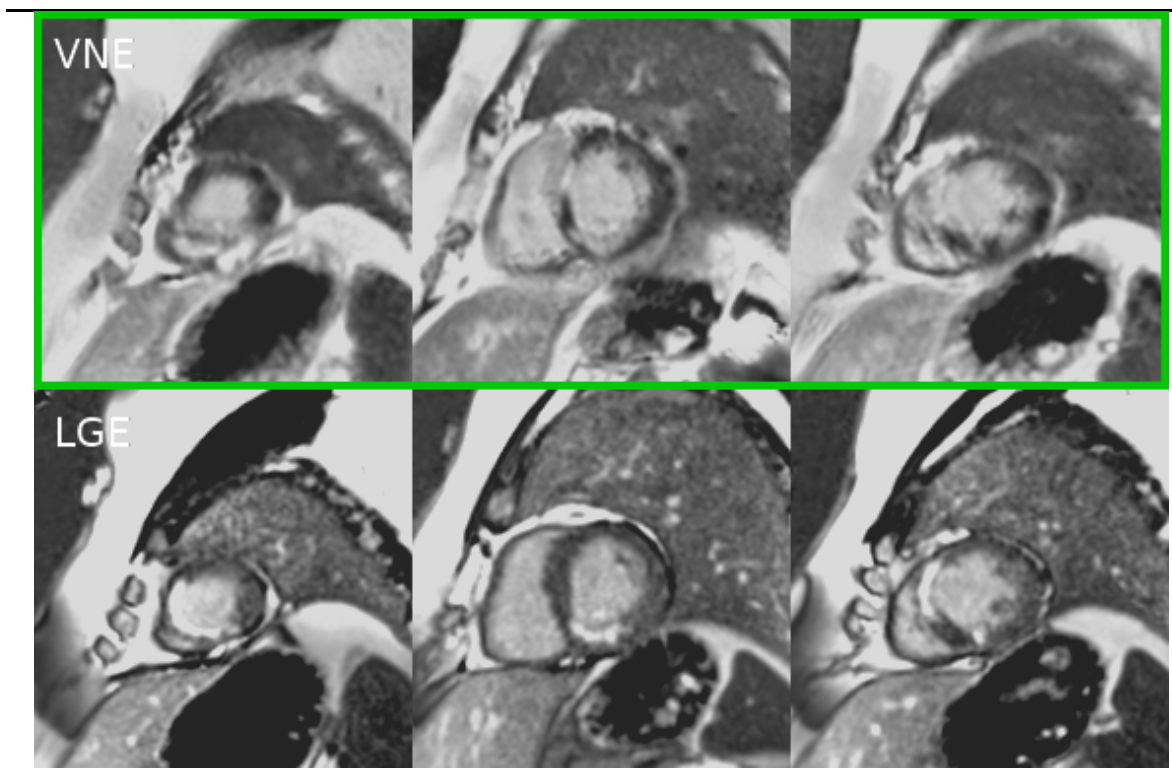


"Possible infarct on VNE; T1-map severe breathing motion artefact, cine severe artefacts."

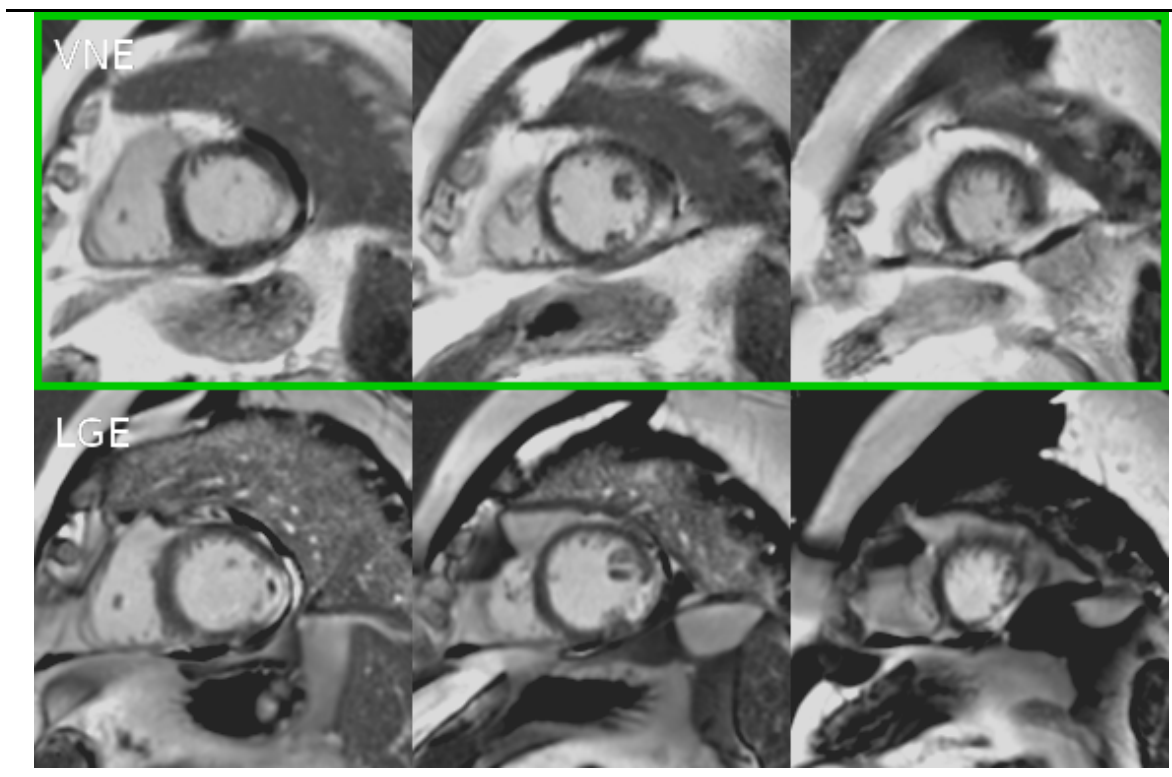


"Possible infarct on VNE; T1-map fat off-resonance artefact"

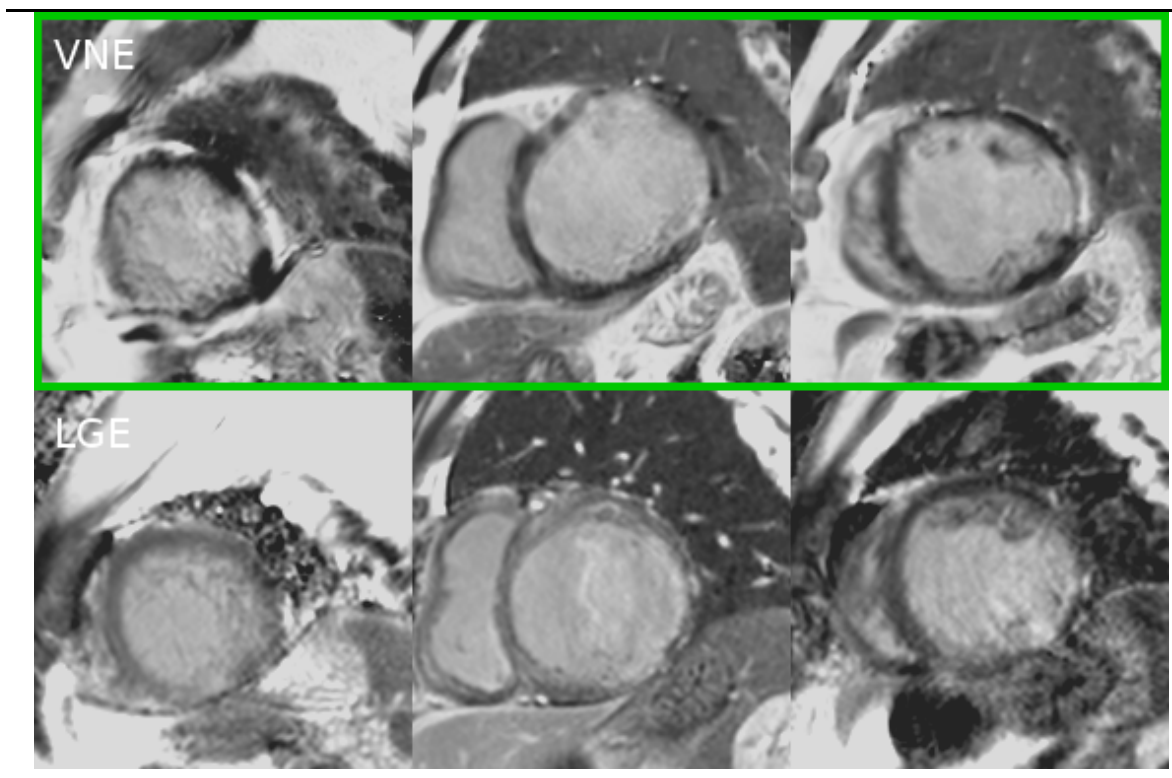


"Possible infarct on VNE; Cine mistriggered beats; R2 of T1-maps is grey (poor quality); black line artefacts in anterior wall; possible inferolateral infarct."

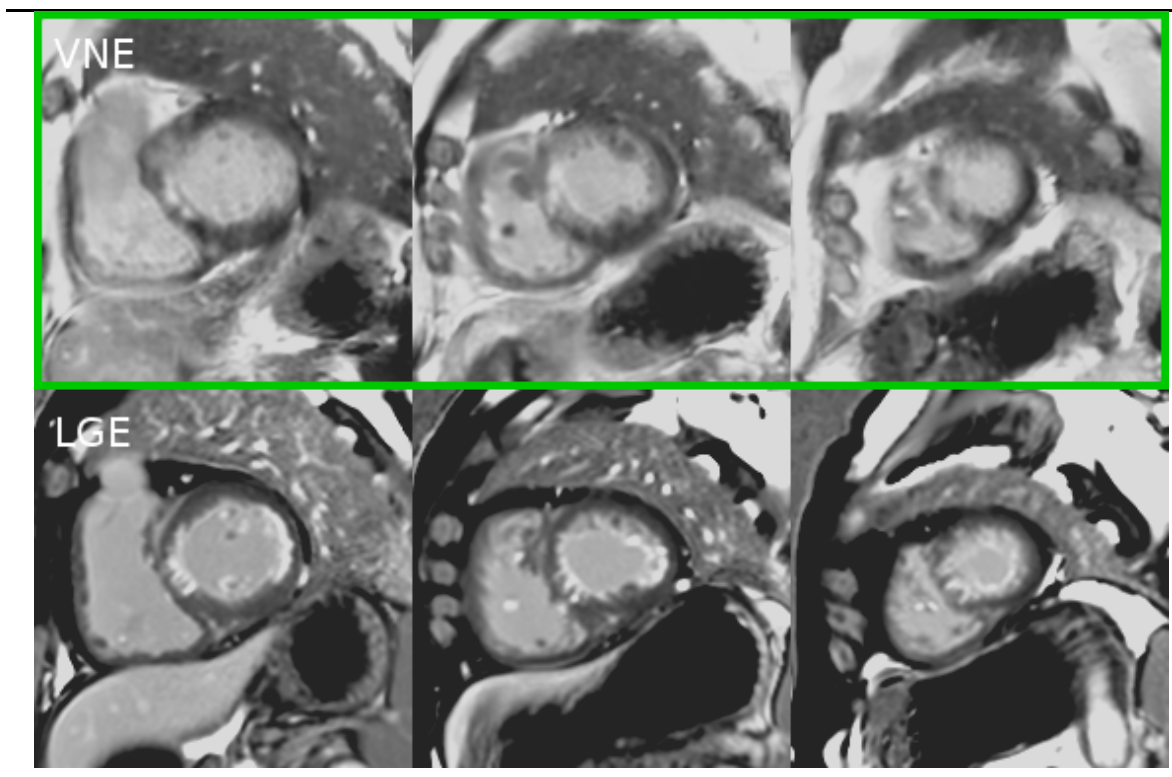


"Possible infarct on VNE; wrapping artefact on cine; T1-map motion artefacts and poor quality; patchy VNE enhancement in septum, possible subendocardial infarct in septum."

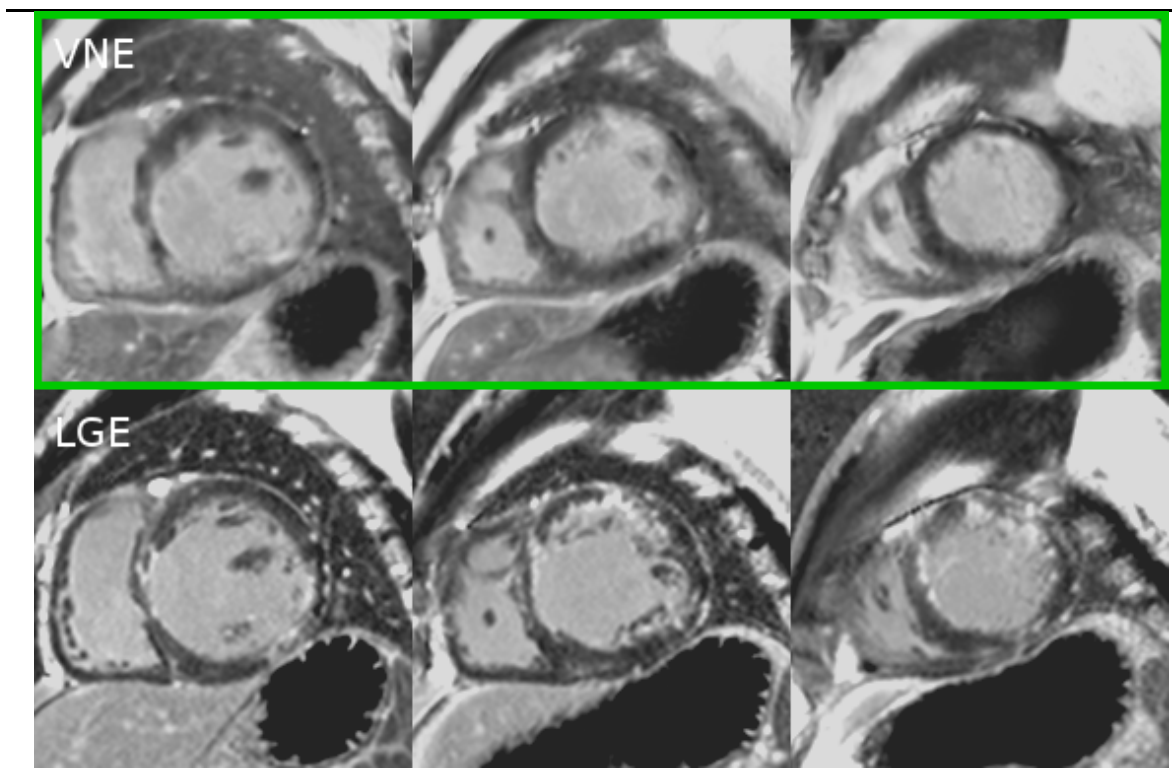


"Possible infarct on VNE; R2 (quality-control map) of T1-maps not reconstructed; possible infarct in septum, anterolateral and inferior walls."

Reason B: slice mismatch between cine and T1-map (n=1)

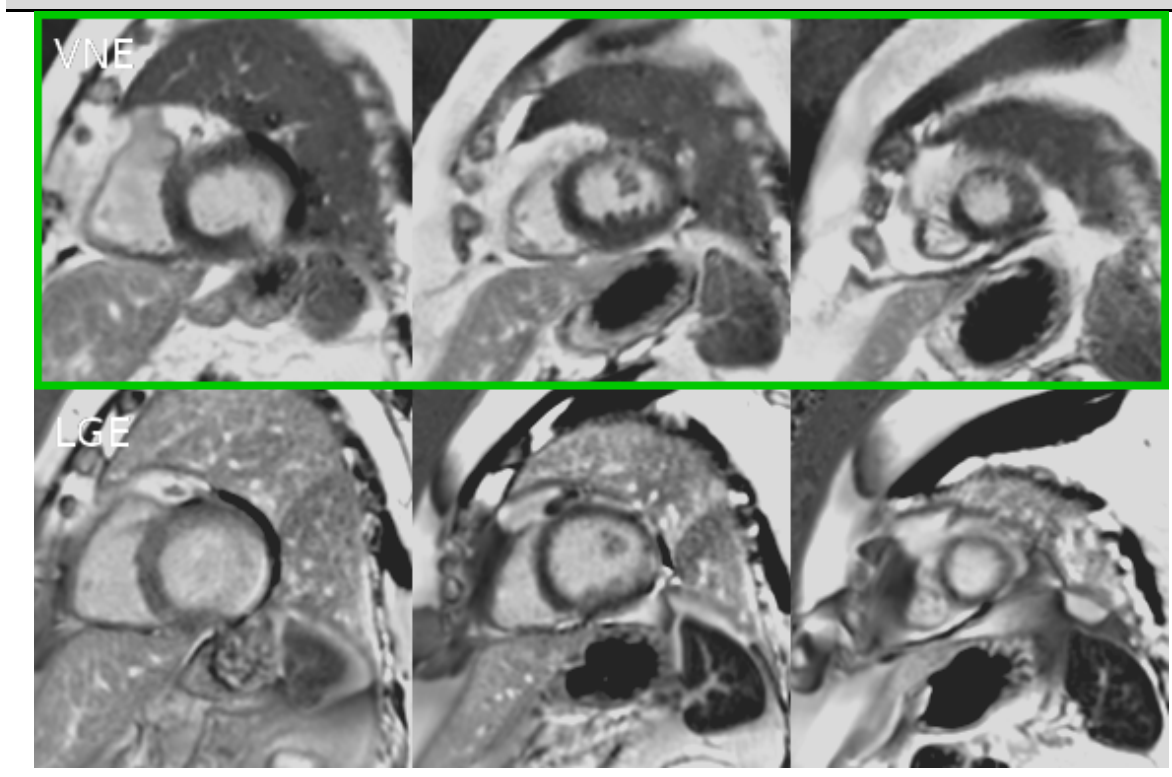


"Possible infarct on VNE; cine mismatch, cine thinned out, cleft or old scar; VNE blurry and possible infarct; Epical anterior wall may be T1-map artefact."

Reason C: VNE subtle signals and poor image quality (n=7)

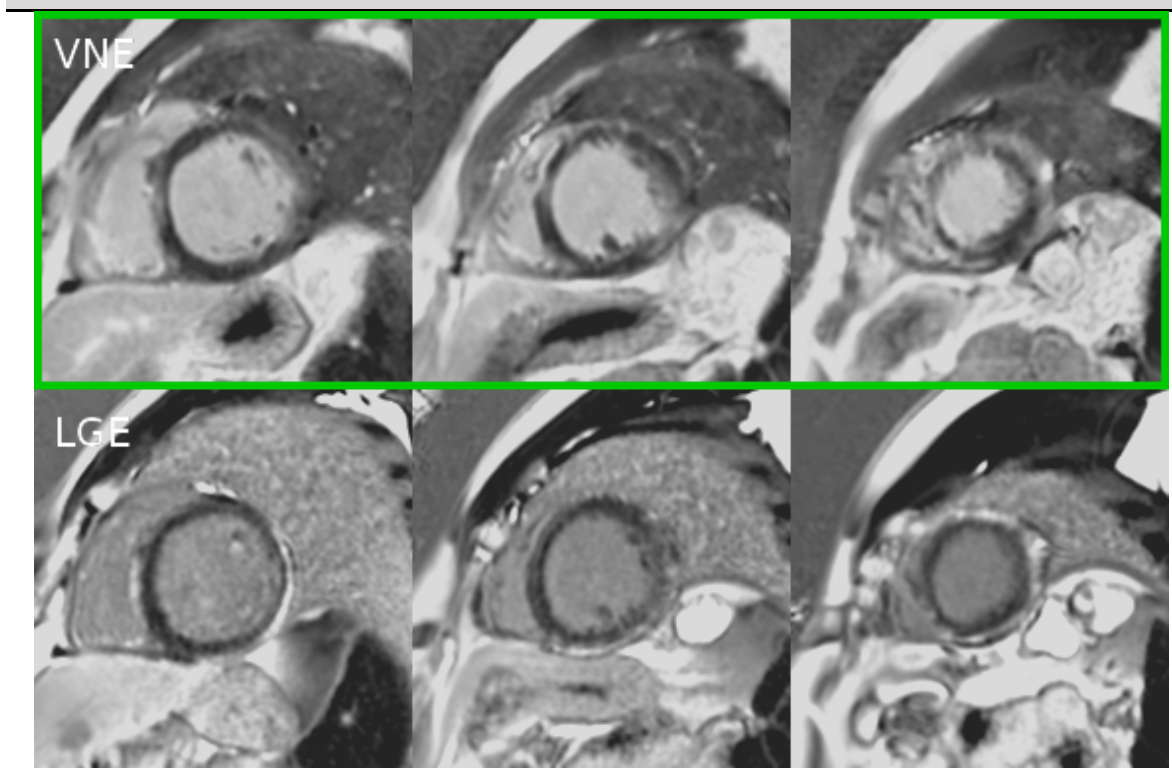


"Possible infarct on VNE; VNE subtle enhancement at septum and inferolateral wall; cannot be sure."

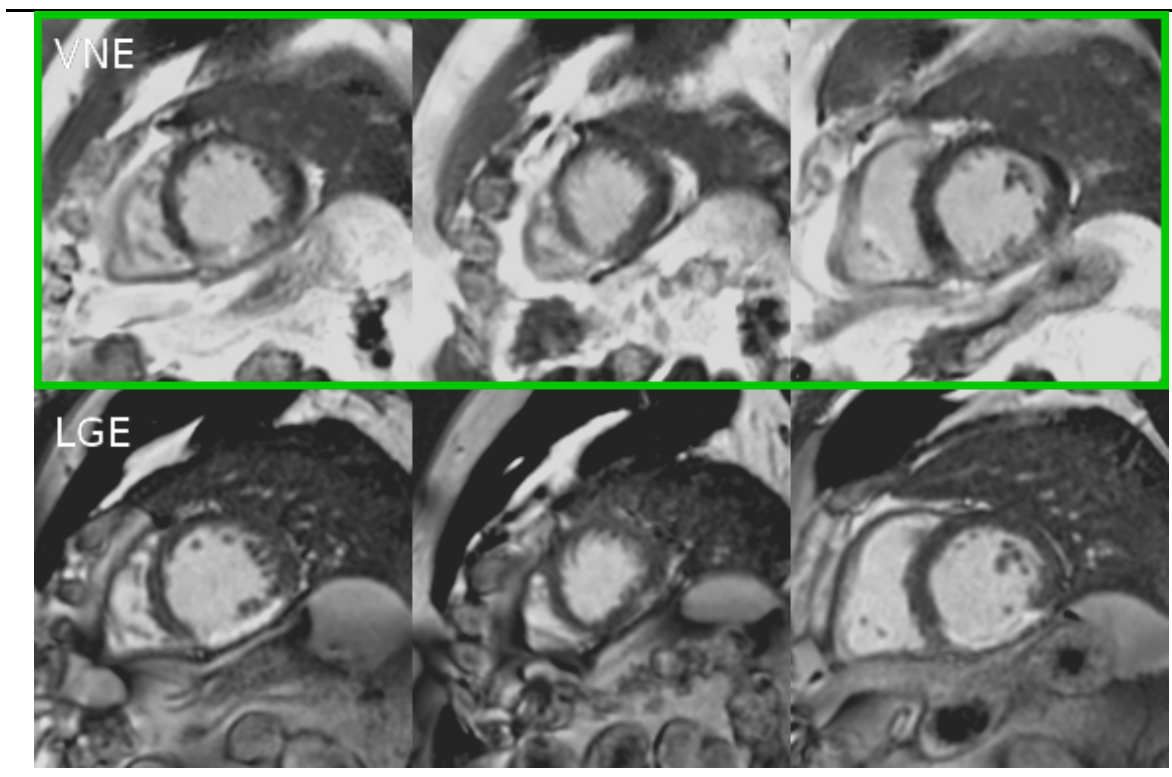


"Possible infarct on VNE; VNE poor image quality and possible inferior scar; T1-map mistriggered beats."

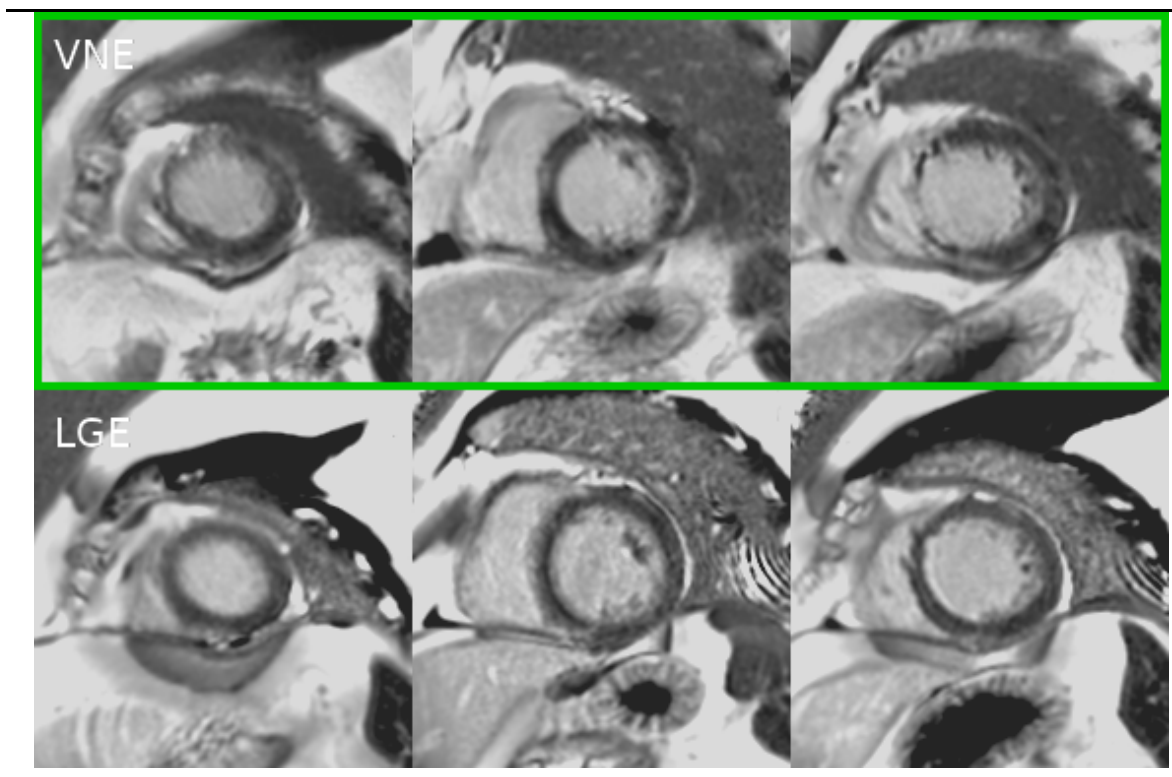


"Possible infarct on VNE; cine motion artefacts; VNE infarct on septum; VNE possible infarct on inferolateral wall; query blood pool; definition is blurred and probably no an infarct."

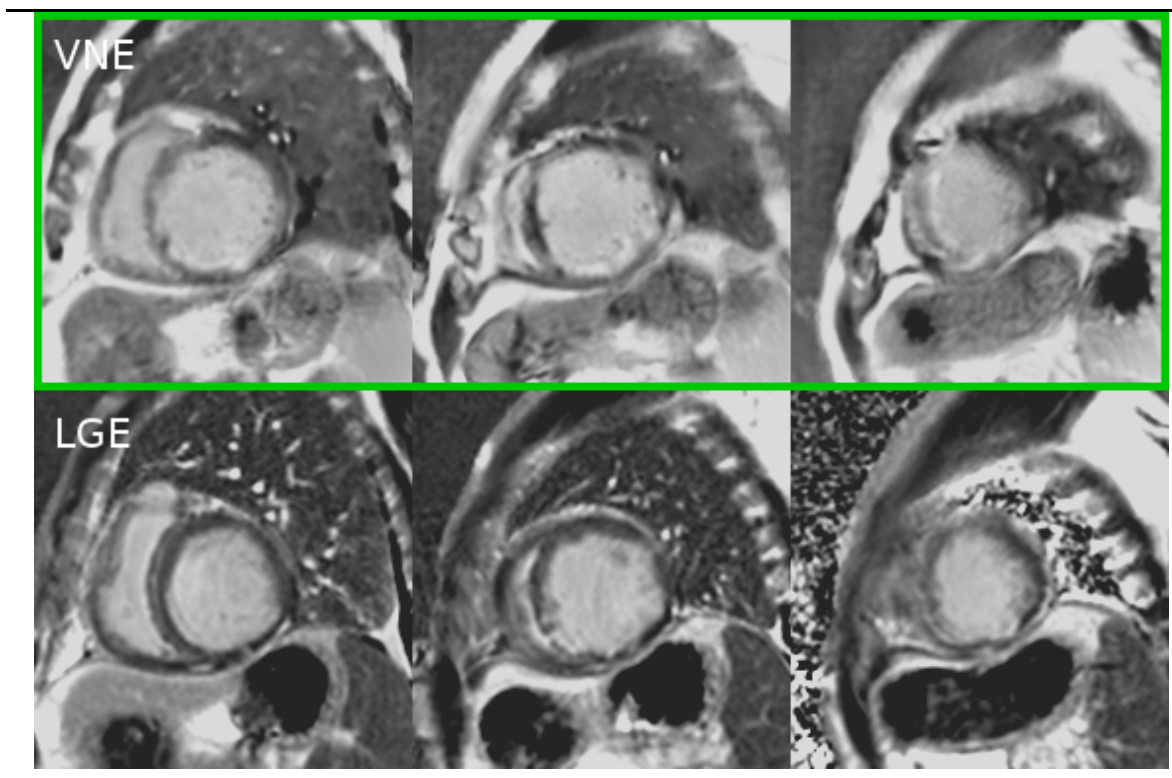


"Possible infarct on VNE; inferior and inferoseptal walls; VNE images blurry."

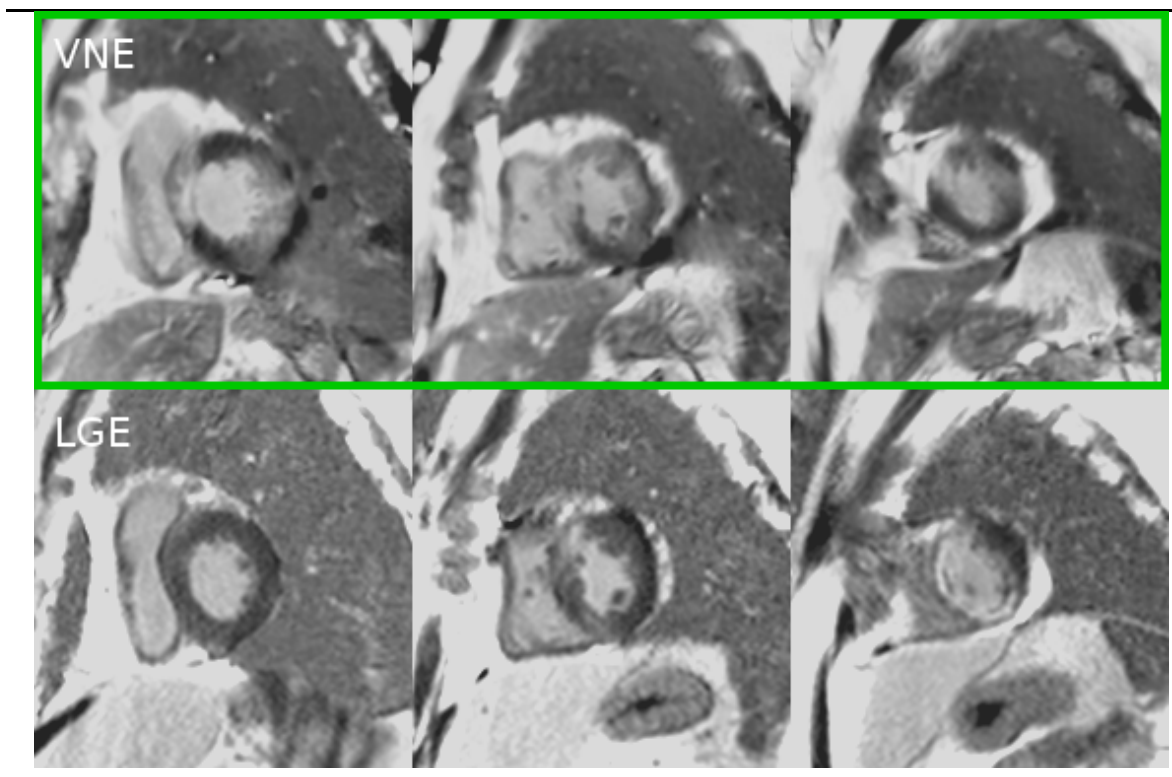


"Possible infarct on VNE; VNE enhancement in septum, possible LVOT; inferior wall artefact on T1-maps; artefacts on cine; signals in septum but not diagnostic; basal slice VNE is blurry; mid and apical slices reject."

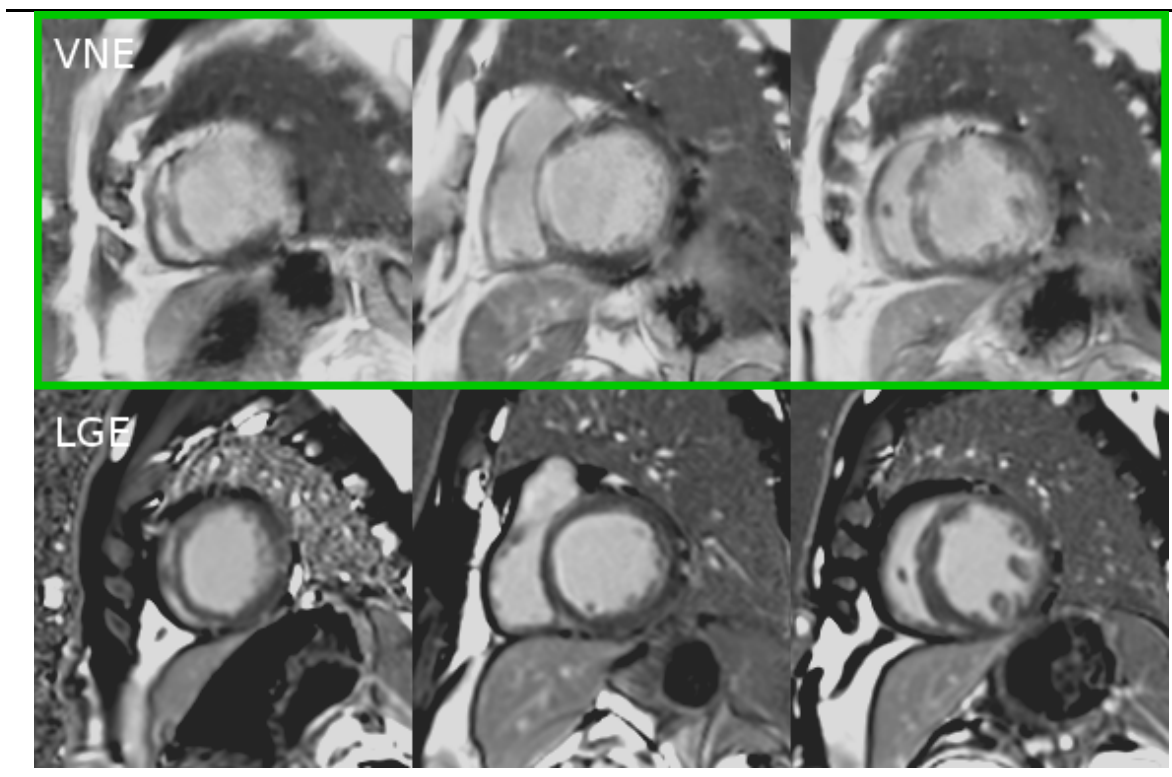


“Possible infarct on VNE; anterior wall, septum possible infarct.”

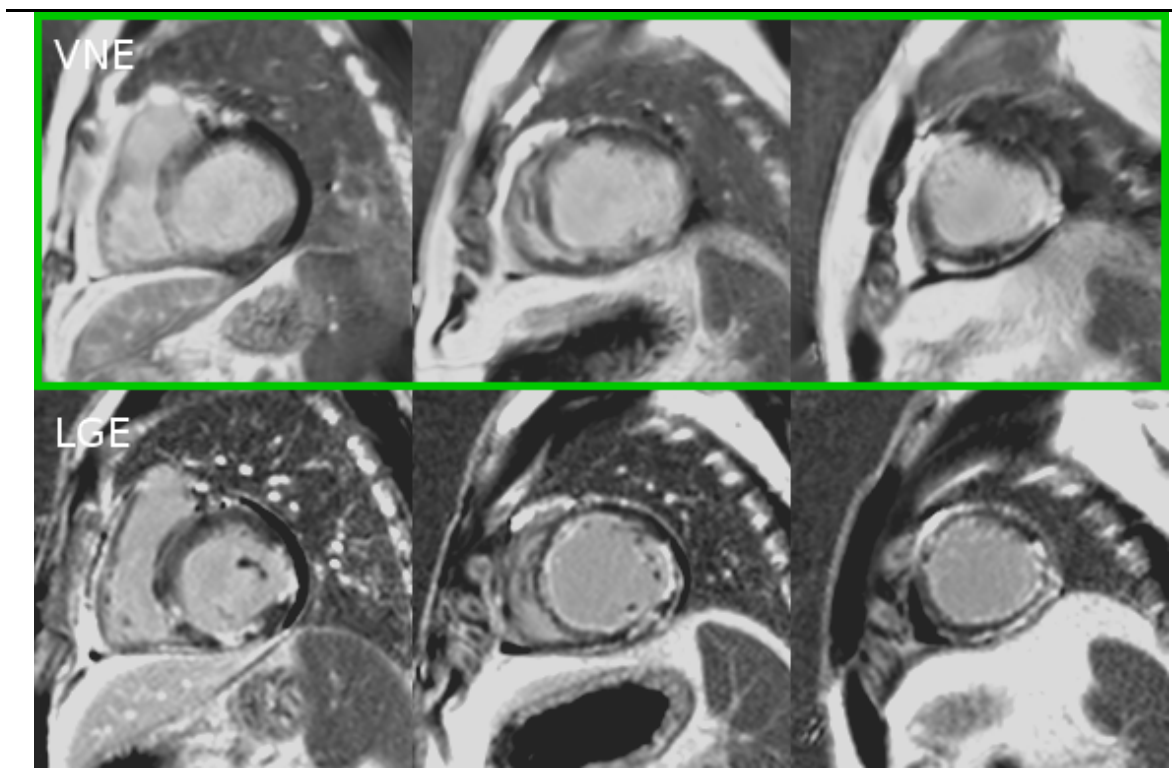


“Possible infarct on VNE; R2 (quality control map) of T1-map not reconstructed; T1-map fat off-resonance artefacts; Vortex artefact in inferior septum; T1-map artefacts in inferolateral wall.”

Reason D: Non-ischemic heart disease (n=1)

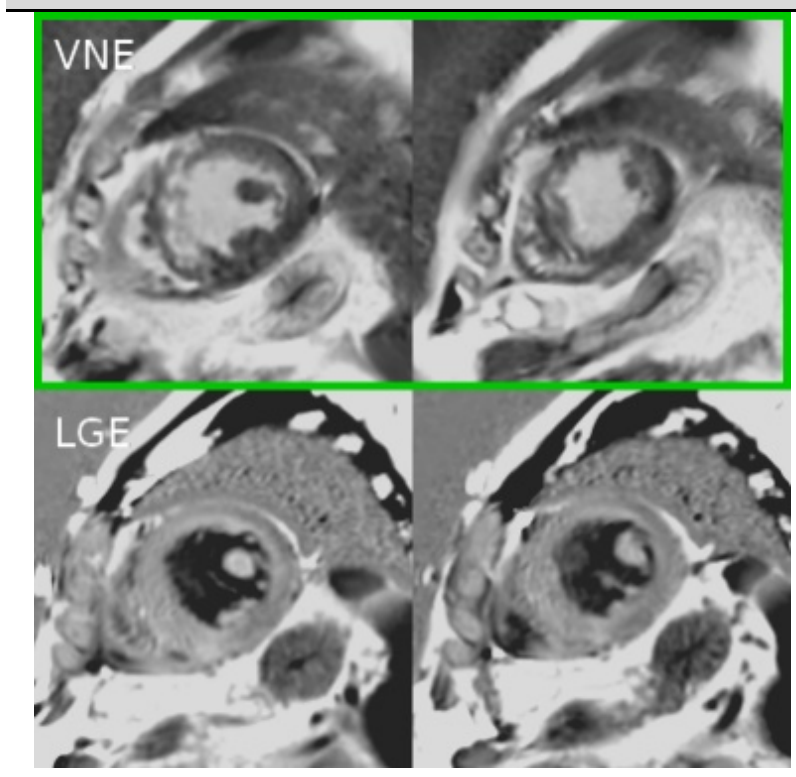


“Possible infarct and non-ischemic scar on VNE; T1-map impaired at septum by off-resonance artefacts; VNE non-ischemic patches in septum, appearing very heterogeneous; query HCM.”

## Section III: Selected examples of high-confidence VNE cases

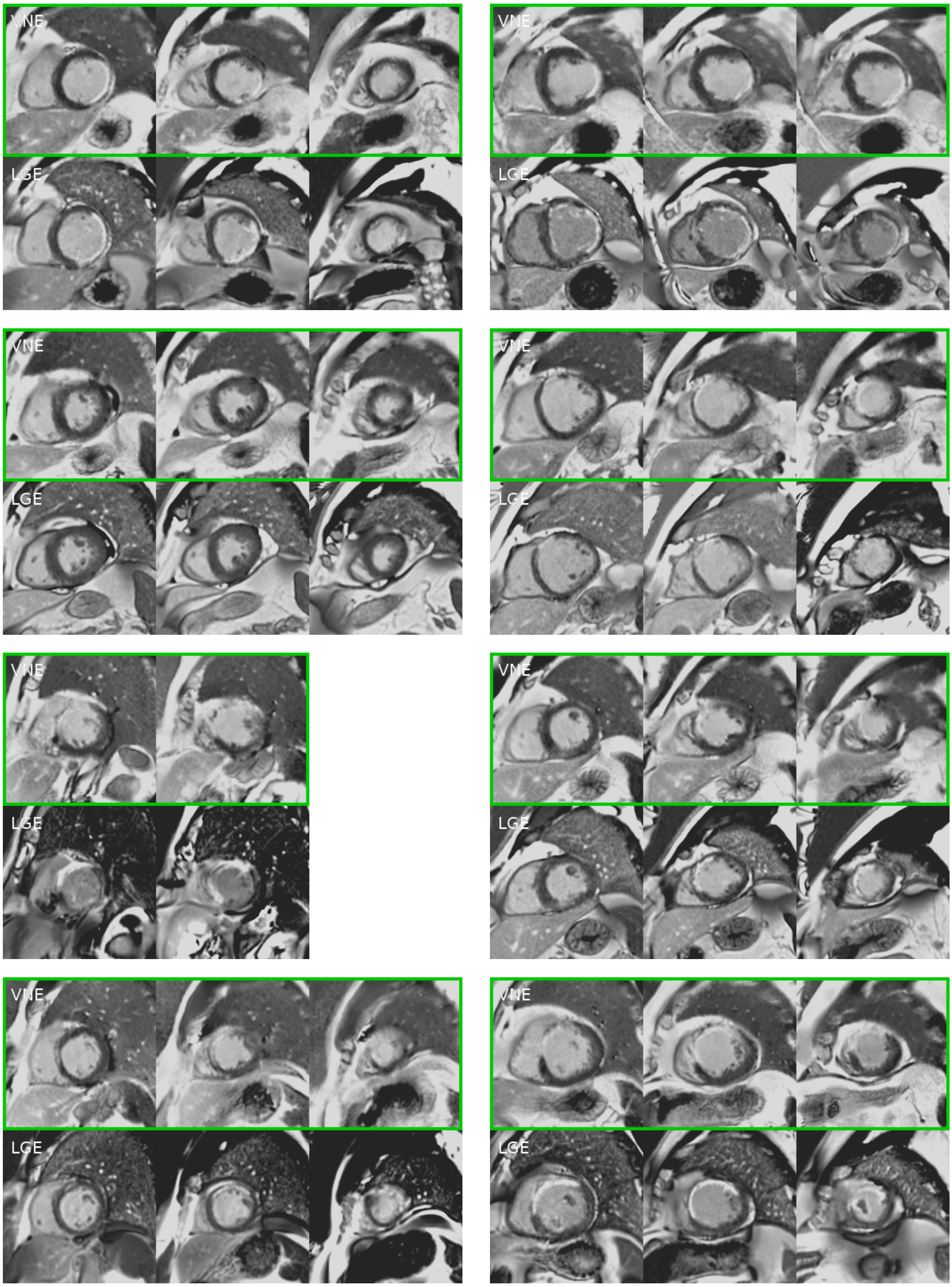

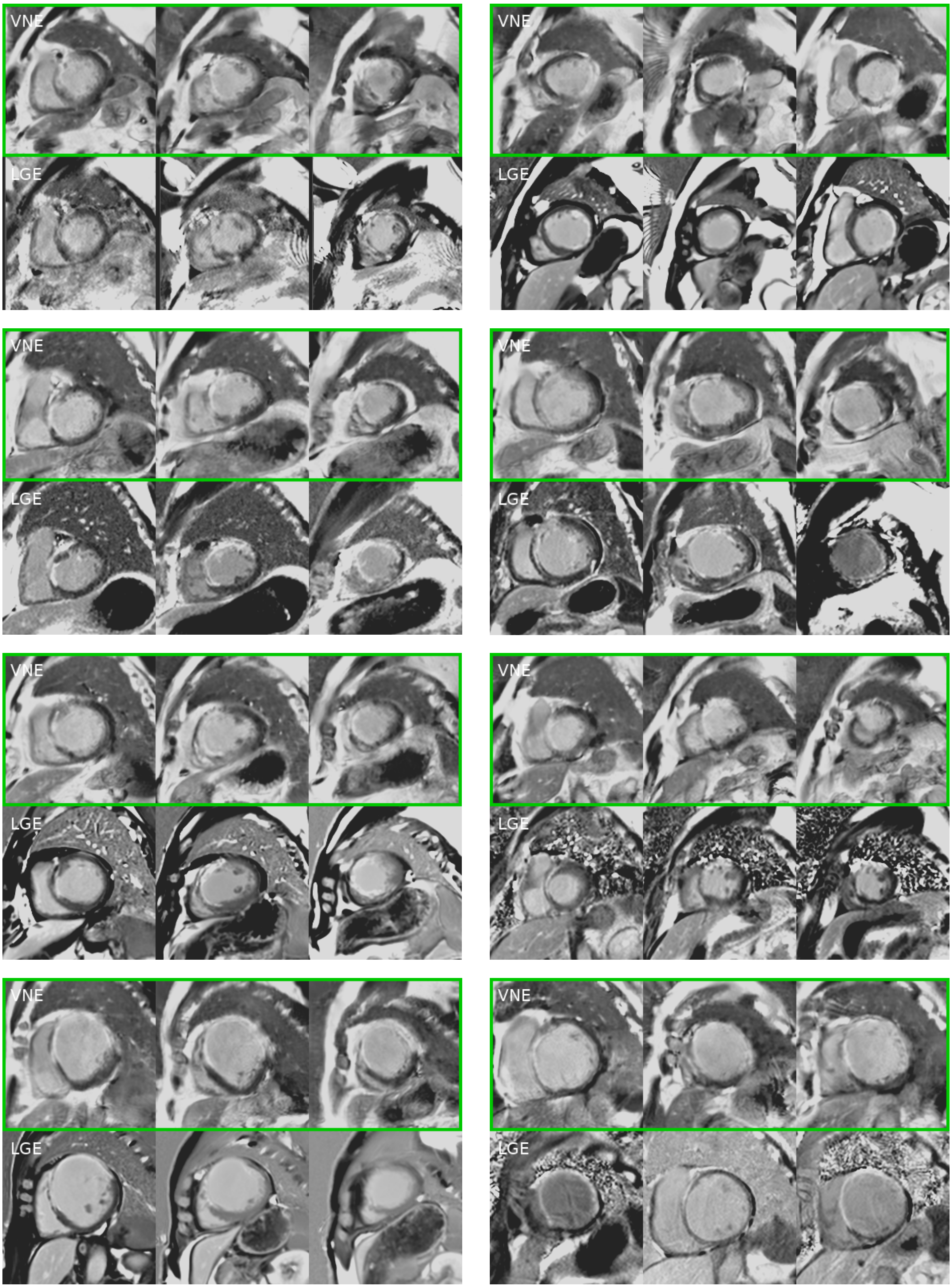
VNE
LGE
VNE
LGE
VNE
LGE
VNE
LGE
VNE
LGE
VNE
LGE
VNE
LGE
VNE
LGE

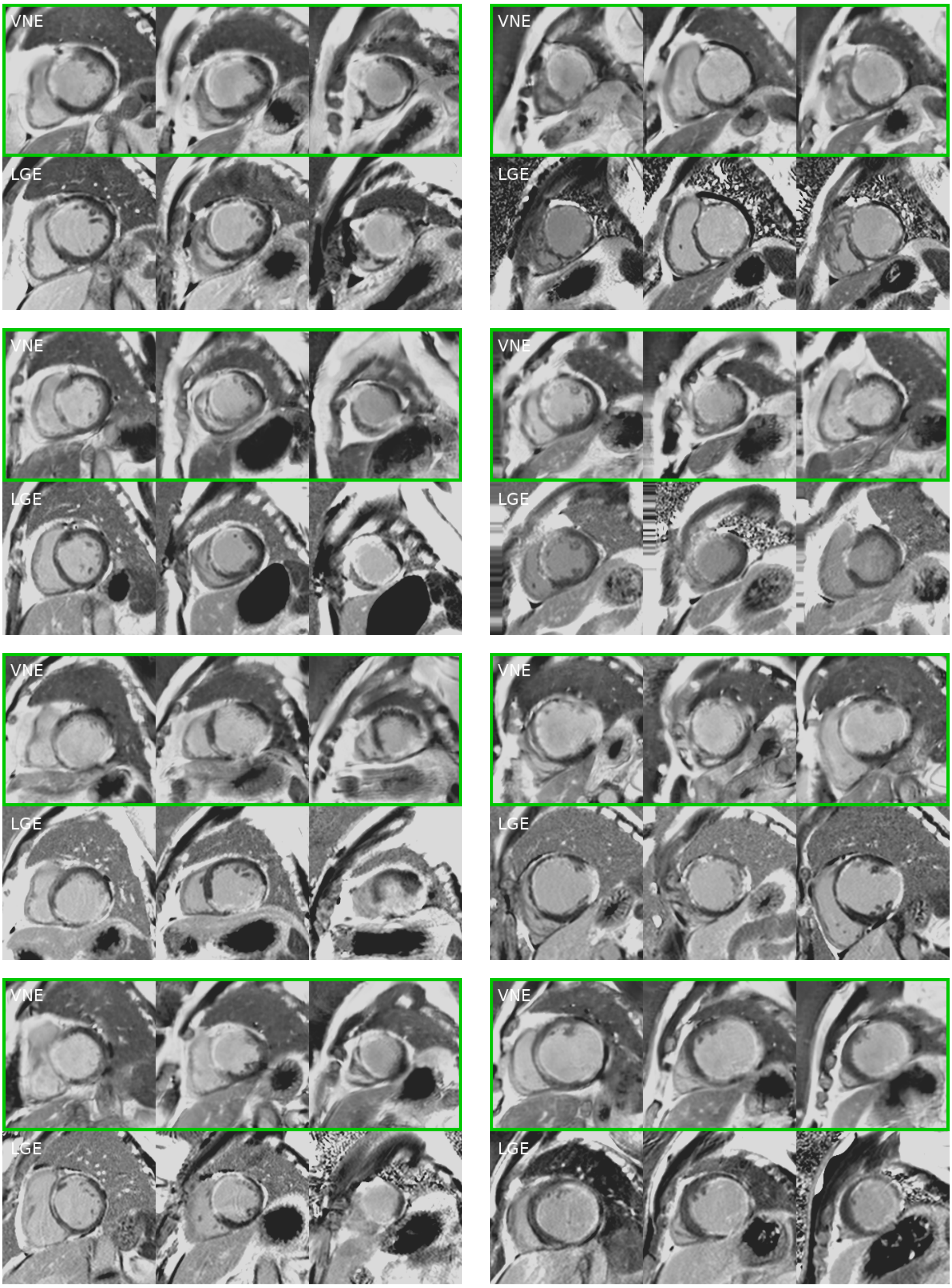
VNE
VNE
LGE
LGE
VNE
VNE
LGE
LGE
VNE
VNE
LGE
LGE
VNE
VNE
LGE
LGE

## Section IV: Matched-slice multi-reader blinded analysis of diagnostic accuracy of VNE compared to LGE

To assess the impact of limited VNE slice coverage, a matched-slice analysis was performed on VNE vs LGE, by scoring the presence of infarct and transmurality of scars based on LGE and LGE slices at matched location, typically at 3 short-axis slice positions per patient.

We developed two dedicated User Interfaces (UI) for multi-observer experiments of (A) diagnostic accuracy and (B) transmurality assessment of VNE and LGE using 3 short-axis slices per patient, as shown in the Figure below. VNE or LGE images were shuffled and presented randomly, always accompanied by the T1-map and cine video. Readers did not know whether each case was VNE or LGE. Four readers (K.T., A.L., O.R., M.L.) independently scored the full set of images.

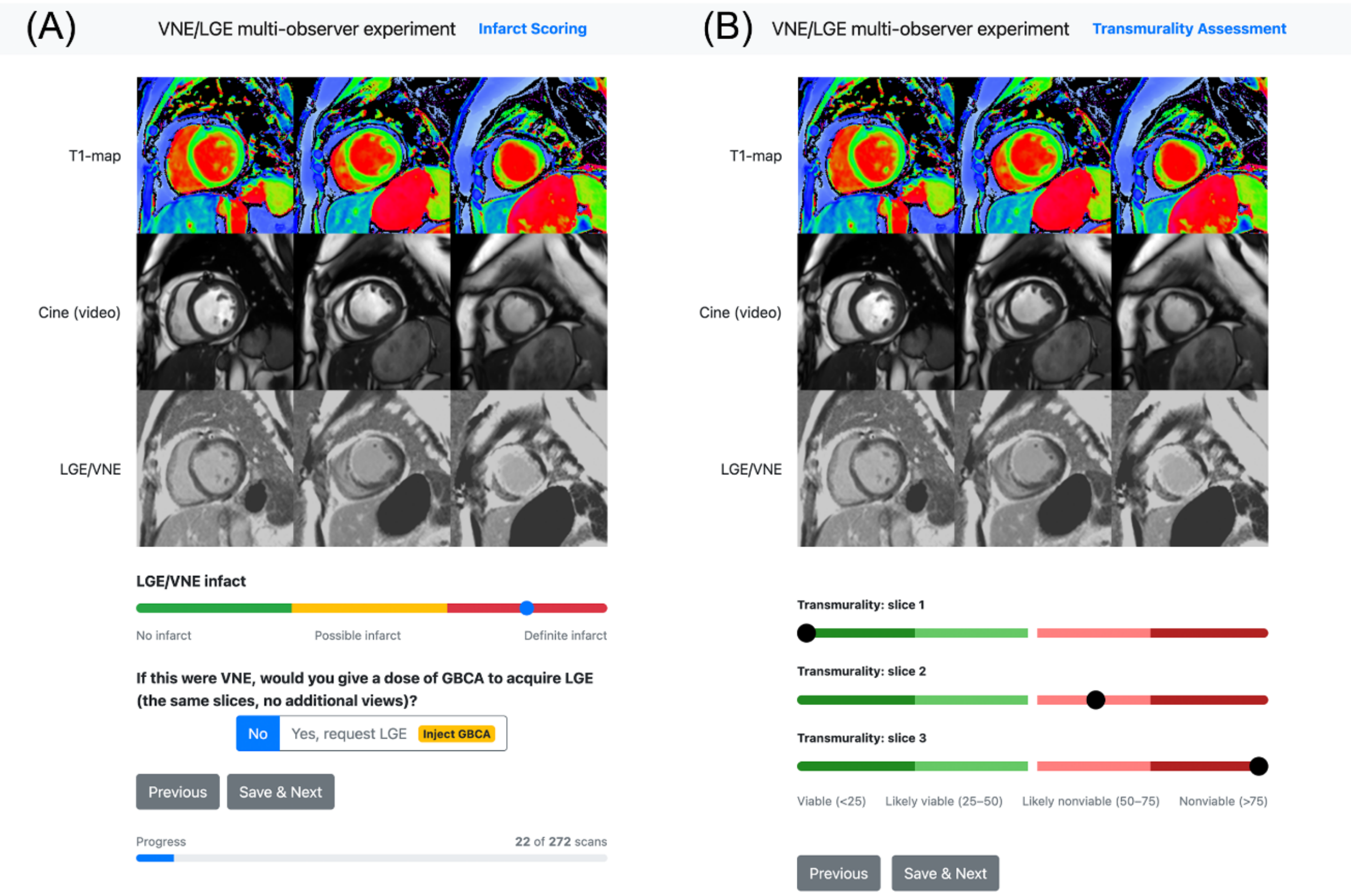


In experiment (A), VNE or LGE images were scored as 'no infarct', 'possible infarct' or 'definite infarct'. This was followed by a query to test the operator's confidence in using the presented scar images for scar assessment, asking "if this were VNE, would you give a dose of GBCA to acquire LGE". if the operator was not confidence with the images and would further request GBCA contrast injection, if the images were VNE.

In experiment (B), scar transmurality of each slice of VNE or LGE was scored based on the readers' visual assessment, on a continuous scale 0-100 guided visually by 4 categories (<25%, 25-50%, 50-75%, >75%).

**Result A: Diagnostic accuracy by clinical readers**

Diagnostic accuracy is reported in the table below as an extension to Table 3 in the main paper. In summary, VNE was considered adequate by the readers without having to proceed to LGE for 69.7% of patients (those for whom the clinical readers answered “No” to “would you give a dose of GBCA to acquire LGE?”). This suggests that VNE may obviate the need for GBCA in over two-thirds of patients for scar assessment, with an average diagnostic accuracy of 93.7%, comparable to that achieved with LGE (93.9%).

| | | Virtual Native Enhancement | | | | | Late Gadolinium Enhancement | | | | | |
|---|---|---|---|---|---|---|---|---|---|---|---|---|
| | | Sens | Spec | PPV | NPV | Acc | Sens | Spec | PPV | NPV | Acc | N |
| All patients | Reader1 | 94 | 69.2 | 83.2 | 87.8 | 84.6 | 97.6 | 92.3 | 95.3 | 96 | 95.6 | 136 |
| | Reader2 | 92.9 | 57.7 | 78 | 83.3 | 79.4 | 94 | 84.6 | 90.8 | 89.8 | 90.4 | |
| | Reader3 | 96.4 | 50 | 75.7 | 89.7 | 78.7 | 96.4 | 80.8 | 89 | 93.3 | 90.4 | |
| | Reader4 | 97.6 | 55.8 | 78.1 | 93.5 | 81.6 | 95.2 | 88.5 | 93 | 92 | 92.6 | |
| | Pooled | 95.2 | 58.2 | 78.6 | 88.3 | 81.1 | 95.8 | 86.5 | 92 | 92.8 | 92.3 | |
| Patients with VNE marked as adequate, i.e. not requesting GBCA | Reader1 | 96.5 | 90 | 94.8 | 93.1 | **94.3** | 96.5 | 93.3 | 96.5 | 93.3 | **95.4** | 87 (64.0%) |
| | Reader2 | 92.4 | 92 | 96.8 | 82.1 | **92.3** | 93.9 | 80 | 92.5 | 83.3 | **90.1** | 91 (66.9%) |
| | Reader3 | 98.6 | 76 | 91.9 | 95 | **92.6** | 100 | 88 | 95.8 | 100 | **96.8** | 94 (69.1%) |
| | Reader4 | 97.4 | 90.3 | 96.1 | 93.3 | **95.3** | 96.1 | 87.1 | 94.8 | 90 | **93.5** | 107 (78.7%) |
| | Pooled | 96.3 | 87.4 | 94.9 | 90.7 | **93.7** | 96.6 | 87.4 | 94.9 | 91.5 | **93.9** | 379 (69.7%) |
| Patients with LGE* (presented as VNE) marked as adequate, i.e. not requesting GBCA | Reader1 | 96.4 | 75.0 | 85.7 | 93.1 | 88.0 | 100.0 | 94.4 | 96.6 | 100.0 | 97.8 | 92 (67.6%) |
| | Reader2 | 95.2 | 60.6 | 81.9 | 87.0 | 83.2 | 96.8 | 100.0 | 100.0 | 94.3 | 97.9 | 95 (69.8%) |
| | Reader3 | 98.6 | 62.9 | 84.5 | 95.7 | 86.9 | 98.6 | 100.0 | 100.0 | 97.2 | 99.1 | 107 (78.8%) |
| | Reader4 | 100.0 | 54.3 | 79.0 | 100.0 | 83.2 | 94.9 | 100.0 | 100.0 | 92.0 | 96.8 | 125 (91.9%) |
| | Pooled | 97.8 | 62.7 | 82.4 | 94.0 | 85.2 | 97.4 | 98.7 | 99.2 | 95.5 | 97.9 | 419 (77.0%) |

*LGE counterpart experiment for reference; the readers did not know whether a scan was LGE or VNE.

**Result B: Scar transmurality by clinical readers**

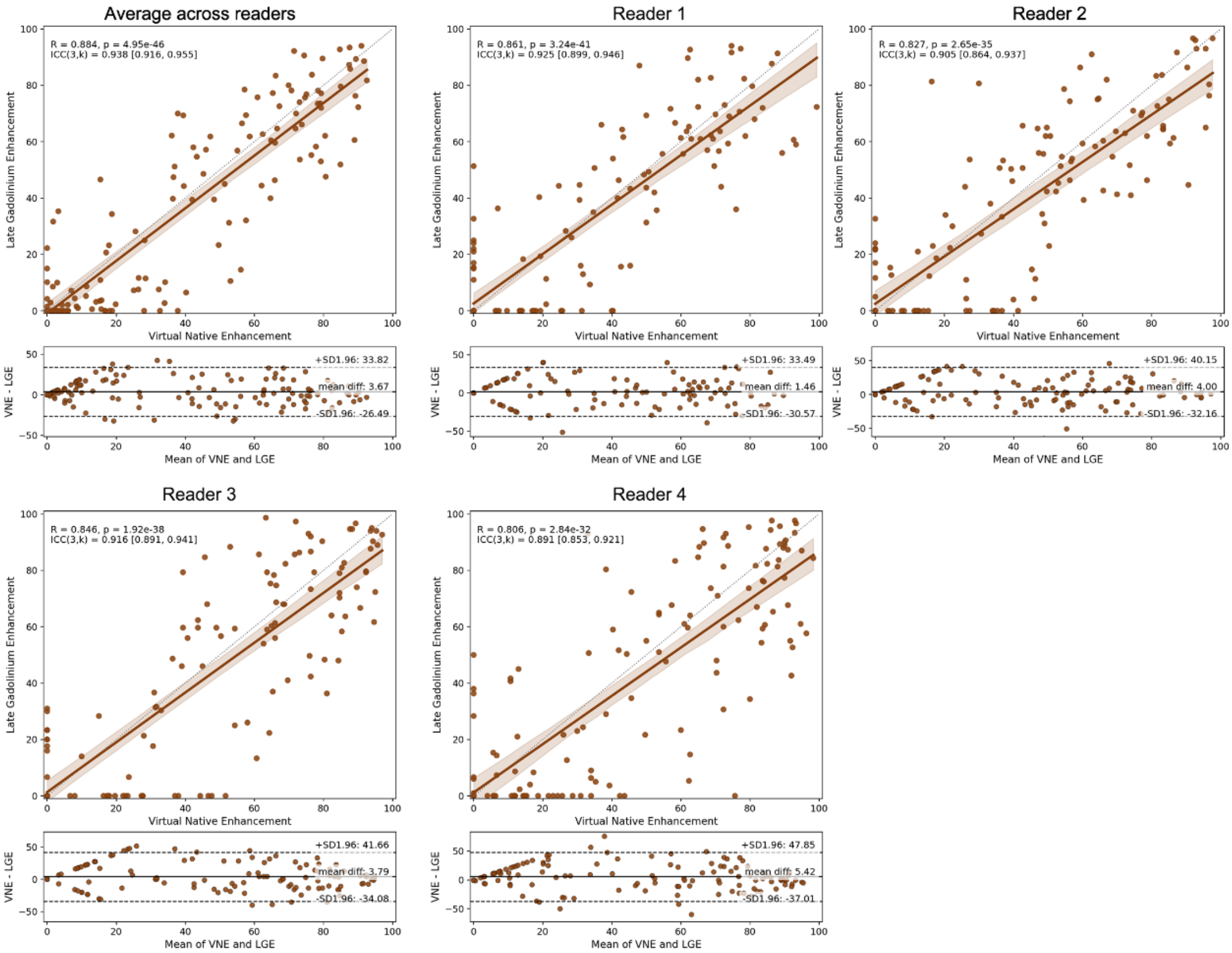


VNE-LGE agreement in scar transmurality scoring achieved an ICC(3,k) between 0.89 and 0.93, and a correlation coefficient R between 0.81 and 0.86. Comparing the mean scores averaged across readers, the VNE-LGE agreement in scoring transmurality was ICC(3,k)=0.94, R=0.88.

Inter-observer variability was ICC(3,k)=0.915 [0.882, 0.942] and categorical Fleiss k=0.770 (with threshold at 50% transmurality) for the four readers.

## Section V: Quantitative analysis of scar burden

LGE and VNE quantification was semi-automatic. Images were pre-contoured automatically using a machine learning segmentation model by RG (1), followed by manual inspection of all data and corrections where necessary by an image analyst (GV) in Oxford. Myocardial regions of interest were manually contoured within scarred myocardium (hyperintensity on LGE and VNE) and remote myocardium (area without hyperintensities, typically opposite of the scar area), to derive the scar volume using the full width at half maximum method (2). In the absence of scarred myocardium, a blood pool region was annotated to calculate the hyperintensity. Image contrast to noise ratio was calculated as the signal intensity difference between scar and remote myocardium divided by the standard deviations in remote myocardium. Scar burden volume fraction was quantified for each patient as the sum of scar areas divided by the total left ventricular myocardial area in all matching LGE and VNE short-axis slices. Myocardial segments and territories of infarct were analyzed and visualized using the 16-segmental model. Transmurality was measured using the centerline chord method (3,4). Segmental transmurality was calculated by averaging the chords that had at least 1% of scar extent (3,4).

**References:**


[1] Gonzales RA, Ibáñez DH, Hann E, Popescu IA, Burrage MK, Lee YP et al. Quality control-driven deep ensemble for accountable automated segmentation of cardiac magnetic resonance LGE and VNE images. Frontiers in Cardiovascular Medicine 2023;10:1213290.

[2] Gräni C, Eichhorn C, Bière L, Kaneko K, Murthy VL, Agarwal V et al. Comparison of myocardial fibrosis quantification methods by cardiovascular magnetic resonance imaging for risk stratification of patients with suspected myocarditis. J Cardiovasc Magn R 2019;21:14.

[3] Zhang Q, Burrage MK, Shanmuganathan M, Gonzales RA, Lukaschuk E, Thomas KE et al. Artificial Intelligence for Contrast-Free MRI: Scar Assessment in Myocardial Infarction Using Deep Learning-Based Virtual Native Enhancement. Circulation 2022;146:1492-1503.

[4] Wang G, Lee S-E, Yang Q, Sadras V, Patel S, Yang H-J et al. Multicenter study on the diagnostic performance of native-T1 cardiac magnetic resonance of chronic myocardial infarctions at 3T. Circulation: Cardiovascular Imaging 2020;13:e009894.

## Section VI: Additional Supplemental Figures and Tables

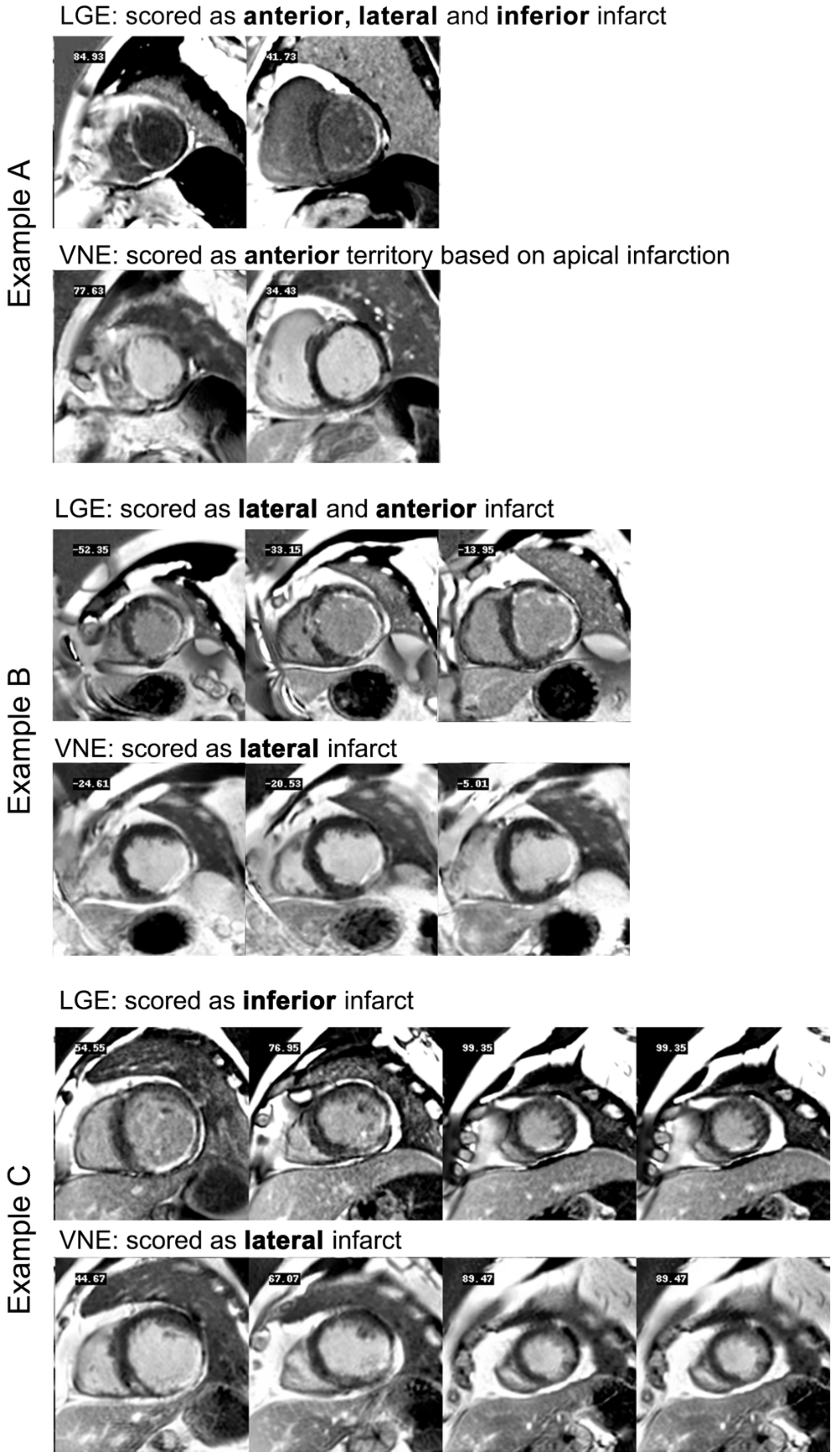


**Figure S1: Examples of disagreements in scoring infarct territories.** The main reasons were the inter-observer discrepancies in labelling multi-territory infarcts **(A, B)** and in assigning an infarction territory as predominantly inferior or lateral **(C)**.

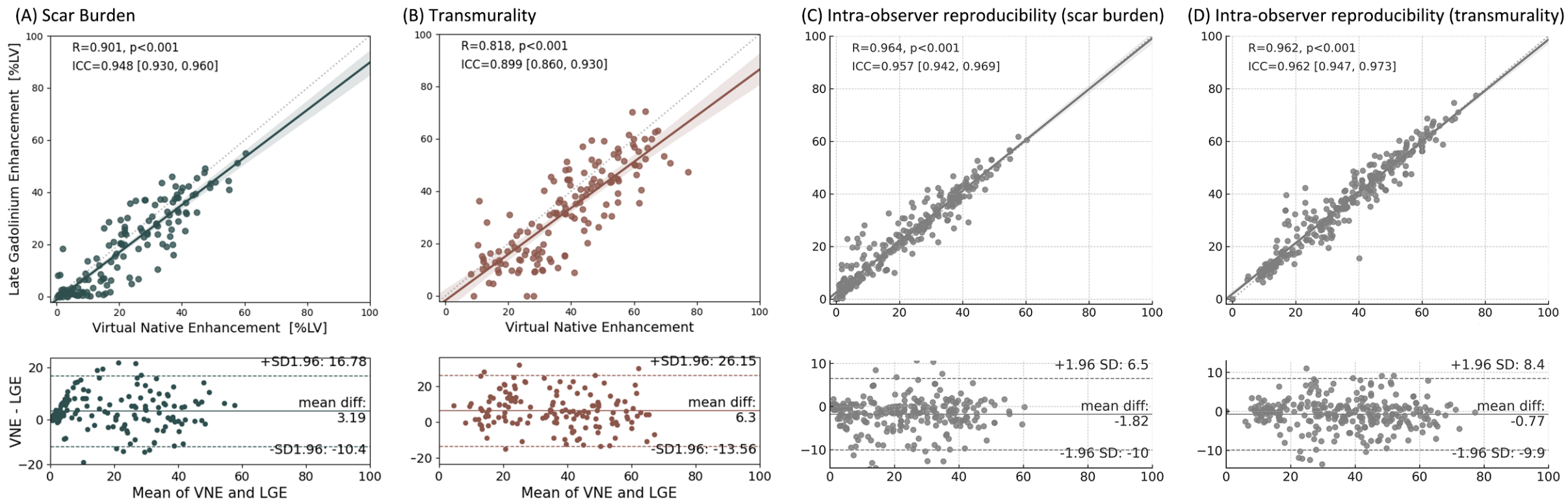


**Figure S2: Intra-observer reproducibility of quantitative analysis.** Intra-observer reproducibility of the semi-automatic quantification was R=0.96 (ICC=0.96) for per-patient scar burden, and R=0.96 (ICC=0.96) for per-patient transmurality. Intra-observer variabilities were ±1.96 SDs intervals of 17% to 18%, accounting for approximately half of inter-method variabilities (±1.96 SDs intervals of 27% to 40%). Future AI development can enable fully automated, robust contouring and quantification of LGE and VNE images.

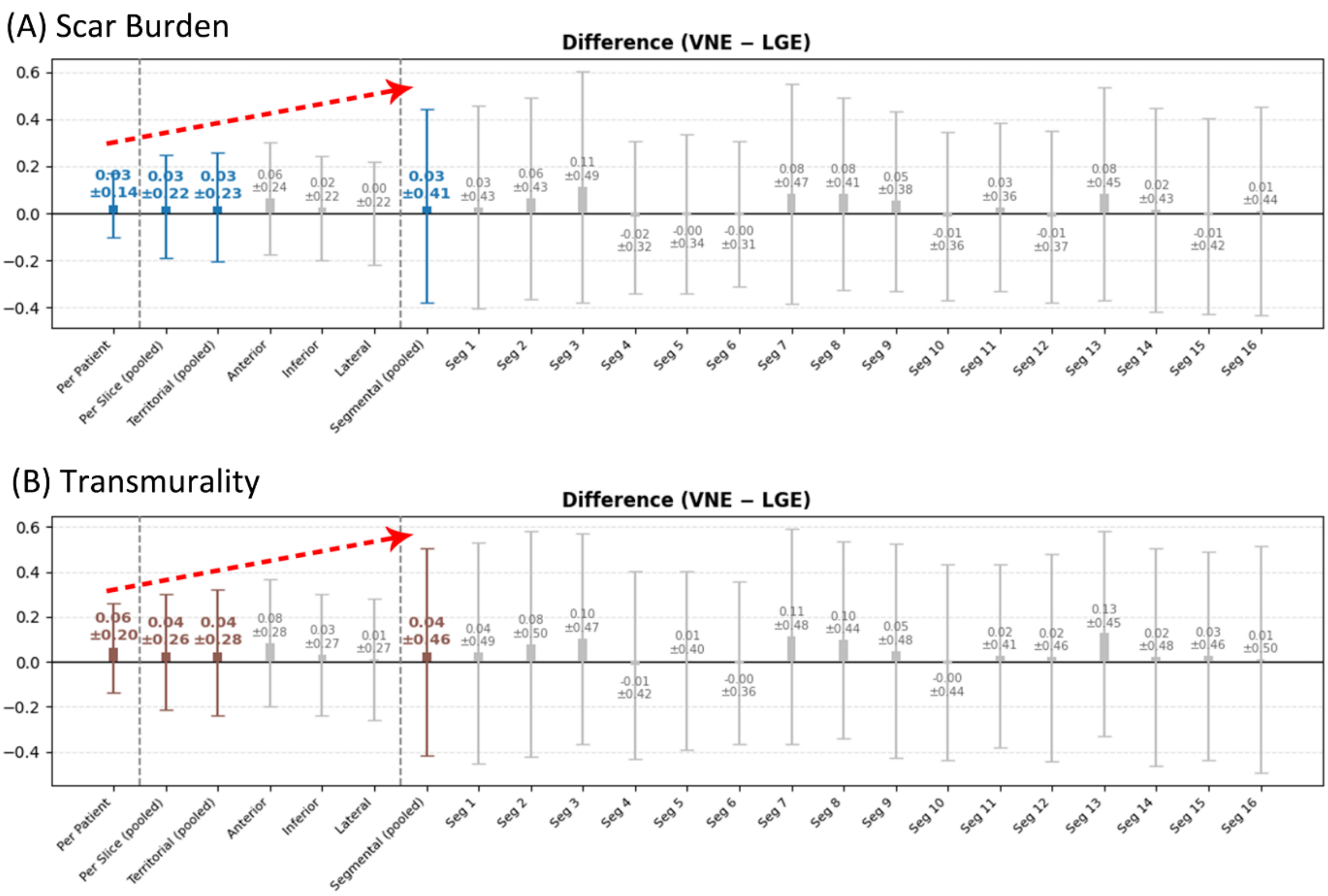


**Figure S3**: Per-patient, per-slice, per-territory and per-segment differences between VNE and LGE. The ±1.96 SD estimate of confidence interval increased from per-patient to per-slice, per-territory, per-segment measurements, from ±0.14 to ±0.22, ±0.23, ±0.41 for scar burden, and from ±0.2 to ±0.26, ±0.28, ±0.46 for transmurality. Note that both scar burden and transmurality are limited to 0-1 range, which substantially clips any variation except for those very close to 50%. For this reason, the per-segment analyses are not presented.

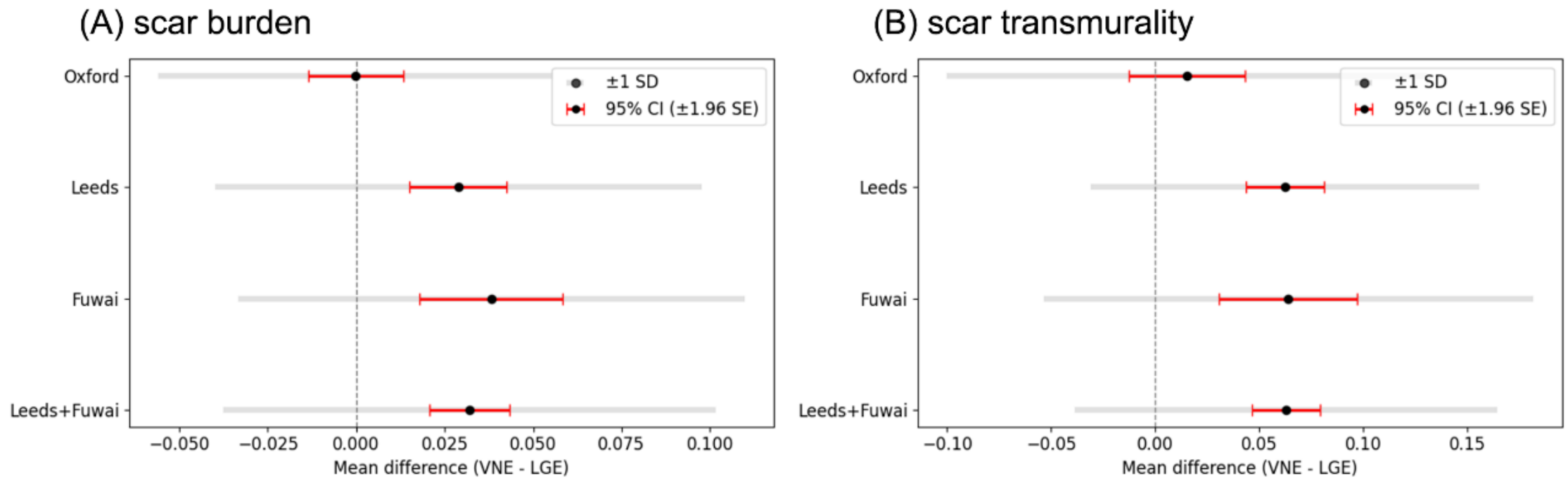


**Figure S4**: Forest plot comparing VNE and LGE scar burden and scar transmurality across study sites. Oxford data from the original single-center VNE development study are shown for reference [1]. (A) For scar burden quantification, VNE performance was consistent between Leeds and Fuwai, with 3. 2% bias when comparing the pooled Leeds+Fuwai data with the Oxford internal validation data (published in [1]). The 3.2% bias in scar burden volume fraction is clinically acceptable, given the range of the observed sample standard deviation of 5.6-7.2% (grey bars), and reported 10% infarct size bias between LGE quantification methods [2]. (B) For scar transmurality, VNE performance was consistent between Leeds and Fuwai. VNE reported 6.3% higher transmurality than LGE in the pooled Leeds+Fuwai data, compared with 1.5% higher than LGE in the Oxford internal validation data [1]. Although this overestimation is small relative to the categorical clinical assessment (<25%, 25-50%, 50-75%, >75%), it should be noted in clinical interpretation.

There was no significant difference in the LGE-VNE agreement between the two cohorts, despite different ethnic composition (predominantly British in Leeds and Chinese in Fuwai), different patient characteristics (such as BMI 29.2 vs 25.7 kg/m2, and smoking history 22.4% vs 62.5%), different magnetic field strengths of MR scanners (1.5 Tesla vs 3 Tesla), and different contrast agents used (gadobutrol vs gadopentetate dimeglumine).

[1] Zhang, Q. et al. (2022). Artificial intelligence for contrast-free MRI: scar assessment in myocardial infarction using deep learning–based virtual native enhancement. Circulation, 146(20), 1492-1503.
[2] Klem, Igor, et al. (2016) "Sources of variability in quantification of cardiovascular magnetic resonance infarct size-reproducibility among three core laboratories." Journal of Cardiovascular Magnetic Resonance 19.1 (2016): 62.

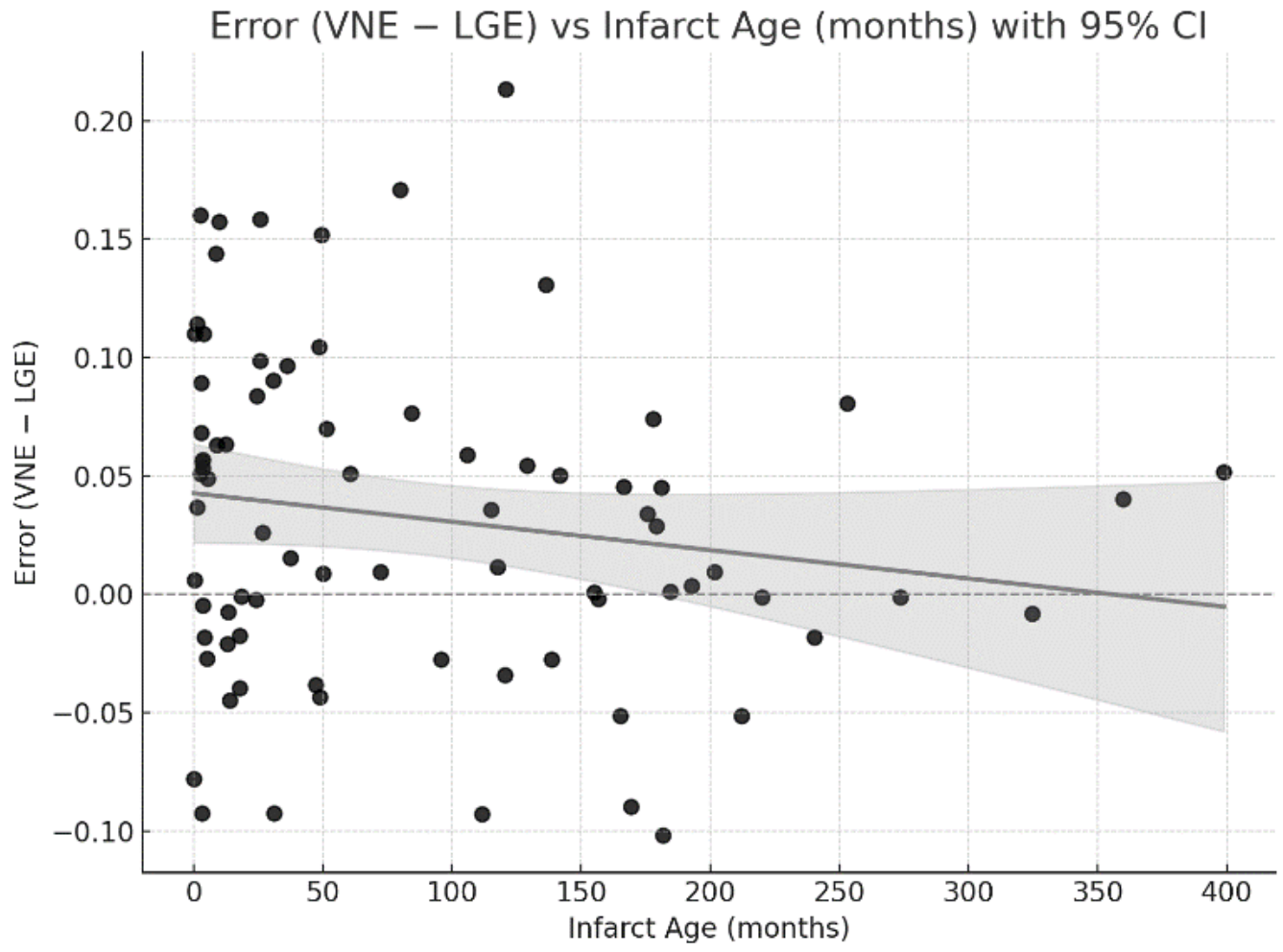


**Figure S5:** Difference between VNE and LGE scar burden plotted against infarct age in this chronic myocardial infarction cohort (based on N=79 cases of known infarct age). Infarct ages are 49 [10, 156] months. The regression line is plotted for visual guidance, and there was no strong correlation ($p=0.145$).

**Table S1: Agreement between VNE and LGE on myocardial territories of infarction (anterior, inferior, lateral) derived from the distribution of myocardial scar burden, according to the AHA 16-segment model.**

| **(A) All patients (N=136)** | | VNE | | |
|---|---|---|---|---|
| | | Anterior | Inferior | Lateral |
| LGE | Anterior | 47 | 0 | 1 |
| | Inferior | 1 | 10 | 2 |
| | Lateral | 3 | 2 | 13 |

| **(B) Leeds (N=88)** | | VNE | | |
|---|---|---|---|---|
| | | Anterior | Inferior | Lateral |
| LGE | Anterior | 13 | 0 | 0 |
| | Inferior | 1 | 6 | 2 |
| | Lateral | 1 | 0 | 8 |

| **(C) Fuwai (N=48)** | | VNE | | |
|---|---|---|---|---|
| | | Anterior | Inferior | Lateral |
| LGE | Anterior | 34 | 0 | 1 |
| | Inferior | 0 | 4 | 0 |
| | Lateral | 2 | 2 | 5 |

VNE-LGE agreement rates were (a) overall 88.6% for all patients, (B) 87.1% for Leeds and (C) 89.5% for Fuwai patients.

## Section VII: T1-map protocols underpinning VNE in Siemens format

Protocol TOC (OxShMOLLI T1-map details shown only once below TOC):

**Table of contents**

- \\USER
  - T1Map_cardio_OxfordC2P_ShMOLLI
    - Template_Clinical
      - OxShmolli
        - OxShmolli
      - Templates_VNE
        - OxShmolli
        - cine_tf2d12_retro_iPAT- no dist corr
        - Late_Gad_Trufi_psir

| \\USER\T1Map_cardio_OxfordC2P_ShMOLLI\Template_Clinical\OxShmolli\OxShmolli |
|---|
| TA: 6.1 s PM: REF Voxel size: 0.9×0.9×8.0 mmPAT: 2 Rel. SNR: 1.00 : tfi |

### Properties

| | |
|---|---|
| Prio recon | Off |
| Load images to viewer | On |
| Inline movie | Off |
| Auto store images | On |
| Load images to stamp segments | Off |
| Load images to graphic segments | On |
| Auto open inline display | Off |
| Auto close inline display | Off |
| Start measurement without further preparation | Off |
| Wait for user to start | On |
| Start measurements | Single measurement |

### Routine

| | |
|---|---|
| Slice group | 1 |
| Slices | 1 |
| Dist. factor | 20 % |
| Position | L11.8 P38.0 F32.0 mm |
| Orientation | Transversal |
| Phase enc. dir. | A >> P |
| AutoAlign | --- |
| Phase oversampling | 0 % |
| FoV read | 360 mm |
| FoV phase | 75.0 % |
| Slice thickness | 8.0 mm |
| TR | 379.40 ms |
| TE | 1.07 ms |
| Averages | 1 |
| Concatenations | 1 |
| Filter | Raw filter, Distortion Corr.(2D) |
| Coil elements | BO2,3;SP2,3 |

### Contrast - Common

| | |
|---|---|
| TR | 379.40 ms |
| TE | 1.07 ms |
| Magn. preparation | Non-sel. IR |
| TI | 260 ms |
| Flip angle | 35 deg |
| Fat suppr. | None |
| Wrap-up Magn. | None |

### Contrast - Dynamic

| | |
|---|---|
| Averages | 1 |
| Averaging mode | Short term |
| Reconstruction | Magn./Phase |
| Measurements | 1 |
| Multiple series | Off |

### Resolution - Common

| | |
|---|---|
| FoV read | 360 mm |
| FoV phase | 75.0 % |
| Slice thickness | 8.0 mm |
| Base resolution | 192 |
| Phase resolution | 100 % |
| Phase partial Fourier | 6/8 |
| Trajectory | Cartesian |
| Interpolation | On |

### Resolution - iPAT

| | |
|---|---|
| PAT mode | GRAPPA |
| Accel. factor PE | 2 |
| Ref. lines PE | 24 |
| Reference scan mode | Integrated |

### Resolution - Filter Image

| | |
|---|---|
| Image Filter | Off |
| Distortion Corr. | On |
| Mode | 2D |
| Unfiltered images | Off |
| Prescan Normalize | Off |
| Normalize | Off |
| B1 filter | Off |

### Resolution - Filter Rawdata

| | |
|---|---|
| Raw filter | On |
| Elliptical filter | Off |
| POCS | Off |

### Geometry - Common

| | |
|---|---|
| Slice group | 1 |
| Slices | 1 |
| Dist. factor | 20 % |
| Position | L11.8 P38.0 F32.0 mm |
| Orientation | Transversal |
| Phase enc. dir. | A >> P |
| FoV read | 360 mm |
| FoV phase | 75.0 % |
| Slice thickness | 8.0 mm |
| TR | 379.40 ms |
| Multi-slice mode | Sequential |
| Series | Interleaved |
| Concatenations | 1 |

### Geometry - AutoAlign

| | |
|---|---|
| Slice group | 1 |
| Position | L11.8 P38.0 F32.0 mm |
| Orientation | Transversal |
| Phase enc. dir. | A >> P |
| AutoAlign | --- |
| Initial Position | L11.8 P38.0 F32.0 |
| L | 11.8 mm |
| P | 38.0 mm |
| F | 32.0 mm |
| Initial Rotation | 0.00 deg |
| Initial Orientation | Transversal |

### Geometry - Saturation

| | |
|---|---|
| Fat suppr. | None |
| Wrap-up Magn. | None |
| Special sat. | None |

### Geometry - Navigator

### Geometry - Tim Planning Suite

| | |
|---|---|
| Set-n-Go Protocol | Off |
| Table position | H |
| Table position | 0 mm |
| Inline Composing | Off |

## System - Miscellaneous

| | |
|---|---|
| Positioning mode | REF |
| Table position | H |
| Table position | 0 mm |
| MSMA | S - C - T |
| Sagittal | R >> L |
| Coronal | A >> P |
| Transversal | F >> H |
| Coil Combine Mode | Adaptive Combine |
| Save uncombined | Off |
| Matrix Optimization | Off |
| Coil Focus | Flat |
| AutoAlign | --- |
| Coil Select Mode | Default |

## System - Adjustments

| | |
|---|---|
| B0 Shim mode | Cardiac |
| B1 Shim mode | TrueForm |
| Adjust with body coil | On |
| Confirm freq. adjustment | Off |
| Assume Dominant Fat | Off |
| Assume Silicone | Off |
| Adjustment Tolerance | Auto |

## System - Adjust Volume

| | |
|---|---|
| ! Position | L13.6 P37.8 H0.0 mm |
| ! Orientation | Transversal |
| ! Rotation | 0.00 deg |
| ! A >> P | 150 mm |
| ! R >> L | 150 mm |
| ! F >> H | 150 mm |
| Reset | Off |

## System - Tx/Rx

| | |
|---|---|
| Frequency 1H | 123.262000 MHz |
| Correction factor | 1 |
| Gain | High |
| Img. Scale Cor. | 1.000 |
| Reset | Off |
| ? Ref. amplitude 1H | 0.000 V |

## Physio - Signal1

| | |
|---|---|
| 1st Signal/Mode | ECG/Trigger |
| Average cycle | 681 ± 105 ms |
| Average cycle | No Signal ms |
| Captured cycle | 681 ± 105 ms |
| Acquisition window | 679 ms |
| Trigger pulse | 1 |
| Trigger delay | 260 ms |
| TR | 379.40 ms |
| Concatenations | 1 |
| Segments | 84 |
| Phases | 1 |
| Adaptive Triggering | Off |

## Physio - Cardiac

| | |
|---|---|
| Tagging | None |
| Magn. preparation | Non-sel. IR |
| TI | 260 ms |
| Fat suppr. | None |
| Dark blood | Off |
| FoV read | 360 mm |
| FoV phase | 75.0 % |
| Phase resolution | 100 % |
| Cine | Off |

## Physio - Cardiac

| | |
|---|---|
| Trajectory | Cartesian |
| Dummy heartbeats | 0 |
| Motion Correction | Standard |

## Physio - PACE

| | |
|---|---|
| Resp. control | Off |
| Concatenations | 1 |

## Inline - Common

| | |
|---|---|
| Subtract | Off |
| Measurements | 1 |
| StdDev | Off |
| Motion Correction | Standard |
| Save original images | On |

## Inline - Cardiac

| | |
|---|---|
| Inline Evaluation | T1 map |
| Magn. preparation | Non-sel. IR |
| Num. of preps | 3 |
| Sampling duration 1 | 5 beats |
| Sampling duration 2 | 1 beats |
| Sampling duration 3 | 1 beats |
| Contrasts | 1 |
| TE | 1.07 ms |
| TR | 379.40 ms |
| Recovery duration 1 | 1 beats |
| Recovery duration 2 | 1 beats |
| Recovery duration 3 | 0 beats |
| Motion Correction | Standard |
| Save original images | On |

## Inline - MIP

| | |
|---|---|
| MIP-Sag | Off |
| MIP-Cor | Off |
| MIP-Tra | Off |
| MIP-Time | Off |
| Save original images | On |

## Inline - Composing

| | |
|---|---|
| Inline Composing | Off |
| Distortion Corr. | On |
| Mode | 2D |
| Unfiltered images | Off |

## Sequence - Part 1

| | |
|---|---|
| Introduction | Off |
| Dimension | 2D |
| Reordering | Linear |
| Asymmetric echo | Weak |
| Contrasts | 1 |
| Optimization | Min. TE TR |
| Multi-slice mode | Sequential |
| Echo spacing | 2.6 ms |
| Sequence type | Trufi |
| Bandwidth | 898 Hz/Px |

## Sequence - Part 2

| | |
|---|---|
| Define | Shots |
| Shots per slice | 1 |
| Segments | 84 |
| Trufi delta freq. | 0 Hz |
| RF pulse type | Fast |
| Gradient mode | Fast |

### Sequence - Part 2

| | |
|---|---|
| Excitation | Slice-sel. |
| Flip angle mode | Constant |
| Cine | Off |

### Sequence - Assistant

| | |
|---|---|
| Mode | Off |
| Allowed delay | 0 s |

| \\USER\T1Map_cardio_OxfordC2P_ShMOLLI\Template_Clinical\Templates_VNE\OxShmolli |
|---|
| TA: 6.1 s PM: REF Voxel size: 0.9×0.9×8.0 mmPAT: 2 Rel. SNR: 1.00 : tfi |

## Properties

| | |
|---|---|
| Prio recon | Off |
| Load images to viewer | On |
| Inline movie | Off |
| Auto store images | On |
| Load images to stamp segments | Off |
| Load images to graphic segments | On |
| Auto open inline display | Off |
| Auto close inline display | Off |
| Start measurement without further preparation | Off |
| Wait for user to start | Off |
| Start measurements | Single measurement |

## Routine

| | |
|---|---|
| Slice group | 1 |
| Slices | 1 |
| Dist. factor | 20 % |
| Position | L36.0 A4.2 F37.4 mm |
| Orientation | S > T42.6 > C33.6 |
| Phase enc. dir. | A >> P |
| AutoAlign | --- |
| Phase oversampling | 0 % |
| FoV read | 360 mm |
| FoV phase | 75.0 % |
| Slice thickness | 8.0 mm |
| TR | 379.40 ms |
| TE | 1.07 ms |
| Averages | 1 |
| Concatenations | 1 |
| Filter | Raw filter, Distortion Corr.(2D) |
| Coil elements | BO1-3;SP1-3 |

## Contrast - Common

| | |
|---|---|
| TR | 379.40 ms |
| TE | 1.07 ms |
| Magn. preparation | Non-sel. IR |
| TI | 260 ms |
| Flip angle | 35 deg |
| Fat suppr. | None |
| Wrap-up Magn. | None |

## Contrast - Dynamic

| | |
|---|---|
| Averages | 1 |
| Averaging mode | Short term |
| Reconstruction | Magn./Phase |
| Measurements | 1 |
| Multiple series | Off |

## Resolution - Common

| | |
|---|---|
| FoV read | 360 mm |
| FoV phase | 75.0 % |
| Slice thickness | 8.0 mm |
| Base resolution | 192 |
| Phase resolution | 100 % |
| Phase partial Fourier | 6/8 |
| Trajectory | Cartesian |
| Interpolation | On |

## Resolution - iPAT

| | |
|---|---|
| PAT mode | GRAPPA |
| Accel. factor PE | 2 |
| Ref. lines PE | 24 |
| Reference scan mode | Integrated |

## Resolution - Filter Image

| | |
|---|---|
| Image Filter | Off |
| Distortion Corr. | On |
| Mode | 2D |
| Unfiltered images | Off |
| Prescan Normalize | Off |
| Normalize | Off |
| B1 filter | Off |

## Resolution - Filter Rawdata

| | |
|---|---|
| Raw filter | On |
| Elliptical filter | Off |
| POCS | Off |

## Geometry - Common

| | |
|---|---|
| Slice group | 1 |
| Slices | 1 |
| Dist. factor | 20 % |
| Position | L36.0 A4.2 F37.4 mm |
| Orientation | S > T42.6 > C33.6 |
| Phase enc. dir. | A >> P |
| FoV read | 360 mm |
| FoV phase | 75.0 % |
| Slice thickness | 8.0 mm |
| TR | 379.40 ms |
| Multi-slice mode | Sequential |
| Series | Base To Apex |
| Concatenations | 1 |

## Geometry - AutoAlign

| | |
|---|---|
| Slice group | 1 |
| Position | L36.0 A4.2 F37.4 mm |
| Orientation | S > T42.6 > C33.6 |
| Phase enc. dir. | A >> P |
| AutoAlign | --- |
| Initial Position | L36.0 A4.2 F37.4 |
| L | 36.0 mm |
| A | 4.2 mm |
| F | 37.4 mm |
| Initial Rotation | 26.97 deg |
| Initial Orientation | S > T |
| S > T | 42.6 |
| > C | 33.6 |

## Geometry - Saturation

| | |
|---|---|
| Fat suppr. | None |
| Wrap-up Magn. | None |
| Special sat. | None |

## Geometry - Navigator

## Geometry - Tim Planning Suite

| | |
|---|---|
| Set-n-Go Protocol | Off |
| Table position | H |
| Table position | 0 mm |

## Geometry - Tim Planning Suite

| | |
|---|---|
| Inline Composing | Off |

## System - Miscellaneous

| | |
|---|---|
| Positioning mode | REF |
| Table position | H |
| Table position | 0 mm |
| MSMA | S - C - T |
| Sagittal | R >> L |
| Coronal | A >> P |
| Transversal | F >> H |
| Coil Combine Mode | Adaptive Combine |
| Save uncombined | Off |
| Matrix Optimization | Off |
| Coil Focus | Flat |
| AutoAlign | --- |
| Coil Select Mode | On - AutoCoilSelect |

## System - Adjustments

| | |
|---|---|
| B0 Shim mode | Cardiac |
| B1 Shim mode | TrueForm |
| Adjust with body coil | On |
| Confirm freq. adjustment | Off |
| Assume Dominant Fat | Off |
| Assume Silicone | Off |
| Adjustment Tolerance | Auto |

## System - Adjust Volume

| | |
|---|---|
| ! Position | L46.1 A10.4 F37.2 mm |
| ! Orientation | S > T43.2 > C26.0 |
| ! Rotation | 22.37 deg |
| ! A >> P | 98 mm |
| ! F >> H | 107 mm |
| ! R >> L | 134 mm |
| Reset | Off |

## System - Tx/Rx

| | |
|---|---|
| Frequency 1H | 123.262000 MHz |
| Correction factor | 1 |
| Gain | High |
| Img. Scale Cor. | 1.000 |
| Reset | Off |
| ? Ref. amplitude 1H | 0.000 V |

## Physio - Signal1

| | |
|---|---|
| 1st Signal/Mode | ECG/Trigger |
| Average cycle | 681 ± 105 ms |
| Average cycle | No Signal ms |
| Captured cycle | 681 ± 105 ms |
| Acquisition window | 640 ms |
| Trigger pulse | 1 |
| Trigger delay | 260 ms |
| TR | 379.40 ms |
| Concatenations | 1 |
| Segments | 84 |
| Phases | 1 |
| Adaptive Triggering | Off |

## Physio - Cardiac

| | |
|---|---|
| Tagging | None |
| Magn. preparation | Non-sel. IR |
| TI | 260 ms |
| Fat suppr. | None |
| Dark blood | Off |
| FoV read | 360 mm |
| FoV phase | 75.0 % |
| Phase resolution | 100 % |
| Cine | Off |
| Trajectory | Cartesian |
| Dummy heartbeats | 0 |
| Motion Correction | Standard |

## Physio - PACE

| | |
|---|---|
| Resp. control | Breath-hold |
| Concatenations | 1 |

## Inline - Common

| | |
|---|---|
| Subtract | Off |
| Measurements | 1 |
| StdDev | Off |
| Motion Correction | Standard |
| Save original images | On |

## Inline - Cardiac

| | |
|---|---|
| Inline Evaluation | T1 map |
| Magn. preparation | Non-sel. IR |
| Num. of preps | 3 |
| Sampling duration 1 | 5 beats |
| Sampling duration 2 | 1 beats |
| Sampling duration 3 | 1 beats |
| Contrasts | 1 |
| TE | 1.07 ms |
| TR | 379.40 ms |
| Recovery duration 1 | 1 beats |
| Recovery duration 2 | 1 beats |
| Recovery duration 3 | 0 beats |
| Motion Correction | Standard |
| Save original images | On |

## Inline - MIP

| | |
|---|---|
| MIP-Sag | Off |
| MIP-Cor | Off |
| MIP-Tra | Off |
| MIP-Time | Off |
| Save original images | On |

## Inline - Composing

| | |
|---|---|
| Inline Composing | Off |
| Distortion Corr. | On |
| Mode | 2D |
| Unfiltered images | Off |

## Sequence - Part 1

| | |
|---|---|
| Introduction | Off |
| Dimension | 2D |
| Reordering | Linear |
| Asymmetric echo | Weak |
| Contrasts | 1 |
| Optimization | Min. TE TR |
| Multi-slice mode | Sequential |
| Echo spacing | 2.6 ms |
| Sequence type | Trufi |
| Bandwidth | 898 Hz/Px |

## Sequence - Part 2

| | |
|---|---|
| Define | Shots |
| Shots per slice | 1 |
| Segments | 84 |

### Sequence - Part 2

| | |
|---|---|
| Trufi delta freq. | 0 Hz |
| RF pulse type | Fast |
| Gradient mode | Fast |
| Excitation | Slice-sel. |
| Flip angle mode | Constant |
| Cine | Off |

### Sequence - Assistant

| | |
|---|---|
| Mode | Off |
| Allowed delay | 0 s |

| \\USER\T1Map_cardio_OxfordC2P_ShMOLLI\Template_Clinical\Templates_VNE\cine_tf2d12_retro_iPAT- no dist corr |
|---|
| TA: 5.4 s PM: REF Voxel size: 1.8×1.8×8.0 mmPAT: 3 Rel. SNR: 1.00 : tfi |

## Properties

| | |
|---|---|
| Prio recon | Off |
| Load images to viewer | On |
| Inline movie | On |
| Auto store images | On |
| Load images to stamp segments | On |
| Load images to graphic segments | On |
| Auto open inline display | Off |
| Auto close inline display | Off |
| Start measurement without further preparation | On |
| Wait for user to start | Off |
| Start measurements | Single measurement |

## Routine

| | |
|---|---|
| Slice group | 1 |
| Slices | 1 |
| Dist. factor | 25 % |
| Position | L36.0 A4.2 F37.4 mm |
| Orientation | S > T42.6 > C33.6 |
| Phase enc. dir. | A >> P |
| AutoAlign | --- |
| Phase oversampling | 0 % |
| FoV read | 380 mm |
| FoV phase | 75.0 % |
| Slice thickness | 8.0 mm |
| TR | 37.80 ms |
| TE | 1.38 ms |
| Averages | 1 |
| Concatenations | 1 |
| Filter | Distortion Corr.(2D), Prescan Normalize, Image Filter |
| Coil elements | BO1-3;SP1-3 |

## Contrast - Common

| | |
|---|---|
| TR | 37.80 ms |
| TE | 1.38 ms |
| Magn. preparation | None |
| Flip angle | 52 deg |
| Fat suppr. | None |
| Wrap-up Magn. | Restore |

## Contrast - Dynamic

| | |
|---|---|
| Averages | 1 |
| Averaging mode | Short term |
| Reconstruction | Magnitude |
| Measurements | 1 |
| Multiple series | Each slice |

## Resolution - Common

| | |
|---|---|
| FoV read | 380 mm |
| FoV phase | 75.0 % |
| Slice thickness | 8.0 mm |
| Base resolution | 208 |
| Phase resolution | 80 % |
| Phase partial Fourier | Off |
| Trajectory | Cartesian |
| View sharing | Off |
| Interpolation | Off |

## Resolution - iPAT

| | |
|---|---|
| PAT mode | GRAPPA |
| Accel. factor PE | 3 |
| Ref. lines PE | 45 |
| Reference scan mode | Integrated |

## Resolution - Filter Image

| | |
|---|---|
| Image Filter | On |
| ! Intensity | Medium |
| Edge Enhancement | 1 |
| Smoothing | 3 |
| Unfiltered images | Off |
| Distortion Corr. | On |
| Mode | 2D |
| Unfiltered images | Off |
| Prescan Normalize | On |
| Unfiltered images | Off |
| Normalize | Off |
| B1 filter | Off |

## Resolution - Filter Rawdata

| | |
|---|---|
| Raw filter | Off |
| Elliptical filter | Off |
| POCS | Off |

## Geometry - Common

| | |
|---|---|
| Slice group | 1 |
| Slices | 1 |
| Dist. factor | 25 % |
| Position | L36.0 A4.2 F37.4 mm |
| Orientation | S > T42.6 > C33.6 |
| Phase enc. dir. | A >> P |
| FoV read | 380 mm |
| FoV phase | 75.0 % |
| Slice thickness | 8.0 mm |
| TR | 37.80 ms |
| Multi-slice mode | Sequential |
| Series | Base To Apex |
| Concatenations | 1 |

## Geometry - AutoAlign

| | |
|---|---|
| Slice group | 1 |
| Position | L36.0 A4.2 F37.4 mm |
| Orientation | S > T42.6 > C33.6 |
| Phase enc. dir. | A >> P |
| AutoAlign | --- |
| Initial Position | L36.0 A4.2 F37.4 |
| L | 36.0 mm |
| A | 4.2 mm |
| F | 37.4 mm |
| Initial Rotation | 26.97 deg |
| Initial Orientation | S > T |
| S > T | 42.6 |
| > C | 33.6 |

## Geometry - Saturation

| | |
|---|---|
| Fat suppr. | None |
| Wrap-up Magn. | Restore |
| Special sat. | None |

## Geometry - Navigator

## Geometry - Tim Planning Suite

| | |
|---|---|
| Set-n-Go Protocol | Off |
| Table position | H |
| Table position | 0 mm |
| Inline Composing | Off |

## System - Miscellaneous

| | |
|---|---|
| Positioning mode | REF |
| Table position | H |
| Table position | 0 mm |
| MSMA | S - C - T |
| Sagittal | R >> L |
| Coronal | A >> P |
| Transversal | F >> H |
| Coil Combine Mode | Sum of Squares |
| Save uncombined | Off |
| Matrix Optimization | Off |
| Coil Focus | Flat |
| AutoAlign | --- |
| Coil Select Mode | On - AutoCoilSelect |

## System - Adjustments

| | |
|---|---|
| B0 Shim mode | Cardiac |
| B1 Shim mode | TrueForm |
| Adjust with body coil | Off |
| Confirm freq. adjustment | Off |
| Assume Dominant Fat | Off |
| Assume Silicone | Off |
| Adjustment Tolerance | Auto |

## System - Adjust Volume

| | |
|---|---|
| ! Position | L46.1 A10.4 F37.2 mm |
| ! Orientation | S > T43.2 > C26.0 |
| ! Rotation | 22.37 deg |
| ! A >> P | 98 mm |
| ! F >> H | 107 mm |
| ! R >> L | 134 mm |
| Reset | Off |

## System - Tx/Rx

| | |
|---|---|
| Frequency 1H | 123.262000 MHz |
| Correction factor | 1 |
| Gain | High |
| Img. Scale Cor. | 1.000 |
| Reset | Off |
| ? Ref. amplitude 1H | 0.000 V |

## Physio - Signal1

| | |
|---|---|
| 1st Signal/Mode | ECG/Retro |
| Average cycle | 681 ± 105 ms |
| Average cycle | No Signal ms |
| Calculated phases | 25 |
| TR | 37.80 ms |
| Concatenations | 1 |
| Segments | 12 |
| Arrhythmia detection | None |

## Physio - Cardiac

| | |
|---|---|
| Tagging | None |
| Magn. preparation | None |
| Fat suppr. | None |
| Dark blood | Off |

## Physio - Cardiac

| | |
|---|---|
| FoV read | 380 mm |
| FoV phase | 75.0 % |
| Phase resolution | 80 % |
| Cine | On |
| Trajectory | Cartesian |
| View sharing | Off |
| Dummy heartbeats | 1 |

## Physio - PACE

| | |
|---|---|
| Resp. control | Breath-hold |
| Concatenations | 1 |

## Inline - Common

| | |
|---|---|
| Subtract | Off |
| Measurements | 1 |
| StdDev | Off |
| Save original images | On |

## Inline - Cardiac

| | |
|---|---|
| Inline Evaluation | Off |
| Magn. preparation | None |
| Contrasts | 1 |
| TE | 1.38 ms |
| TR | 37.80 ms |
| Save original images | On |

## Inline - MIP

| | |
|---|---|
| MIP-Sag | Off |
| MIP-Cor | Off |
| MIP-Tra | Off |
| MIP-Time | Off |
| Save original images | On |

## Inline - Composing

| | |
|---|---|
| Inline Composing | Off |
| Distortion Corr. | On |
| Mode | 2D |
| Unfiltered images | Off |

## Sequence - Part 1

| | |
|---|---|
| Introduction | Off |
| Dimension | 2D |
| Reordering | Linear |
| Asymmetric echo | Weak |
| Contrasts | 1 |
| Optimization | Min. TE TR |
| Multi-slice mode | Sequential |
| Echo spacing | 3.1 ms |
| Sequence type | Trufi |
| Bandwidth | 962 Hz/Px |

## Sequence - Part 2

| | |
|---|---|
| Define | Segments |
| Segments | 12 |
| Trufi delta freq. | 0 Hz |
| RF pulse type | Normal |
| Gradient mode | Fast |
| Excitation | Slice-sel. |
| Flip angle mode | Constant |
| Cine | On |

## Sequence - Assistant

| | |
|---|---|
| Mode | Min flip angle |

## Sequence - Assistant

| | |
|---|---|
| Min flip angle | 50 deg |
| Allowed delay | 0 s |

| \\USER\T1Map_cardio_OxfordC2P_ShMOLLI\Template_Clinical\Templates_VNE\Late_Gad_Trufi_psir |
|---|
| TA: 0:11 PM: REF Voxel size: 1.5×1.5×7.0 mmPAT: Off Rel. SNR: 1.00 : tfi |

## Properties

| | |
|---|---|
| Prio recon | Off |
| Load images to viewer | On |
| Inline movie | Off |
| Auto store images | On |
| Load images to stamp segments | On |
| Load images to graphic segments | On |
| Auto open inline display | Off |
| Auto close inline display | Off |
| Start measurement without further preparation | On |
| Wait for user to start | Off |
| Start measurements | Single measurement |

## Routine

| | |
|---|---|
| Slice group | 1 |
| Slices | 1 |
| Dist. factor | 20 % |
| Position | L36.0 A4.2 F37.4 mm |
| Orientation | S > T42.6 > C33.6 |
| Phase enc. dir. | A >> P |
| AutoAlign | --- |
| Phase oversampling | 0 % |
| FoV read | 380 mm |
| FoV phase | 61.7 % |
| Slice thickness | 7.0 mm |
| TR | 700.00 ms |
| TE | 1.19 ms |
| Averages | 1 |
| Concatenations | 1 |
| Filter | Raw filter, Distortion Corr.(2D), Prescan Normalize |
| Coil elements | BO1-3;SP1-3 |

## Contrast - Common

| | |
|---|---|
| TR | 700.00 ms |
| TE | 1.19 ms |
| Magn. preparation | Non-sel. IR |
| TI | 300 ms |
| Flip angle | 55 deg |
| Fat suppr. | None |
| Wrap-up Magn. | Suppress |

## Contrast - Dynamic

| | |
|---|---|
| Averages | 1 |
| Averaging mode | Short term |
| Reconstruction | Magnitude/Real |
| Measurements | 1 |
| Multiple series | Each measurement |

## Resolution - Common

| | |
|---|---|
| FoV read | 380 mm |
| FoV phase | 61.7 % |
| Slice thickness | 7.0 mm |
| Base resolution | 256 |
| Phase resolution | 72 % |
| Phase partial Fourier | Off |
| Trajectory | Cartesian |
| Interpolation | Off |

## Resolution - iPAT

| | |
|---|---|
| PAT mode | None |

## Resolution - Filter Image

| | |
|---|---|
| Image Filter | Off |
| Distortion Corr. | On |
| Mode | 2D |
| Unfiltered images | Off |
| Prescan Normalize | On |
| Unfiltered images | Off |
| Normalize | Off |
| B1 filter | Off |

## Resolution - Filter Rawdata

| | |
|---|---|
| Raw filter | On |
| Elliptical filter | Off |
| POCS | Off |

## Geometry - Common

| | |
|---|---|
| Slice group | 1 |
| Slices | 1 |
| Dist. factor | 20 % |
| Position | L36.0 A4.2 F37.4 mm |
| Orientation | S > T42.6 > C33.6 |
| Phase enc. dir. | A >> P |
| FoV read | 380 mm |
| FoV phase | 61.7 % |
| Slice thickness | 7.0 mm |
| TR | 700.00 ms |
| Multi-slice mode | Sequential |
| Series | Descending |
| Concatenations | 1 |

## Geometry - AutoAlign

| | |
|---|---|
| Slice group | 1 |
| Position | L36.0 A4.2 F37.4 mm |
| Orientation | S > T42.6 > C33.6 |
| Phase enc. dir. | A >> P |
| AutoAlign | --- |
| Initial Position | L36.0 A4.2 F37.4 |
| L | 36.0 mm |
| A | 4.2 mm |
| F | 37.4 mm |
| Initial Rotation | 26.97 deg |
| Initial Orientation | S > T |
| S > T | 42.6 |
| > C | 33.6 |

## Geometry - Saturation

| | |
|---|---|
| Fat suppr. | None |
| Wrap-up Magn. | Suppress |
| Special sat. | None |

## Geometry - Navigator

## Geometry - Tim Planning Suite

| | |
|---|---|
| Set-n-Go Protocol | Off |
| Table position | H |
| Table position | 0 mm |

### Geometry - Tim Planning Suite

| | |
|---|---|
| Inline Composing | Off |

### System - Miscellaneous

| | |
|---|---|
| Positioning mode | REF |
| Table position | H |
| Table position | 0 mm |
| MSMA | S - C - T |
| Sagittal | R >> L |
| Coronal | A >> P |
| Transversal | F >> H |
| Coil Combine Mode | Adaptive Combine |
| Save uncombined | Off |
| Matrix Optimization | Cardio |
| Coil Focus | Flat |
| AutoAlign | --- |
| Coil Select Mode | Default |

### System - Adjustments

| | |
|---|---|
| B0 Shim mode | Cardiac |
| B1 Shim mode | TrueForm |
| Adjust with body coil | Off |
| Confirm freq. adjustment | Off |
| Assume Dominant Fat | Off |
| Assume Silicone | Off |
| Adjustment Tolerance | Auto |

### System - Adjust Volume

| | |
|---|---|
| ! Position | L15.0 A45.0 H25.0 mm |
| ! Orientation | Sagittal |
| ! Rotation | 0.00 deg |
| ! A >> P | 170 mm |
| ! F >> H | 170 mm |
| ! R >> L | 170 mm |
| Reset | Off |

### System - Tx/Rx

| | |
|---|---|
| Frequency 1H | 123.262000 MHz |
| Correction factor | 1 |
| Gain | High |
| Img. Scale Cor. | 1.000 |
| Reset | Off |
| ? Ref. amplitude 1H | 0.000 V |

### Physio - Signal1

| | |
|---|---|
| 1st Signal/Mode | ECG/Trigger |
| Average cycle | 681 ± 105 ms |
| Average cycle | No Signal ms |
| Captured cycle | 681 ± 105 ms |
| Acquisition window | 700 ms |
| Trigger pulse | 2 |
| Trigger delay | 0 ms |
| TR | 700.00 ms |
| Concatenations | 1 |
| Segments | 38 |
| Phases | 1 |
| Adaptive Triggering | Off |

### Physio - Cardiac

| | |
|---|---|
| Tagging | None |
| Magn. preparation | Non-sel. IR |
| TI | 300 ms |
| Fat suppr. | None |
| Dark blood | Off |
| FoV read | 380 mm |

### Physio - Cardiac

| | |
|---|---|
| FoV phase | 61.7 % |
| Phase resolution | 72 % |
| Cine | Off |
| Trajectory | Cartesian |
| Dummy heartbeats | 1 |

### Physio - PACE

| | |
|---|---|
| Resp. control | Breath-hold |
| Concatenations | 1 |

### Inline - Common

| | |
|---|---|
| Subtract | Off |
| Measurements | 1 |
| StdDev | Off |
| Save original images | On |

### Inline - Cardiac

| | |
|---|---|
| Inline Evaluation | Off |
| Magn. preparation | Non-sel. IR |
| Contrasts | 1 |
| TE | 1.19 ms |
| TR | 700.00 ms |
| Save original images | On |

### Inline - MIP

| | |
|---|---|
| MIP-Sag | Off |
| MIP-Cor | Off |
| MIP-Tra | Off |
| MIP-Time | Off |
| Save original images | On |

### Inline - Composing

| | |
|---|---|
| Inline Composing | Off |
| Distortion Corr. | On |
| Mode | 2D |
| Unfiltered images | Off |

### Sequence - Part 1

| | |
|---|---|
| Introduction | Off |
| Dimension | 2D |
| Reordering | Linear |
| Asymmetric echo | Weak |
| Contrasts | 1 |
| Optimization | Min. TE |
| Multi-slice mode | Sequential |
| Echo spacing | 2.9 ms |
| Sequence type | Trufi |
| Bandwidth | 781 Hz/Px |

### Sequence - Part 2

| | |
|---|---|
| Define | Shots |
| Shots per slice | 3 |
| Segments | 38 |
| Trufi delta freq. | 0 Hz |
| RF pulse type | Fast |
| Gradient mode | Fast |
| Excitation | Slice-sel. |
| Flip angle mode | Constant |
| Cine | Off |

### Sequence - Assistant

| | |
|---|---|
| Mode | Min flip angle |
| Min flip angle | 40 deg |
